# Development of clinical phenotypes and biological profiles via proteomic analysis of trauma patients

**DOI:** 10.1186/s13054-022-04103-z

**Published:** 2022-08-06

**Authors:** Jotaro Tachino, Hisatake Matsumoto, Fuminori Sugihara, Shigeto Seno, Daisuke Okuzaki, Tetsuhisa Kitamura, Sho Komukai, Yoshiyuki Kido, Takashi Kojima, Yuki Togami, Yusuke Katayama, Yuko Nakagawa, Hiroshi Ogura

**Affiliations:** 1 Department of Traumatology and Acute Critical Medicine, Osaka University Graduate School of Medicine, 2-15, Yamada‑oka, Suita, Osaka 565-0871 Japan; 2 Core Instrumentation Facility, Immunology Frontier Research Center and Research Institute for Microbial Diseases, Osaka University, 3-3-1, Yamada‑oka, Suita, Osaka Japan; 3 Department of Bioinformatic Engineering, Graduate School of Information Science and Technology, Osaka University, 1-5 Yamada-oka, Suita, Osaka Japan; 4 Genome Information Research Center, Research Institute for Microbial Disease, Osaka University, 3-1 Yamada-oka, Suita, Osaka Japan; 5 Division of Environmental Medicine and Population Sciences, Department of Social and Environmental Medicine, Osaka University Graduate School of Medicine, 2-2, Yamada‑oka, Suita, Osaka Japan; 6 Division of Biomedical Statistics, Department of Integrated Medicine, Graduate School of Medicine, Osaka University, 2-2, Yamada‑oka, Suita, Osaka Japan; 7 Cybermedia Center, Osaka University, 5-1, Mihoga-oka, Ibaraki, Osaka Japan; 8 Facility of Informatics, Okayama University of Science, 1-1, Ridaicho, Kita-ku, Okayama, Okayama Japan

**Keywords:** Trauma, Phenotype, Inflammatory response, Personalized medicine

## Abstract

**Background:**

Trauma is a heterogeneous condition, and specific clinical phenotypes may identify target populations that could benefit from certain treatment strategies. In this retrospective study, we determined clinical phenotypes and identified new target populations of trauma patients and their treatment strategies.

**Methods:**

We retrospectively analyzed datasets from the Japan Trauma Data Bank and determined trauma death clinical phenotypes using statistical machine learning techniques and evaluation of biological profiles.

**Results:**

The analysis included 71,038 blunt trauma patients [median age, 63 (interquartile range [IQR], 40–78) years; 45,479 (64.0%) males; median Injury Severity Score, 13 (IQR, 9–20)], and the derivation and validation cohorts included 42,780 (60.2%) and 28,258 (39.8%) patients, respectively. Of eight derived phenotypes (D-1–D-8), D-8 (*n* = 2178) had the highest mortality (48.6%) with characteristic severely disturbed consciousness and was further divided into four phenotypes: D-8α, multiple trauma in the young (*n* = 464); D-8β, head trauma with lower body temperature (*n* = 178); D-8γ, severe head injury in the elderly (*n* = 957); and D-8δ, multiple trauma, with higher predicted mortality than actual mortality (*n* = 579). Phenotype distributions were comparable in the validation cohort. Biological profile analysis of 90 trauma patients revealed that D-8 exhibited excessive inflammation, including enhanced acute inflammatory response, dysregulated complement activation pathways, and impaired coagulation, including downregulated coagulation and platelet degranulation pathways, compared with other phenotypes.

**Conclusions:**

We identified clinical phenotypes with high mortality, and the evaluation of the molecular pathogenesis underlying these clinical phenotypes suggests that lethal trauma may involve excessive inflammation and coagulation disorders.

**Supplementary Information:**

The online version contains supplementary material available at 10.1186/s13054-022-04103-z.

## Background

The standardization of trauma care is being promoted, and research is being conducted worldwide to improve outcomes. However, 4.5 million cases of trauma-related deaths are reported annually worldwide [1]. The roles of coagulation, fibrinolysis, and immune reactions in trauma are well understood, but their application as potential therapeutic targets remains limited. The development of new treatments is complicated by the heterogeneity of trauma due to age, sex, comorbidities, injury type and degree, and complex pathophysiology. Therefore, correctly determining the effects of therapeutic interventions remains intractable. Studies using large-scale registry data have attempted to validate interaction effects to identify groups with fatal polytrauma and reported that specific combinations of injuries significantly affect patient outcomes [2]. Thus, in analyses that consider the effects of complex, nonlinear interactions may reveal the pathological conditions that interact with multiple factors.

Recent studies investigated new therapeutic targets by combining unsupervised learning and biological indicators in various diseases to elucidate potential sub-phenotypes [3–7]. Identifying trauma sub-phenotypes with poor outcomes and complex pathologies may enable the discovery of new therapeutic strategies and target populations. Recent advances in technology have allowed researchers to acquire comprehensive biomolecular information. Following trauma, damage-related molecular patterns bind to pattern recognition receptors expressed on immunocompetent cells, followed by activated intracellular transcription factors binding to nuclear DNA and promoting upregulated transcription and translation of target genes and a systemic inflammatory response. Thus, proteomic analysis of blood might broaden the understanding of the molecular pathology underlying trauma.

In this study, we identified latent clinical phenotypes in trauma patients, with the primary goal of identifying lethal clinical phenotypes with high mortality rates based on available clinical information. The secondary goal was to clarify the molecular pathology of the derived clinical phenotypes by analyzing biological data, including proteomic data.

## Methods

### Overview

**Fig. 1:**
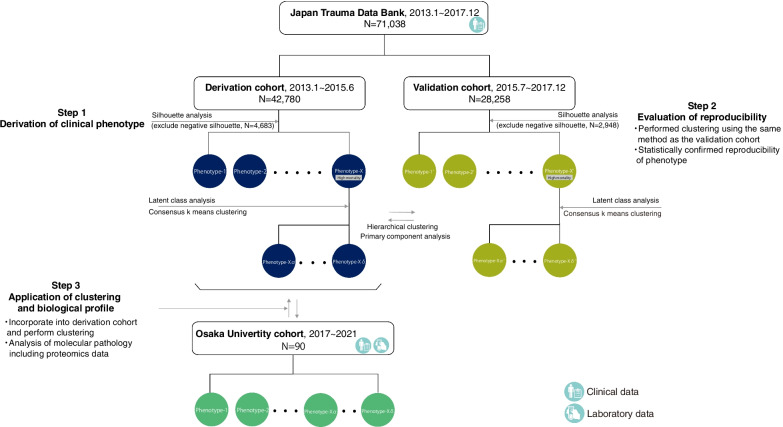
Outline of the study scheme

This study included two datasets and used several statistical approaches. The study scheme is outlined in Fig. 1. First, we divided the datasets from Japan Trauma Data Bank (JTDB) into two cohorts according to the registration period and identified clinical phenotypes from the derivation cohort using unsupervised clustering. We then derived the clinical phenotypes using the same clustering method in the validation cohort and subsequently analyzed the distribution of each component. We assessed the reproducibility and consistency of the two clinical phenotypes by performing hierarchical clustering analysis. After determining the correlation between the clinical phenotypes and biological markers of host response, we clustered trauma patients from another dataset into different clinical phenotypes and evaluated the molecular pathology of each cluster by analyzing serum proteomic data. This study was conducted according to the Declaration of Helsinki and approved by the Ethics Committee of Osaka University (IRB approval Nos. 16260, 21,211, and 885; Osaka University Critical Care Consortium Novel Omix Project; Occonomix Project).

### Data collection

We used JTDB data related to all blunt trauma patients. Patients who had non-direct transportation, suffered cardiopulmonary arrest on arrival, had an Injury Severity Score (ISS) of 75, were pregnant, or had significant missing data were excluded from the study. The primary outcome was all-cause mortality. In another dataset, we evaluated the association between clinical phenotypes and biological markers of host response for blunt trauma patients transferred to Osaka University Hospital from 2017 to 2021 (Osaka University cohort). In this cohort, a datasheet containing clinical data necessary (Additional file 1: Methods: Biological Correlates and Clinical Outcomes) for clustering was generated, and biological information was collected. After incorporating the Osaka University cohort into the derivation cohort and determining clusters for each case in the former cohort, biological profiling was performed.

### Candidate clinical variables for phenotyping

To generate a model applicable to early trauma care, we restricted the candidate variables to the following two conditions: (1) those related to trauma outcome and pathophysiology and (2) those included as the general information obtained during initial trauma care. Subsequently, 14 candidate variables were selected. We generated a data sheet and included the following information to understand the baseline characteristics of the patients: age, sex, existing comorbidities, vital signs on arrival, AIS code (AIS 90, update 98) [8], ISS [9], Revised Trauma Score [10], and a Trauma and Injury Severity Score and Probability of Survival (TRISS-PS) [11]. The ISS was calculated from the top three scores of the AIS of the six trunks classified by the AIS code.

### Statistical analysis

Based on our aims to (1) develop and evaluate new clinical phenotypes related to traumatic death and (2) understand the molecular pathology underlying the derived clinical phenotypes, we first assessed the distribution of the candidate variables and instances where they were absent. The 5-year trauma data were split to obtain a ratio of approximately 6:4 for the sample size used to derive and verify the phenotype (derivation: January 2013–June 2015; validation: July 2015–December 2017) [12]. To avoid multicollinearity, we excluded candidate variables with an absolute correlation coefficient > 0.5 [13]. We derived clinical phenotypes associated with trauma-related death and performed two-step clustering considering calculation cost. We calculated the appropriate number of clusters by the mean silhouette and *k*-means methods [14, 15]. After standardizing patient data, clustering was performed using the *k*-means algorithm based on the optimal number of clusters, and the silhouette coefficient was calculated according to Euclidean distance. Negative silhouettes were removed, and the survival of each phenotype was evaluated. The clinical phenotype with the highest mortality rate was then extracted and clustered a second time. For the second clustering, a group was derived using latent class analysis (LCA), where the Bayesian information criterion (BIC), appropriate size of each phenotype, and misclassification rate of each phenotype were evaluated to confirm the optimal phenotype number [16, 17]. The optimal class number was selected based on the largest BIC considering the misclassification rate and interpretability [Additional file 1: Methods: Latent class analysis (LCA) and Calculation of BIC with LCA] [6]. The proportion of patients assignable to a phenotype at the margin was determined as 45% to 55%. To confirm the robustness of the phenotype, consensus *k*-means clustering was performed using 14 variables (Additional file 1: Methods: Consensus K clustering) [18]. To determine the optimal number of phenotypes, we evaluated the number of patients included in each phenotype, clear separation of the consensus matrix heatmaps, characteristics of the consensus cumulative distribution function plots, and appropriate pairwise consensus values between the clusters (> 0.8). Additionally, we visualized t-distributed stochastic neighbor embedding (t-SNE) to confirm the reproducibility between LCA and consensus *k-*means clustering [Additional file 1: Methods: Data visualizing/t-Distributed Stochastic Neighbor Embedding (t-SNE) plot] [19]. Upon determining the clinical phenotype, we generated a complex heatmap, violin plots, and alluvial plots to visualize the distribution of clinical explanatory variables in order to evaluate the features of each clinical phenotype (Additional file 1: Methods: Data visualizing/Alluvial plot).

Clustering in the validation cohort was performed as described for the derivation cohort. We statistically evaluated the reproducibility as follows: 1) hierarchical clustering by principal component scores of each phenotype and 2) visualization of the centroid of each phenotype with size based on the number of patients by principal component analysis. Additionally, we collected clinical information to cluster patients who were transferred to the Department of Traumatology and Acute Critical Medicine at Osaka University Graduate School of Medicine from February 2017 to March 2021 and incorporated these data into the derivation cohort dataset, with clustering performed as described. The high-mortality group included all cases, whereas the remaining seven clinical phenotypes included ≤ 14 patients close to the centroid in each group.

To evaluate the biological characteristics of each phenotype, we procured general laboratory data of the patients using samples taken upon their arrival to the hospital and performed proteomic analysis of the serum collected within 72 h of injury using mass spectrometry (Additional file 1: Methods: Mass spectrometry). Volcano plot analysis was performed using the limma voom algorithm [20, 21] to identify differentially expressed proteins between the high-mortality phenotype and other phenotypes. Differential protein expression was defined as a false discovery rate < 0.2 and a fold change >|1.2|. Subsequently, Gene Ontology (GO) enrichment analysis was performed using the R package clusterProfiler [22]. Patient characteristics data are described as the mean (standard deviation) or median [interquartile range (IQR)]. Mann–Whitney U tests, analysis of variance, and Kruskal–Wallis tests were used to compare continuous data, and the chi-square test was performed for categorical data. The threshold of statistical significance was *p* < 0.05 according to a two-sided test. No adjustment was made to the type I error rate by multiple comparisons; therefore, these results should be considered exploratory.

All statistical analyses were performed using R (v4.0.2; https://www.r-project.org/). Some analyses were performed on a supercomputer (OCTOPUS; Osaka University Cybermedia cenTer Over-Petascale Universal Supercomputer). This study followed the STROBE guidelines [23].

## Results

### Study population

From 2013 to 2017, 158,918 trauma patients were enrolled in JTDB. Of these, 12,565 non-blunt trauma patients and 75,315 patients who did not meet the inclusion criteria were excluded, leaving 71,038 patients for the final analyses (Additional file 1: Fig. S1). Patients admitted from January 2013 to June 2015 were included in the derivation cohort (*n* = 42,780; 60.2%), and patients admitted from July 2015 to December 2017 were included in the validation cohort (*n* = 28,258; 39.8%) (Fig. 1). Baseline characteristics of all patients are listed in Table 1. Overall, the median age was 63 years (IQR 40–78 years), median ISS was 13 (IQR 9–20), median TRISS-PS was 0.97 (IQR 0.93–0.99), and the in-hospital mortality rate was 5.5%. The biological profile cohort included 171 patients, with a median age of 50 years (IQR 34–71 years), a median ISS of 17 (IQR 6–26), and median TRISS-PS of 0.97 (IQR 0.84–0.99), and an in-hospital mortality rate of 5.8%. This cohort was incorporated into the derivation cohort, and clustering analysis was performed using the same method. Additionally, we analyzed biological data for 90 individuals whose coordinates were close to the centroid of each phenotype.

**Table 1:** Baseline characteristics of the patients in all three cohorts

Variables	Derivation cohort	Validation cohort	Osaka University cohort	Overall
Number of patients	42,780	28,258	171	71,209
Age, years, median [IQR]	62 [38–77]	65 [42–79]	50 [34–71]	63 [40–78]
Male gender, no. (%)	27,545 (64.4)	17,934 (63.5)	128 (74.9)	45,607 (64.0)
CPS, median [IQR]	1 [1–1]	1 [1, 2]	0 [0–1]	1 [1–1]
Respiratory rate, median [IQR]	20 [17–24]	20 [17–24]	21 [18–26]	20 [17–24]
Heart rate, median [IQR]	83 [72–96]	83 [72–96]	90 [74–108]	83 [72–96]
Systolic blood pressure, median [IQR]	138 [119–158]	139 [120–160]	139 [119–161]	138 [119–159]
Body temperature (℃), median [IQR]	36.5 [36.0–36.9]	36.5 [36.1–36.9]	36.5 [36.0–36.9]	36.5 [36.0–36.9]
Glasgow Coma Scale, median [IQR]	15 [14, 15]	15 [14, 15]	14 [12–15]	15 [14, 15]
Head & Cervical AIS, median [IQR]	1 [0–4]	1 [0–4]	1 [0–4]	1 [0–4]
Face AIS, median [IQR]	0 [0–0]	0 [0–0]	0 [0–0]	0 [0–0]
Chest AIS, median [IQR]	0 [0–2]	0 [0–2]	0 [0–3]	0 [0–2]
Abdomen AIS, median [IQR]	0 [0–0]	0 [0–0]	0 [0–1]	0 [0–0]
Extremities AIS, median [IQR]	2 [0–3]	2 [0–3]	1 [0–2]	2 [0–3]
External AIS, median [IQR]	0 [0–0]	0 [0–0]	0 [0–0]	0 [0–0]
ISS, median [IQR]	13 [9–20]	13 [9–20]	17 [6–26]	13 [9–20]
RTS, median [IQR]	7.84 [7.55–7.84]	7.84 [7.84–7.84]	7.84 [6.90–7.84]	7.84 [7.55–7.84]
TRISS-PS, median [IQR]	0.97 [0.93–0.99]	0.97 [0.93–0.99]	0.97 [0.84–0.99]	0.97 [0.93–0.99]
Survival, no. (%)	40,344 (94.3)	26,753 (94.7)	161 (94.2)	67,258 (94.5)

*IQR* interquartile range, *CPS* Charlson polypharmacy scale, *AIS* abbreviated injury scale, *ISS* injury severity scale, *RTS* revised trauma score, *TRISS-PS*, Trauma and Injury Severity Score and Probability of Survival

### Derivation of clinical trauma phenotypes

We examined correlations among 14 candidate variables and found that there were no variables with absolute correlation coefficients > 0.5 (Additional file 1: Fig. S2). In the derivation cohort, the optimal number of phenotypes was identified as 8 according to the mean silhouette and the *k*-means method (Additional file 1: Figs. S3 and 4). Additional file 1: Table S1 shows the characteristics of 38,097 patients after removing the negative silhouettes (Additional file 1: Fig. S5). Subsequently, we identified the clinical phenotypes with high mortality rates (Fig. 1 and Additional file 1: Table S1) and performed LCA on the phenotype with the highest mortality rate (Additional file 1: Fig. S6). The BIC for the LCA model increased continuously with the number of classes, with changes in the BIC decreasing when the number of classes was ≥ 4. Overall, the four-class model was the best-fitting model because of its low misclassification rate and interpretability (Additional file 1: Fig. S7) and showed strong separation in the likelihood of membership for patients assigned to a given phenotype rather than to other phenotypes (Additional file 1: Figs. S8 and 9). The discriminative power of each variable in LCA was subsequently confirmed (Additional file 1: Fig. S10). Additional file 1: Table S2 shows the patient number and baseline characteristics of the four clinical phenotypes derived from the high-mortality cluster in the derivation cohort, with the clinical features of the high-mortality group shown in violin and alluvial plots (Additional file 1: Figs. S11 and 12). Severe head trauma with impaired consciousness and a Glasgow Coma Scale ≤ 8 were common in the high-mortality group. The clinical phenotype D-8α is comprised predominantly of young individuals with chest, limb, and pelvic trauma complications (29.2% mortality). Phenotype D-8β mainly included elderly people with head trauma and hypothermia on hospital arrival (46.6% mortality). Clinical phenotype D-8γ had the largest number of patients in the high-mortality group, with the highest mortality rate (56.6%) observed in the elderly with severe head trauma. Phenotype D-8δ was characterized by polytrauma and the highest ISS, with many individuals with this phenotype having chest, extremity, and pelvic trauma. The mortality rate for this phenotype was 51.6%, with the predicted mortality rate considerably higher than the actual mortality rate.

### Evaluation of reproducibility

**Fig. 2:**
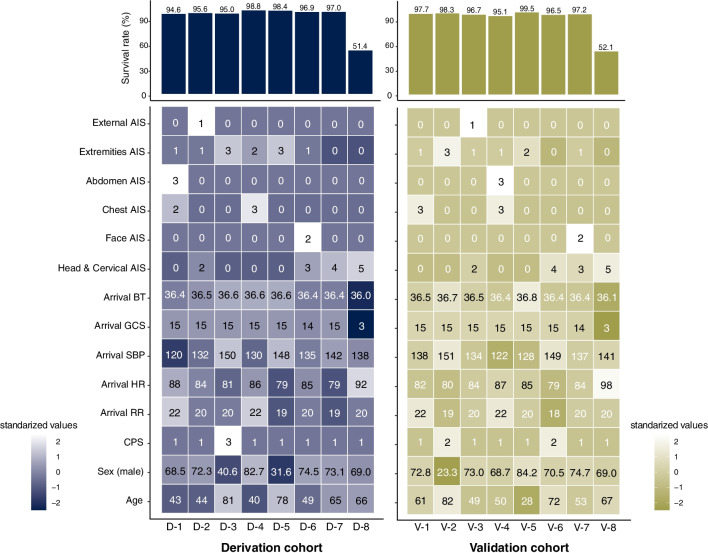
Complex heatmap with the distribution of survival rates and variables in each clinical phenotype. Heatmap showing the distribution of survival rates and variables in each clinical phenotype. The upper panel shows the survival rate for each clinical phenotype in bar graphs. The heatmap in the bottom panel shows each variable (standardized and colored). The number in the cell is the median (sex is shown as a percentage of males). AIS, Abbreviated Injury Scale; BT, body temperature; GCS, Glasgow Coma Scale; SBP, systolic blood pressure; HR, heart rate; RR, respiratory rate; CPS, Charlson Polypharmacy Scale

**Fig. 3:**
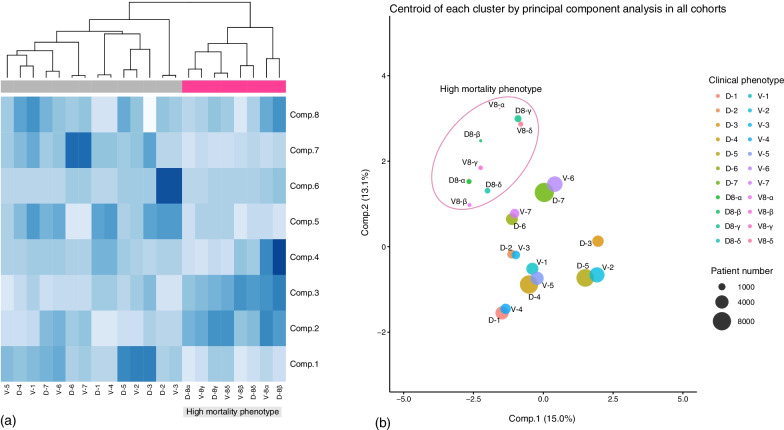
Evaluation of reproducibility for clinical phenotypes. **a** Hierarchical cluster analysis for principal component scores of centroids of each phenotype. Phenotype analysis, silhouette analysis, and latent class analysis were used. Patient data with phenotype information were combined into a single data sheet, PCA was performed, principal component scores for each phenotypic centroid were calculated, and hierarchical cluster analysis was performed. **b** PCA for the centroid of each phenotype. The horizontal axis is the first principal component axis, and the vertical axis is the second principal component axis. Plot size indicates the number of patients. The distribution of cluster 8, which is strongly associated with death, was clearly different from that of clusters 1–7. There was no dissociation in the position of the clusters between the derivation and validation cohorts, suggesting that homology between the cohorts was maintained. PCA, principal component analysis

In the validation cohort, clinical phenotypes were derived from silhouette analysis and LCA. The optimal number of phenotypes was 8 when the mean silhouette method and the *k*-means method were used, as was the case for the derived cohort (Additional file 1: Fig. S13). The LCA for high-mortality phenotypes optimally classified the phenotypes into four phenotypes based on the BIC results. Moreover, consensus *k*-means clustering for the high-mortality group confirmed its robustness, suggesting that the t-SNE results were comparable with the LCA classification (Additional file 1: Fig. S14). We performed the same clustering for the validation cohort (Additional file 1: Figs. S16–27 and Tables S3 and 4). Figure 2 shows the survival rate distribution and each variable for the phenotypes generated in the derivation and validation cohorts. The high-mortality phenotype (D-8/V-8) was characterized by severely impaired consciousness, lower body temperature, and a higher degree of severe head trauma. Survival analysis revealed that this phenotype (D-8/V-8) showed decreased survival over time (Additional file 1: Figs. S15 and 28). Hierarchical clustering based on principal component scores confirmed that high-mortality clusters were statistically paired (Fig. 3a). We subsequently plotted the principal component coordinates of the calculated centroids according to the numbers of patients and visualized pairs with similar phenotypes in each cohort (Fig. 3b and Additional file 1: Fig. S29).

### Correlation of clinical phenotypes with biomarker profiles

**Fig. 4:**
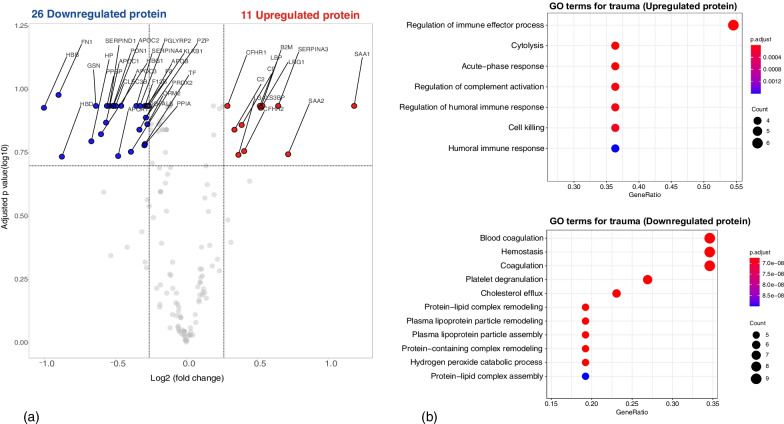
Molecular pathophysiological comparison of high-mortality phenotypes and other phenotypes. **a** Serum proteins differentially expressed between the high-mortality phenotype and other phenotypes (volcano plot). Red indicates proteins with upregulated expression, and blue indicates proteins with downregulated expression. The names of proteins demonstrating significantly different levels of expression are shown (false discovery rate < 0.2), as are those with fold changes >|1.2|. **b** Gene Ontology enrichment analysis identified 256 reliable proteins. Dots represent the top 12 enriched biological process terms. The color of the dots indicates significance (Benjamini–Hochberg adjusted *p* value), and their size indicates the number of differentially expressed proteins in the list of significant proteins associated with the GO term

**Fig. 5:**
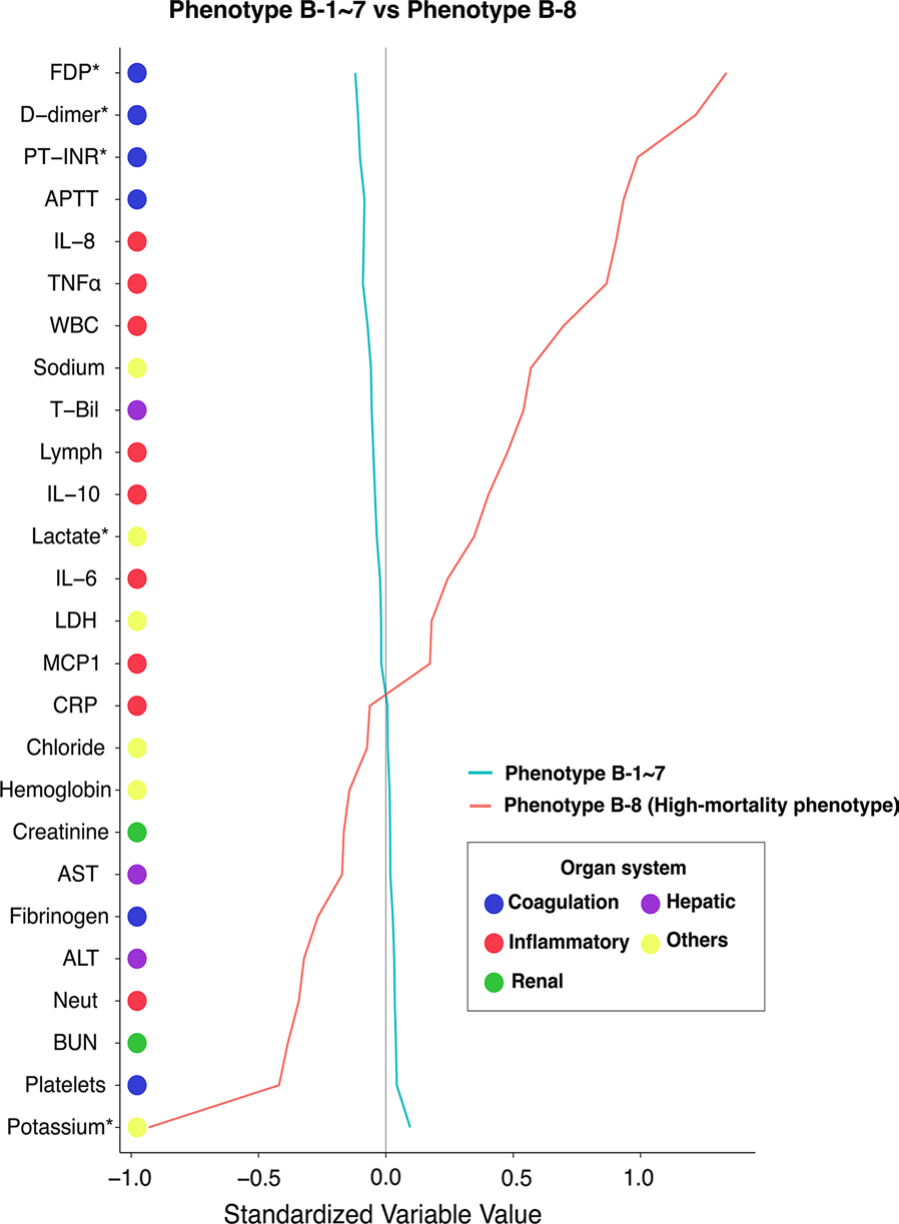
Comparison of variables contributing to clinical phenotype in the Osaka University cohort. A line plot was created to visualize the differences in general blood-derived variables between the high-mortality phenotype and other phenotypes. Each indicator is color-coded according to the organ system. Variables were standardized to scale all means to 0 and SDs to 1. Standardized variables (X-axis) with a value of 1 had a mean value with an SD > 1, which is higher than the mean value of the entire phenotype. Asterisks indicate significant differences in Mann–Whitney U tests. FDP, fibrinogen/fibrin degradation products; PT-INR, prothrombin time–international normalized ratio; APTT, activated partial thromboplastin time; IL, interleukin; TNF-α, tumor necrosis factor-α; WBC, white blood cell; T-bil, total bilirubin; Lymph: lymphocyte; LDH, lactate dehydrogenase; MCP1, monocyte chemotactic protein 1; CRP, C-reactive protein; AST, aspartate transaminase; ALT, alanine transaminase; Neut, neutrophil; BUN, blood urea nitrogen; SD, standard deviation

The patient characteristics of the cohort for the biological profile are shown in Additional file 1: Table S5. We removed decoy proteins and immunoglobulins and used 256 proteins according to their standing according to the total exponentially modified protein abundance index summation [24]. Among the differentially expressed proteins, expression levels of 11 and 26 proteins were significantly upregulated and downregulated, respectively (Fig. 4a and Additional file 1: Tables S6 and 7). Characteristics of patients with high-mortality phenotypes in all three cohorts were compared and are presented in Table 2. GO enrichment analysis showed that clinical phenotype B-8 (equivalent to phenotype D-8/V-8) exhibited excessive inflammation, including enhanced acute inflammatory response and dysregulated complement activation pathways, and impaired coagulation, including downregulated coagulation and platelet degranulation pathways (Fig. 4b). When continuous variables were ranked according to the standardized mean difference between phenotypes (Fig. 5), phenotype B-8 showed higher laboratory values related to coagulation, fibrinolysis, and inflammation and lower values for fibrinogen and platelets relative to the other phenotypes.

**Table 2:** Comparison of characteristics of patients with high-mortality phenotypes in each cohort

Clinical phenotype	D-8	V-8	B-8	Overall	p value
Number of patients	2178	1241	9	3428	
Age, years, median [IQR]	66 [46–77]	67 [47–79]	66 [52–79]	66 [46–78]	0.122
Male gender, no. (%)	1502 (69)	856 (69)	9 (100)	2367 (69)	0.132
CPS (median [IQR])	1 [1–1]	1 [1–1]	1 [0–1]	1 [1–1]	< 0.001
Respiratory rate, median [IQR]	20 [16–25]	20 [16–25]	21 [17–27]	20 [16–25]	0.793
Heart rate, median [IQR]	92 [76–113]	98 [80–118]	108 [98–126]	94 [78–115]	< 0.001
Systolic blood pressure, median [IQR]	138 [109–167]	141 [111–169]	169 [152–205]	140 [110–168]	0.029
Body temperature, median [IQR]	36.0 [35.3–36.5]	36.1 [35.5–36.6]	35.8 [35.1–36.4]	36.0 [35.4–36.5]	< 0.001
Glasgow coma scale, median [IQR]	3 [3–6]	3 [3–5]	3 [3–3]	3 [3–6]	0.004
Head and Cervical AIS, median [IQR]	5 [4, 5]	5 [4, 5]	5 [5–5]	5 [4, 5]	0.002
Face AIS, median [IQR]	0 [0–0]	0 [0–0]	0 [0–0]	0 [0–0]	0.662
Chest AIS, median [IQR]	0 [0–3]	0 [0–3]	0 [0–0]	0 [0–3]	0.003
Abdomen AIS, median [IQR]	0 [0–0]	0 [0–0]	0 [0–0]	0 [0–0]	0.229
Extremities AIS, median [IQR]	0 [0–2]	0 [0–2]	1 [0–2]	0 [0–2]	0.748
External AIS, median [IQR]	0 [0–0]	0 [0–0]	0 [0–0]	0 [0–0]	< 0.001
ISS, median [IQR]	25 [21–34]	26 [25–36]	29 [25–30]	25 [22–35]	< 0.001
RTS, median [IQR]	4.09 [4.09–5.21]	4.09 [4.09–5.03]	4.09 [4.09–4.09]	4.09 [4.09–5.03]	0.102
TRISS-PS, median [IQR]	0.45 [0.26–0.67]	0.40 [0.21–0.63]	0.35 [0.21–0.39]	0.43 [0.24–0.65]	< 0.001
Survival, no. (%)	1119 (51.4)	647 (52.1)	5 (55.6)	1771(51.7)	0.888

*IQR* interquartile range, *CPS* Charlson polypharmacy scale, *AIS* abbreviated injury scale, *ISS* injury severity scale, *RTS* revised trauma score, *TRISS-PS* Trauma and Injury Severity Score and Probability of Survival

## Discussion

This analysis revealed 11 tentative, mutually exclusive clinical phenotypes that are multidimensional and exhibit differential patient characteristics along with distinct laboratory data and patterns of organ damage according to the injured region. The frequency and characteristics of the clinical phenotypes were confirmed as robust and reproducible using different cohorts and machine learning methods. Furthermore, biological analysis indicated that the high-mortality phenotype showed excessive inflammation and coagulation dysfunction.

The findings suggest that these phenotypes can be evaluated during the initial care of trauma patients to help develop treatment strategies for each respective phenotype and establish inclusion criteria for future clinical trials. Previous studies subgrouped trauma patients according to patient characteristics and injured organs to search for risk factors [25–27]; however, no study has comprehensively captured all trauma patients using machine learning approaches to search for new target populations in trauma treatment and evaluate them biologically. Machine learning is advantageous for variable selection and modeling, as it considers complex interaction effects and nonlinearity with outcomes [28, 29].

High-mortality groups exhibited a high degree of impaired consciousness and were further divided into a severe traumatic brain injury group and a polytrauma group according to reasonable clinical parameters. In trauma epidemiology, associations between combinations of injured regions and trauma-related mortality have been examined using a logistic model that included interaction effects and demonstrated significant interactions with mortality in head–chest and chest–pelvic/extremities injuries [2]. In the present study, clustering analysis revealed that injuries to the chest, pelvis, and extremities in the polytrauma group were associated with a high mortality rate. Moreover, the high-mortality group showed excessive inflammation, including a dysregulated acute inflammatory response and complement activation pathway, and coagulation abnormalities, which may play a major role in trauma death.

This study has two clinical implications. First, we used early trauma care data to identify clinical phenotypes with high mortality, identifying populations that may benefit from early intervention. Selecting sub-phenotypes of patients at high risk of poor outcomes and incorporating them into clinical trials is referred to as prognostic enrichment [30, 31]. However, no previous report has identified sub-phenotypes in early trauma stages or attempted new clinical trials, suggesting that the data presented here may reveal new therapeutic target populations and treatment strategies. Additionally, this technique may help clinicians predict potential trauma-related deaths and treat these patients appropriately. Second, we considered phenotypic pathological features from biological data, which enhanced the understanding of the endotypes of trauma patients. Excessive inflammation and coagulation disorders in clinical phenotype D-8/V-8 suggest the possibility of using preemptive treatment upon confirmation of the phenotype from initial clinical data. These findings may constitute a breakthrough for developing new trauma treatment strategies and therapeutic agents.

This study has a few limitations. First, this is a retrospective study, which has inherent limitations, as unmeasured confounding factors may affect trauma mortality. Second, with AIS coding, only the maximum value is adopted, even when there are multiple injuries in the same region. Third, we only used daily clinical data available from the electronic health records to identify clinical phenotypes. The JTDB datasets used in this study did not include blood test data; therefore, information on characteristics such as lactate levels and blood coagulation could not be used to derive clinical phenotypes. Moreover, future improvements in testing capabilities will likely help reveal biological indicators early in trauma care, which could result in completely different clinical phenotypes from those reported here. Fourth, the studied data were obtained only from Japanese patients. Therefore, the results may not be generalizable to other cohorts because of differences in trauma patient characteristics and medical practice across countries and regions. Fifth, the small sample size of the Osaka University cohort used for the biological profile may be statistically insufficient. Comparison of high-mortality phenotypes in each cohort showed significant differences in factors such as heart rate and body temperature. However, demographic data of the high-mortality phenotypes in each cohort were similar, and the significant differences could be attributed to the small sample size of the Osaka University cohort. Sixth, the body undergoes drastic changes after trauma, especially during the acute phase. In this study, the median proteomic sampling time was 1 h (IQR 0.8–1.7 h), with a maximum of 56 h after injury. Thus, the variation in blood sample collection time may have affected the results of proteomic analysis.

## Conclusions

In summary, this retrospective analysis using a nationwide trauma cohort for the Japanese population classified all trauma into 11 clinical phenotypes based on available clinical information acquired during the early stage of trauma care. We identified clinical phenotypes with high mortality, with the evaluation of the molecular pathogenesis of the derived clinical phenotypes suggesting that lethal trauma involves excessive inflammation and coagulation disorders.

## Supplementary Information

**Additional file 1**. Supplemental Digital Contents.

## Data Availability

Jotaro Tachino and Hisatake Matsumoto had full access to all the data in the study and take responsibility for the integrity of the data and the accuracy of the data analysis. The datasets used and/or analyzed during the current study are available from those authors on reasonable request.

## Abbreviations

AIS: Abbreviated injury scale
BIC: Bayesian information criterion
GO: Gene Ontology
IQR: Interquartile range
ISS: Injury Severity Score
JTDB: Japan Trauma Data Bank
LCA: Latent class analysis
t-SNE: T-distributed stochastic neighbor embedding
TRISS-PS: Trauma and Injury Severity Score and Probability of Survival
