# Supplementary material for: Development of clinical phenotypes and biological profiles via proteomic analysis of trauma patients
Source: Crit Care. 2022 Aug 6;26:241. doi: 10.1186/s13054-022-04103-z (PMC9357328; doi:10.1186/s13054-022-04103-z)
Supplement: Supplementary file 1 — Additional file 1. Supplemental Digital Contents. [file 13054_2022_4103_MOESM1_ESM.pdf]

Supplemental Digital Contents

Development of clinical phenotypes and biological profiles of trauma patients via proteome analysis

Contents

Database 4

Japan Trauma Data Bank 4

Definitions of variables 4

Supplementary Methods 5

Latent class analysis (LCA) 5

Calculation of BIC with LCA 5

Consensus K clustering 5

Data visualizing 6

Biological Correlates and Clinical Outcomes 6

Mass spectrometry 6

Supplementary References 8

Supplementary Tables 9

Supplemental Table 1. Silhouette plot clustering of patient characteristics in the derivation cohort 9

Supplemental Table 2. Characteristics of patients in high-mortality cluster in the derivation cohort 11

Supplemental Table 3. Silhouette plot clustering of patient characteristics in the validation cohort 12

Supplemental Table 4. Characteristics of patients in high-mortality cluster in the validation cohort 13

Supplemental Table 5. Patient characteristics in the cohort for biological profile 14

cal profile (continued) 15

Supplemental Table 6. Significantly regulated plasma proteins identified using limma voom algorithm 17

Supplemental Table 7. GO enrichment analysis results 21

Supplementary Figures 22

Supplemental Figure.1 Flowchart of patient selection 22

Supplemental Figure.2 Heatmap of correlation between clinical variables for phenotyping 23

Supplemental Figure.3 Optimal number of clusters in the derivation cohort (average silhouette width) 24

Supplemental Figure.4 Optimal number of clusters in the derivation cohort (elbow plot by k-means) 25

Supplemental Figure.5 Silhouette plot in the derivation cohort (excluding the negative silhouette) 26

Supplemental Figure.6 Outline of derivation cohort analysis 27

Supplemental Figure.7 Latent class analysis for high-mortality phenotype in the derivation cohort (Bayesian information criterion analysis) 28

Supplemental Figure.8 Probability of misclassification determined using latent class clustering for high-mortality group in the derivation cohort 29

Supplemental Figure.9 Probability of assignment for phenotype members and unassigned members determined using latent class analysis in the derivation cohort 30

Supplemental Figure.10 Discriminative power of each variable in the latent class analysis (derivation cohort) 32

Supplemental Figure.11 Distribution of variables for each clinical phenotype (high-mortality group, derivation cohort) 33

Supplemental Figure.12 Alluvial plot showing distribution of clinical phenotypes in the derivation cohort 35

Supplemental Figure.13 Consensus *k* clustering results in the derivation cohort......38

Supplemental Figure.14 t-SNE plot of clinical phenotype assignments in the derivation cohort 39

Supplemental Figure.15 Kaplan-Meier plot for the derivation cohort......40

Supplemental Figure.16 Optimal number of clusters in the validation cohort (average silhouette width)......41

Supplemental Figure.17 Optimal number of clusters in the validation cohort (elbow plot by k-means)......42

Supplemental Figure.18 Silhouette plot for the validation cohort (excluding the negative silhouette)......43

Supplemental Figure.19 Outline of validation cohort analysis......44

Supplemental Figure.20 Latent class analysis for high-mortality phenotype in the validation cohort (Bayesian information criterion analysis) 45

Supplemental Figure.21 Probability of misclassification determined using latent class clustering for the high-mortality group in the validation cohort......46

Supplemental Figure.22 Probability of assignment for phenotype members and unassigned members determined using latent class analysis in the validation cohort 47

Supplemental Figure.23 Discriminative power of each variable in the latent class analysis (validation cohort) 49

Supplemental Figure.24 Distribution of variables for each clinical phenotype (high-mortality group, validation cohort)......50

Supplemental Figure.25 Alluvial plot showing distribution of clinical phenotypes in the validation cohort......52

Supplemental Figure.26 Consensus *k* clustering results in the validation cohort......55

Supplemental Figure.27 t-SNE plot of clinical phenotype assignments in the validation cohort......56

Supplemental Figure.28 Kaplan-Meier plot for the validation cohort 57

Supplemental Figure.29 Centroid of each cluster determined using principal component analysis in all cohorts......58

Database

Japan Trauma Data Bank

The Japan Trauma Data Bank (JTDB) is a nationwide trauma registry introduced in Japan in 2003 that contains data from 272 hospitals as of March 2018. The JTDB was established by the Japanese Association for the Surgery of Trauma (Trauma Registry Committee) and the Japanese Association for Acute Medicine (Committee for Clinical Care Evaluation) to improve and assure the quality of trauma care in Japan. Data are continuously recorded, shared via the Internet, and stored on a data server at the Association for Japan Trauma Care and Research. Patients suspected of having an injury with an Abbreviated Injury Scale (AIS) score of 3 or greater are registered mainly from tertiary care and emergency centers. The patients are followed up until discharge from the hospital or death.

Definitions of variables

Information about age, gender, and underlying disease (comorbidity-polypharmacy score [CPS] calculated based on the sum of underlying conditions[1,2]) was obtained as patient baseline characteristics. We also obtained information regarding vital signs from the patients on arrival, such as respiratory rate, heart rate, systolic blood pressure, Glasgow Coma Scale (GCS), [3,4] and body temperature. For information on the location and severity of the trauma, we obtained AIS codes for the six portions (head and neck, face, chest, abdomen, extremities and pelvis, and body surface) required for calculating the Injury Severity Score (ISS). In addition, to understand patient characteristics, we obtained information on the presence or absence of blood transfusion within 24 hours, the presence or absence of emergency surgery, ISS, Revised Trauma Score (RTS), Trauma and Injury Severity Score-Probability of Survival (TRISS Ps), and in case of death, the time from injury to death.

Supplementary Methods

Latent class analysis (LCA)

LCA can be used to determine the optimal number of clusters and to obtain a cluster assignment for each subject; LCA generates a posterior probability for each subject-cluster combination that represents the likelihood that the subject belongs to that cluster. The posterior probability ranges from 0 to 1. For a given subject and a given cluster, the higher the posterior probability, the more likely it is that this subject belongs to this cluster. The subject is assigned to the cluster with the highest posterior probability. We determined the optimal number of clusters by combining the following criteria: (1) a larger Bayesian information criterion, (2) an appropriate sample size within a cluster, (3) a higher posterior probability of group assignment, and clinical characteristics of the cluster. The discriminative power of each variable used for clustering was calculated as the log of the ratio of the probability that the variable was relevant to clustering to the probability that it was irrelevant to clustering[5].

Calculation of BIC with LCA

Typically, the lowest BIC is the best fit model and is calculated as in equation (1):

$$\mathrm{BIC} = -2\ln \mathrm{L}(\hat{\theta}) + \mathrm{k}\ln(\mathrm{n}) \cdots (1)$$

where $\mathrm{L}(\hat{\theta})$ is the maximized value of the likelihood function, n is the number of data points, and k is the number of parameters estimated. However, the BIC in model-based clustering, as popularized by the VarSelLCM package in R, is calculated differently as shown in equation (2).

$$\mathrm{BIC} = \ln \mathrm{L}(\hat{\theta}) - 0.5\mathrm{k}\ln(\mathrm{n}) \cdots (2)$$

Overall, the BIC introduces a penalty term for the number of parameters in the model, thus selecting models with better fit. In this study, BIC was calculated using equation (2); therefore, the largest BIC is the optimal number of classes.

Consensus K clustering

Consensus clustering is a partitioning approach in which the clustering framework incorporates the results of multiple runs of an inner-loop clustering algorithm on subsampled subjects[6]. The advantage of consensus clustering is that it helps determine the optimal number of clusters and can be used to assess cluster stability. For each given number of clusters, a consensus matrix is created as a heat map. By varying the number of clusters and comparing the heat maps, one can visually determine the suitability for the data. Ideally, the clusters found should be stable across multiple clustering runs and there should be a clear separation on the heat map indicating that the optimal number of clusters was selected. Consensus cumulative density function (CDF) plots and cluster consensus plots were also used to confirm cluster number selection and cluster stability. The consensus CDF is a plot of the cumulative density function against the consensus value for each given number of clusters. The optimal number of clusters is represented by a CDF plot with a flat plateau until the first step is close to 0 and then the second step is close to 1. This indicates that the consensus values are dominated by numbers close to 0 and 1, indicating good

consensus across multiple inner loop algorithm runs. The cluster consensus plots show the average consensus value for all pairs within each cluster. The data were standardized, and the consensus k-means method was applied to (i) determine the optimal number of clusters and (ii) cluster assignment for each subject. The results of the consensus k-means could be summarized in three plots: a consensus matrix heat map, a consensus CDF plot, and a cluster consensus plot. The optimal number of clusters was assessed based on the combination of these plots, class size, and clinical characteristics. The position of consensus clustering in this study was as a sensitivity analysis of LCA.

Data visualizing

Alluvial plot

Alluvial plots were created to visualize clinical features by phenotype. These plots were created using the Alluvial package in R. Phenotypes are grouped in the leftmost column and arranged by proportion. Patients are grouped by phenotype, with ribbons connecting clinical characteristics. Bold ribbons indicate that more subjects fall into a particular phenotype or range.

t-Distributed Stochastic Neighbor Embedding (t-SNE) plot

t -SNE is a method that uses nonparametric, nonlinear dimensionality reduction to enable visualization of high-dimensional data sets. The method assigns weights to each of the modeling variables to create a two-dimensional composite eigenvector representing the gradient in the data. In our study, we created a two-dimensional t-SNE plot to represent the overall structure of the data. We also color-coded the patients belonging to the phenotypes. These plots were created in R using the tsne package.

Biological Correlates and Clinical Outcomes

For basic clinical blood data, the following instruments were used: Blood Count XN-9000 (Sysmex, Kobe, Japan), Biochemistry JCA-BM6050 (JEOL, Tokyo, Japan), and Coagulation CS-5100 (Sysmex). TNF-α, IL-6 and IL-8, and MCP-1 protein levels were measured by ELISA (all from DuoSet R&D Systems, Minneapolis, MN, USA).

Mass spectrometry

The serum protein was precipitated by adding methanol and chloroform and dissolved into PTS solution. The protein solution was reduced with dithiothreitol (DTT), followed by alkylation with iodoacetamide, digestion by trypsin, and purification with a C18 tip (GL Sciences, Tokyo, Japan). The trypsinized and purified solution was subjected to LC-MS/MS, with a Bruker TEN column (Bruker, Billerica, MA, USA) on a Nano Elute nanoLC system coupled with timsTOF Pro mass spectrometer (Bruker). The column temperature was set to 50.0℃. The mobile phase consisted of water containing 0.1% formic acid (solvent A) and acetonitrile containing 0.1% formic acid (solvent B). Peptides were eluted by the gradient setting of 2-35%B for 18 min at a flow rate of 500 nL/min. The mass scanning range was set to 300–2000 m/z, and the ion mobility revolution mode was set to custom with a range of 0.85–1.30 Vs/cm^2. The ion spray

voltage was set to 1.6 kV in positive ion mode. MS/MS spectra were acquired by automatic switching between MS and MS/MS modes. Bruker Data Analysis software was used for the processing of mass spectrometry data. Peptides were identified by a database search using MASCOT Server (ver.2.7, Matrix Science). Precursor mass tolerance was set to 15 ppm, and fragment mass tolerance was set to 0.05 Da. Carbamidomethylation of cysteine was set as a static modification, and oxidation of methionine, acetyl of protein N-term, deamination of N, Q were set as variable modifications. The Swiss-Prot database was used for Mascot search, and the taxonomy was limited to *Homo sapiens*. The search results were summarized using Scaffold (Proteome software), quantified using the exponentially modified protein abundance index (emPAI),[7] and exported in the CSV format for further analysis. DECOY proteins and immunoglobulins were removed, and the top 256 proteins of the total emPAI summation were used.

Supplementary References

[1] Evans DC, Gerlach AT, Christy JM, et al. Pre-injury polypharmacy as a predictor of outcomes in trauma patients. *Int J Crit Illn Inj Sci*. 2011;1(2):104-109.

[2] Evans DC, Cook CH, Christy JM, et al. Comorbidity-polypharmacy scoring facilitates outcome prediction in older trauma patients. *J Am Geriat Soc*. 2012;60(8):1465-1470.

[3] Teasdale G, Jennett B. Assessment of coma and impaired consciousness. A practical scale. *Lancet*. 1974;2(7872):81-84.

[4] Teasdale G, Murray G, Parker L, Jennett B. Adding up the Glasgow coma score. In: Brihaye J, Clarke PRR, Loew F, et al., eds. *Proceedings of the 6th European Congress of Neurosurgery*. Vienna: Springer; 1979:13-16.

[5] Marbac M, Sedki M. VarSelLCM: An R/C++ package for variable selection in model-based clustering of mixed-data with missing values. *Bioinformatics.* 2019; 35: 1255–1257.

[6] Wilkerson MDea. ConsensusClusterPlus: a class discovery tool with confidence assessments and item tracking. Bioinformatics. 2010;26(12):1572-1573.

[7] Masuda T, Tomita M, Ishihama Y. Phase transfer surfactant-aided trypsin digestion for membrane proteome analysis. *J Proteome Res*. 2008;7(2):731-740.

Supplementary Tables

Supplemental Table 1. Silhouette plot clustering of patient characteristics in the derivation cohort

Clinical Phenotype	D-1	D-2	D-3	D-4	D-5	D-6	D-7	D-8	Overall
Number of patients	3,914	1,629	2,879	8,056	7,476	3,108	8,857	2,178	38,097
Age, years, median [IQR]	43 [25–62]	44 [24–66]	81 [74–87]	40 [22–57]	78 [65–85]	49 [27–67]	65 [48–76]	66 [46–77]	62 [39–77]
Male gender, no. (%)	2,683 (68.5)	1,178 (72.3)	1,170 (40.6)	6,660 (82.7)	2,362 (31.6)	2,314 (74.5)	6,475 (73.1)	1,502 (69.0)	24,344 (63.9)
CPS, median [IQR]	1 [1–1]	1 [1–1]	3 [3–4]	1 [1–1]	1 [1–2]	1 [1–1]	1 [1–1]	1 [1–1]	1 [1–1]
Respiratory rate, median [IQR]	22 [19–28]	20 [18–24]	20 [17–23]	22 [18–26]	19 [16–22]	20 [17–24]	19 [16–22]	20 [16–25]	20 [17–24]
Heart rate, median [IQR]	88 [75–104]	84 [73–98]	81 [71–93]	86 [75–99]	79 [70–90]	85 [74–98]	79 [68–90]	92 [76–113]	82 [72–96]
Systolic blood pressure, median [IQR]	120 [100–138]	132 [116–151]	150 [130–170]	130 [116–147]	148 [128–167]	135 [119–154]	142 [124–164]	138 [109–167]	138 [119–158]
Body temperature, median [IQR]	36.4 [35.8–36.8]	36.5 [36.0–36.9]	36.6 [36.1–37.0]	36.6 [36.1–37.0]	36.6 [36.2–37.0]	36.4 [35.9–36.8]	36.4 [35.9–36.8]	36.0 [35.3–36.5]	36.5 [36.0–36.9]
Glasgow Coma Scale, median [IQR]	15 [14–15]	15 [13–15]	15 [14–15]	15 [14–15]	15 [15–15]	14 [13–15]	15 [14–15]	3 [3–6]	15 [14–15]
Head & Cervical AIS, median [IQR]	0 [0–0]	2 [0–3]	0 [0–3]	0 [0–1]	0 [0–0]	3 [0–4]	4 [3–4]	5 [4–5]	1 [0–3]
Face AIS, median [IQR]	0 [0–0]	0 [0–0]	0 [0–0]	0 [0–0]	0 [0–0]	2 [2–2]	0 [0–0]	0 [0–0]	0 [0-0]
Chest AIS, median [IQR]	2 [0–3]	0 [0–3]	0 [0–0]	3 [0–3]	0 [0–0]	0 [0–2]	0 [0–0]	0 [0–3]	0 [0-2]
Abdomen AIS, median [IQR]	3 [2–3]	0 [0–0]	0 [0–0]	0 [0–0]	0 [0–0]	0 [0–0]	0 [0–0]	0 [0–0]	0 [0–0]
Extremities AIS, median [IQR]	1 [0–2]	1 [0–2]	3 [0–3]	2 [0–3]	3 [3–3]	1 [0–2]	0 [0–0]	0 [0–2]	2 [0–3]
External AIS, median [IQR]	0 [0–0]	1 [1–1]	0 [0–0]	0 [0–0]	0 [0–0]	0 [0–0]	0 [0–0]	0 [0–0]	0 [0–0]
ISS, median [IQR]	18 [12–29]	14 [9–22]	9 [9–16]	10 [9–18]	9 [9–9]	17 [13–24]	16 [9–17]	25 [21–34]	13 [9–20]
RTS, median [IQR]	7.84 [7.55–7.84]	7.84 [7.55–7.84]	7.84 [7.84–7.84]	7.84 [7.84–7.84]	7.84 [7.84–7.84]	7.84 [7.11–7.84]	7.84 [7.84–7.84]	4.09 [4.09–5.21]	7.84 [7.84–7.84]
TRISS Ps, median [IQR]	0.97 [0.90–0.99]	0.98 [0.94–0.99]	0.97 [0.94–0.97]	0.99 [0.96–0.99]	0.97 [0.97–0.97]	0.96 [0.91–0.99]	0.96 [0.94–0.98]	0.45 [0.26–0.67]	0.97 [0.94–0.99]
Survival, no. (%)	3,701 (94.6)	1,557 (95.6)	2,734 (95.0)	7,960 (98.8)	7,356 (98.4)	3,011 (96.9)	8,591 (97.0)	1,119 (1059/51.4)	36,029 (94.6)

Abbreviations: IQR: interquartile range; CPS: Charlson polypharmacy scale; AIS: Abbreviated Injury Scale; ISS: Injury Severity Scale; RTS: Revised Trauma Score; TRISS Ps; Trauma and Injury Severity Score

Probability of survival

Supplemental Table 2. Characteristics of patients in high-mortality cluster in the derivation cohort

Clinical Phenotype	D-8α	D-8β	D-8γ	D-8δ	Overall
Number of patients	464	178	957	579	2,178
Age, years, median [IQR]	22 [17–32]	71 [56–80]	72 [64–81]	67 [58–77]	66 [46–77]
Male gender, no. (%)	345 (74.2)	126 (70.8)	654 (68.3)	377 (65.2)	1502 (69.0)
CPS, median [IQR]	1 [1–1]	1 [1–1]	1 [1–1]	1 [1–1]	1 [1–1]
Respiratory rate, median [IQR]	21 [16–27]	20 [15–26]	20 [16–24]	21 [16–26]	20 [16–25]
Heart rate, median [IQR]	101 [80–126]	84 [62–106]	89 [75–106]	94 [79–114]	92 [76–113]
Systolic blood pressure, median [IQR]	130 [112–151]	99 [73–129]	161 [138–191]	118 [86–143]	138 [109–167]
Body temperature, median [IQR]	36.2 [35.7–36.8]	32.5 [29.9–33.8]	36.0 [35.5–36.5]	36.0 [35.4–36.4]	36.0 [35.3–36.5]
Glasgow Coma Scale, median [IQR]	4 [3–6]	3 [3–6]	4 [3–5]	3 [3–6]	3 [3–6]
Head & Cervical AIS, median [IQR]	5 [4–5]	4 [3–5]	5 [4–5]	5 [3–5]	5 [4–5]
Face AIS, median [IQR]	0 [0–0]	0 [0–0]	0 [0–0]	0 [0–0]	0 [0–0]
Chest AIS, median [IQR]	0 [0–3]	0 [0–3]	0 [0–0]	3 [2–4]	0 [0–3]
Abdomen AIS, median [IQR]	0 [0–0]	0 [0–0]	0 [0–0]	0 [0–2]	0 [0–0]
Extremities AIS, median [IQR]	0 [0–2]	0 [0–2]	0 [0–0]	2 [1–3]	0 [0–2]
External AIS, median [IQR]	0 [0–0]	0 [0–0]	0 [0–0]	0 [0–0]	0 [0–0]
ISS, median [IQR]	26 [20–3]	25 [16–30]	25 [17–26]	35 [26–43]	25 [21–34]
RTS, median [IQR]	4.21 [4.09–5.68]	4.09 [3.36–5.23]	4.09 [4.09–5.03]	4.09 [3.57–5.35]	4.09 [4.09–5.21]
TRISS Ps, median [IQR]	0.72 [0.51–0.87]	0.44 [0.20–0.70]	0.45 [0.27–0.63]	0.23 [0.09–0.43]	0.45 [0.26–0.67]
Survival, no. (%)	329 (70.8)	95 (53.4)	415 (43.4)	280 (48.4)	1,119 (51.4)

Abbreviations: IQR: interquartile range; CPS: Charlson polypharmacy scale; AIS: Abbreviated Injury Scale; ISS: Injury Severity Scale; RTS: Revised Trauma Score; TRISS Ps; Trauma and Injury Severity Score Probability of survival

Supplemental Table 3. Silhouette plot clustering of patient characteristics in the validation cohort

Clinical Phenotype	V-1	V-2	V-3	V-4	V-5	V-6	V-7	V-8	Overall
Number of patients	3,242	5,356	1,516	2,376	4,033	5,674	1,872	1,241	25,310
Age, years, median [IQR]	61 [46–74]	82 [73–88]	49 [27–68]	50 [32–67]	28 [17–45]	72 [62–81]	53 [31–70]	67 [47–79]	65 [43–79]
Male gender, no. (%)	2,361 (72.8)	1,247 (23.3)	1,106 (73.0)	1,632 (68.7)	3,397 (84.2)	3,998 (70.5)	1,399 (74.7)	856 (69.0)	15,996 (63.2)
CPS, median [IQR]	1 [1–1]	2 [1–2]	1 [1–1]	1 [1–1]	1 [1–1]	2 [1–2]	1 [1–1]	1 [1–1]	1 [1–2]
Respiratory rate, median [IQR]	22 [18–26]	19 [16–21]	20 [18–24]	22 [18–28]	20 [17–24]	18 [16–22]	20 [17–24]	20 [16–25]	20 [17–24]
Heart rate, median [IQR]	82 [72–95]	80 [70–91]	84 [73–97]	87 [75–102]	85 [75–98]	79 [68–90]	84 [72–97]	98 [80–118]	82 [72–95]
Systolic blood pressure, median [IQR]	138 [120–157]	151 [131–170]	134 [120–154]	122 [104–140]	128 [114–142]	149 [129–170]	137 [119–159]	141 [111–169]	139 [120–160]
Body temperature, median [IQR]	36.5 [36.0–36.8]	36.7 [36.3–37.0]	36.5 [36.1–37.0]	36.4 [36.0–36.8]	36.8 [36.4–37.2]	36.4 [36.0–36.8]	36.4 [36.0–36.8]	36.1 [35.5–36.6]	36.5 [36.1–36.9]
Glasgow Coma Scale, median [IQR]	15 [14–15]	15 [15–15]	15 [14–15]	15 [14–15]	15 [15–15]	15 [14–15]	14 [13–15]	3 [3–5]	15 [14–15]
Head & Cervical AIS, median [IQR]	0 [0–2]	0 [0–0]	2 [0–3]	0 [0–1]	0 [0–2]	4 [3–4]	3 [1–4]	5 [4–5]	1 [0–4]
Face AIS, median [IQR]	0 [0–0]	0 [0–0]	0 [0–0]	0 [0–0]	0 [0–0]	0 [0–0]	2 [2–2]	0 [0–0]	0 [0–0]
Chest AIS, median [IQR]	3 [3–4]	0 [0–0]	0 [0–3]	3 [0–3]	0 [0–0]	0 [0–0]	0 [0–3]	0 [0–3]	0 [0–2]
Abdomen AIS, median [IQR]	0 [0–0]	0 [0–0]	0 [0–0]	3 [2–3]	0 [0–0]	0 [0–0]	0 [0–0]	0 [0–0]	0 [0–0]
Extremities AIS, median [IQR]	1 [0–2]	3 [3–3]	1 [0–2]	1 [0–2]	2 [2–3]	0 [0–0]	1 [0–2]	0 [0–2]	2 [0–3]
External AIS, median [IQR]	0 [0–0]	0 [0–0]	1 [1–1]	0 [0–0]	0 [0–0]	0 [0–0]	0 [0–0]	0 [0–0]	0 [0–0]
ISS, median [IQR]	16 [10–24]	9 [9–9]	14 [9–21]	18 [13–29]	9 [5–10]	16 [10–17]	20 [13–24]	26 [25–36]	13 [9–20]
RTS, median [IQR]	7.84 [7.84–7.84]	7.84 [7.84–7.84]	7.84 [7.55–7.84]	7.84 [7.55–7.84]	7.84 [7.84–7.84]	7.84 [7.84–7.84]	7.84 [7.11–7.84]	4.09 [4.09–5.03]	7.84 [7.84–7.84]
TRISS Ps, median [IQR]	0.96 [0.92–0.98]	0.97 [0.97–0.97]	0.98 [0.94–0.99]	0.96 [0.89–0.99]	0.99 [0.99–1.00]	0.94 [0.93–0.97]	0.96 [0.89–0.99]	0.40 [0.21–0.63]	0.97 [0.94–0.99]
Survival, no. (%)	3,168 (97.7)	5,264 (98.3)	1,466 (96.7)	2,259 (95.1)	4,013 (99.5)	5,475 (96.5)	1,820 (97.2)	647 (52.1)	24,112 (95.3)

Abbreviations: IQR: interquartile range; CPS: Charlson polypharmacy scale; AIS: Abbreviated Injury Scale; ISS: Injury Severity Scale; RTS: Revised Trauma Score; TRISS Ps; Trauma and Injury Severity Score Probability of survival

Supplemental Table 4. Characteristics of patients in high-mortality cluster in the validation cohort

Clinical Phenotype	V-8α	V-8β	V-8γ	V-8δ	Overall
Number of patients	104	281	372	484	1,241
Age, years, median [IQR]	75 [66–84]	64 [43–78]	42 [25–53]	76 [68–83]	67 [47–79]
Male gender, no. (%)	78 (75.0)	155 (55.2)	317 (85.2)	306 (63.2)	856 (69.0)
CPS, median [IQR]	1 [1–2]	1 [1–1]	1 [1–1]	1 [1–2]	1 [1–1]
Respiratory rate, median [IQR]	18 [12–25]	20 [14–24]	22 [18–27]	20 [17–24]	20 [16–25]
Heart rate, median [IQR]	85 [64–104]	111 [91–129]	100 [82–120]	92 [77–108]	98 [80–118]
Systolic blood pressure, median [IQR]	125 [100–156]	94 [69–121]	145 [126–163]	164 [140–187]	141 [111–169]
Body temperature, median [IQR]	33.6 [30.7–35.3]	35.6 [35.0–36.2]	36.4 [36.0–36.8]	36.2 [35.8–36.6]	36.1 [35.5–36.6]
Glasgow Coma Scale, median [IQR]	3 [3–6]	3 [3–4]	4 [3–6]	3 [3–5]	3 [3–5]
Head & Cervical AIS, median [IQR]	5 [4–5]	5 [4–5]	5 [4–5]	5 [4–5]	5 [4–5]
Face AIS, median [IQR]	0 [0–0]	0 [0–1]	0 [0–0]	0 [0–0]	0 [0–0]
Chest AIS, median [IQR]	0 [0–0]	4 [3–4]	0 [0–3]	0 [0–0]	0 [0–3]
Abdomen AIS, median [IQR]	0 [0–0]	0 [0–2]	0 [0–0]	0 [0–0]	0 [0–0]
Extremities AIS, median [IQR]	0 [0–0]	2 [2–3]	0 [0–2]	0 [0–0]	0 [0–2]
External AIS, median [IQR]	0 [0–0]	0 [0–0]	0 [0–0]	0 [0–0]	0.00 [0–0]
ISS, median [IQR]	25 [16–26]	41 [34–45]	26 [25–36]	25 [18–26]	26 [25–36]
RTS, median [IQR]	4.09 [3.57–5.08]	4.09 [2.63–4.74]	4.74 [4.09–5.97]	4.09 [4.09–5.03]	4.09 [4.09–5.03]
TRISS Ps, median [IQR]	0.31 [0.25–0.55]	0.13 [0.04–0.28]	0.68 [0.43–0.82]	0.40 [0.27–0.45]	0.40 [0.21–0.63]
Survival, no. (%)	47 (45.2)	130 (46.3)	258 (69.4)	212 (43.8)	647 (52.1)

Abbreviations: IQR: interquartile range; CPS: Charlson polypharmacy scale; AIS: Abbreviated Injury Scale; ISS: Injury Severity Scale; RTS: Revised Trauma Score; TRISS Ps; Trauma and Injury Severity Score Probability of survival

Supplemental Table 5. Patient characteristics in the cohort for biological profile

Clinical Phenotype	B-1	B-2	B-3	B-4	B-5	B-6	B-7	B-8	Overall
Number of patients	14	14	5	13	13	10	12	9	90
Age, years, median [IQR]	50 [34–69]	40 [34–53]	72 [69–76]	52 [43–74]	50 [47–71]	39 [22–46]	74 [67–84]	66 [52–79]	52 [39–72]
Male gender, no. (%)	9 (64.3)	12 (85.7)	3 (60.0)	8 (61.5)	8 (61.5)	5 (50.0)	8 (66.7)	9 (100.0)	62 (68.9)
CPS, median [IQR]	0 [0–1]	0 [0–1]	4 [4–6]	0 [0–1]	0 [0–0]	0 [0–0]	0 [0–1]	1 [0–1]	0.00 [0–1]
Respiratory rate, median [IQR]	23 [20–25]	21 [18–24]	20 [18–22]	24 [20–29]	20 [18–22]	21 [20–23]	23 [21–27]	21 [17–27]	22 [18–25]
Heart rate, median [IQR]	93 [73–99]	78 [68–88]	82 [80–85]	98 [78–107]	88 [68–108]	89 [77–103]	82 [62–95]	108 [98–126]	89 [72–107]
Systolic blood pressure, median [IQR]	133 [109–152]	137 [122–156]	163 [126–179]	141 [129–159]	134 [125–160]	156 [141–167]	152 [131–164]	169 [152–205]	146 [125–164]
Body temperature, median [IQR]	36.5 [36.3–36.7]	36.6 [36.4–36.7]	36.2 [36.1–36.2]	36.6 [35.7–37.1]	36.5 [36.1–36.8]	36.6 [36.3–36.9]	36.1 [36.0–36.5]	35.8 [35.1–36.4]	36.4 [36.0–36.8]
Glasgow coma scale, median [IQR]	15 [15–15]	15 [14–15]	14 [14–15]	15 [14–15]	15 [14–15]	13 [11–14]	14 [13–14]	3 [3–3]	14 [13–15]
Head & Cervical AIS, median [IQR]	0 [0–0]	0 [0–4]	0 [0–0]	0 [0–3]	0 [0–0]	3 [0–5]	4 [4–5]	5 [5–5]	0 [0–4]
Face AIS, median [IQR]	0 [0–0]	0 [0–0]	0 [0–0]	0 [0–0]	0 [0–0]	2 [2–2]	0 [0–0]	0 [0–0]	0 [0–0]
Chest AIS, median [IQR]	1 [0–2]	0 [0–2]	0 [0–0]	3 [3–3]	0 [0–0]	1 [0–3]	0 [0–0]	0 [0–0]	0 [0–2]
Abdomen AIS, median [IQR]	3 [2–3]	0 [0–0]	0 [0–0]	0 [0–0]	0 [0–0]	0 [0–0]	0 [0–0]	0 [0–0]	0 [0–0]
Extremities AIS, median [IQR]	0 [0–1]	1 [0–2]	1 [1–4]	1 [0–2]	2 [1–3]	0 [0–2]	0 [0–1]	1 [0–2]	1 [0–2]
External AIS, median [IQR]	0 [0–0]	1 [1–1]	0 [0–0]	0 [0–0]	0 [0–0]	0 [0–0]	0 [0–0]	0 [0–0]	0 [0–0]
ISS, median [IQR]	10 [9–18]	8 [2–26]	10 [4–16]	17 [9–22]	4 [1–9]	14 [8–36]	18 [14–25]	29 [25–30]	12 [7–25]
RTS, median [IQR]	7.84 [7.55–7.84]	7.84 [7.84–7.84]	7.84 [7.84–7.84]	7.84 [7.55–7.84]	7.84 [7.84–7.84]	7.84 [7.14–7.84]	7.84 [7.84–7.84]	4.09 [4.09–4.09]	7.84 [7.22–7.84]
TRISS Ps, median [IQR]	0.98 [0.95–0.99]	0.99 [0.94–1.00]	0.96 [0.94–0.98]	0.98 [0.97–0.99]	0.99 [0.98–0.99]	0.99 [0.95–0.99]	0.94 [0.88–0.95]	0.35 [0.21–0.39]	0.97 [0.86–0.99]
Survival, no. (%)	14 (100)	14 (100)	5 (100)	13 (100)	13 (100)	10 (100)	12 (100)	5 (55.6)	86 (95.6)

Supplemental Table 5. Patient characteristics in the cohort for biological profile (continued)

Clinical Phenotype	B-1	B-2	B-3	B-4	B-5	B-6	B-7	B-8	Overall
Sodium, mEq/L, median [IQR]	140 [139–142]	139 [137–140]	142 [138–142]	138 [137–139]	141 [139–142]	139 [139–141]	140 [139–141]	141 [139–142]	140 [138–141]
Potassium, mEq/L, median [IQR]	3.8 [3.6–3.9]	3.8 [3.5–4.2]	3.9 [3.5–4.0]	3.8 [3.5–4.0]	3.8 [3.4–4.1]	3.7 [3.4–4.0]	3.9 [3.8–4.1]	3.2 [3.2–3.6]	3.8 [3.5–4.0]
Chloride, mEq/L, median [IQR]	106 [104–107]	106 [104–108]	105 [103–106]	104 [103–105]	106 [102–107]	104 [103–104]	108 [105–108]	105 [102–106]	105 [103–107]
Total bilirubin, mg/dL, median [IQR]	0.7 [0.5–0.9]	0.4 [0.4–0.5]	0.6 [0.5–1.1]	0.5 [0.4–1.0]	0.6 [0.5–0.8]	0.5 [0.4–0.6]	0.7 [0.5–0.8]	0.7 [0.5–1.1]	0.5 [0.4–0.9]
AST, U/L, median [IQR]	55 [34–99]	48 [28–84]	23 [14–38]	44 [26–59]	30 [20–44]	53 [28–97]	24 [21–27]	39 [31–60]	39 [24–70]
ALT, U/L, median [IQR]	46 [29–71]	35 [27–54]	25 [10–40]	31 [22–46]	26 [17–36]	32 [22–60]	17 [13–22]	20 [12–30]	28 [17–45]
LDH, U/L, median [IQR]	495 [382–560]	331 [247–597]	242 [208–350]	306 [246–396]	255 [195–295]	410 [240–516]	249 [227–278]	413 [310–468]	309 [238–450]
BUN, mg/dL, median [IQR]	16 [11–19]	17 [14–19]	13 [11–13]	17 [13–19]	20 [15–22]	15 [13–15]	16 [12–20]	18 [13–18]	16 [13–19]
Creatinine, mg/dL, median [IQR]	0.79 [0.74–0.96]	0.78 [0.58–0.91]	0.62 [0.62–0.64]	0.79 [0.70–0.89]	0.90 [0.75–1.05]	0.73 [0.57–0.85]	0.82 [0.67–0.93]	0.83 [0.69–0.92]	0.80 [0.66–0.93]
C-Reactive Protein, mg/L, median [IQR]	0.14 [0.09–0.69]	0.07 [0.05–0.14]	0.09 [0.07–0.27]	0.15 [0.12–0.24]	0.22 [0.18–0.22]	0.07 [0.07–0.08]	0.17 [0.07–0.69]	0.12 [0.05–0.82]	0.12 [0.06–0.39]
WBC count, ×10^3/μL, median [IQR]	11.30 [8.08–12.58]	8.49 [7.25–11.61]	8.85 [8.34–8.96]	9.46 [8.67–10.48]	9.03 [7.52–10.65]	13.11 [7.82–16.84]	7.66 [6.72–10.25]	12.43 [8.97–16.94]	9.34 [7.62–12.42]
Hemoglobin, g/dL, median [IQR]	13.4 [12.3–15.3]	13.4 [12.3–14.3]	12.6 [11.7–13.4]	13.4 [11.0–14.2]	13.6 [12.0–14.9]	13.6 [13.3–14.9]	12.8 [11.8–13.3]	13.3 [11.5–13.6]	13.3 [12.0–14.4]
Platelet count ×10^3/μL, median [IQR]	191 [157–242]	220 [191–261]	157 [149–188]	220 [209–292]	206 [190–274]	265 [220–290]	199 [181–212]	200 [145–254]	209 [178–261]
PT-INR, median [IQR]	1.10 [0.96–1.28]	1.04 [0.97–1.10]	0.95 [0.94–1.19]	1.07 [1.03–1.09]	1.00 [0.98–1.05]	1.01 [0.96–1.07]	1.05 [1.00–1.11]	1.25 [1.11–1.40]	1.05 [0.98–1.13]
APTT, sec, median [IQR]	26 [23–29]	29 [26–31]	25 [23–35]	27 [26–28]	27 [24–28]	26 [25–27]	27 [24–30]	38 [28–40]	27 [24–29]
FDP, μg/mL, median [IQR]	46.8 [30.1–112.6]	17.6 [5.7–52.4]	5.4 [2.8–28.2]	28.2 [14.4–80.0]	9.8 [3.4–30.3]	10.5 [6.0–32.1]	20.8 [11.4–39.0]	193 [138.5–361.3]	28.2 [6.5–80.0]
D-dimer, μg/mL, median [IQR]	24.1 [11.5–47.4]	6.5 [1.4–18.2]	2.9 [1.2–18.5]	16.9 [4.1–34.4]	3.0 [0.8–11.6]	3.6 [1.8–11.6]	8.5 [3.7–15.0]	39.1 [28.3–99.9]	11.03 [2.87–28.1]
Lactate, mmol/L, median [IQR]	2.1 [1.7–3.5]	1.8 [1.5–2.7]	2.0 [1.6–2.2]	1.9 [1.2–2.4]	1.9 [1.2–2.2]	2.0 [1.6–3.1]	1.8 [1,4–2.0]	2.6 [2.3–4.0]	2.0 [1.4–2.9]
Proteomics sample collection time from injury, hour [IQR]	1.0 [1.0–1.2]	1.0 [0.9–1.5]	1.2 [1.0–1.5]	1.2 [1.0–1.5]	1.0 [1.0–3.0]	0.9 [0.8–1.2]	0.9 [0.8–1.1]	2.0 [0.8–3.5]	1.0 [0.8–1.7]

Abbreviations: IQR: interquartile range; CPS: Charlson polypharmacy scale; AIS: Abbreviated Injury Scale; ISS: Injury Severity Scale; RTS: Revised Trauma Score; TRISS Ps; Trauma and Injury Severity Score Probability of survival; AST: aspartate transaminase; ALT: alanine transaminase; LDH: lactate dehydrogenase; BUN: blood urea nitrogen; WBC: white blood cell; PT-INR: prothrombin time-international normalized ratio; APTT: activated partial thromboplastin time; FDP: fibrinogen/fibrin degradation products

Supplemental Table 6. Significantly regulated plasma proteins identified using limma voom algorithm

Gene symbol	Protein Accession	Protein names	Length	Change	fold change	Log2 (fold change)	Adjusted p value	Adjusted p value (-log10)	Manhattan distance
SAA1	P0DJI8	Serum amyloid A-1 protein (SAA) [Cleaved into: Amyloid protein A (Amyloid fibril protein AA); Serum amyloid protein A (2-104); Serum amyloid protein A (3-104); Serum amyloid protein A (2-103); Serum amyloid protein A(2-102); Serum amyloid protein A(4-101)]	122	Up-regulated	2.267	1.181	0.116	0.936	2.116
SERPINA3	P01011	Alpha-1-antichymotrypsin (ACT) (Cell growth-inhibiting gene 24/25 protein) (Serpin A3) [Cleaved into: Alpha-1-antichymotrypsin His-Pro-less]	423	Up-regulated	1.564	0.646	0.116	0.936	1.581
LRG1	P02750	Leucine-rich alpha-2-glycoprotein (LRG)	347	Up-regulated	1.448	0.534	0.116	0.936	1.470
SAA2	P0DJI9	Serum amyloid A-2 protein (SAA2) [Cleaved into: Amyloid A2 protein (AA2)]	122	Up-regulated	1.642	0.716	0.180	0.745	1.460
LBP	P18428	Lipopolysaccharide-binding protein (LBP)	481	Up-regulated	1.430	0.516	0.116	0.936	1.452
B2M	P61769	Beta-2-microglobulin [Cleaved into: Beta-2-microglobulin form pI 5.3]	119	Up-regulated	1.437	0.523	0.118	0.928	1.451
C9	P02748	Complement component C9 [Cleaved into: Complement component C9a; Complement component C9b]	559	Up-regulated	1.309	0.388	0.138	0.860	1.248
CFHR1	Q03591	Complement factor H-related protein 1 (FHR-1) (H factor-like protein 1) (H-factor-like 1) (H36)	330	Up-regulated	1.221	0.288	0.116	0.936	1.224
C2	P06681	Complement C2 (EC 3.4.21.43) (C3/C5 convertase) [Cleaved into: Complement C2b fragment; Complement C2a fragment]	752	Up-regulated	1.264	0.338	0.144	0.842	1.179
CFHR2	P36980	Complement factor H-related protein 2 (FHR-2) (DDESK59) (H factor-like 3) (H factor-like protein 2)	270	Up-regulated	1.325	0.406	0.175	0.757	1.163

LGALS3BP	Q08380	Galectin-3-binding protein (Basement membrane autoantigen p105) (Lectin galactoside-binding soluble 3-binding protein) (Mac-2-binding protein) (MAC2BP) (Mac-2 BP) (Tumor-associated antigen 90K)	585	Up-regulated	1.288	0.366	0.181	0.742	1.108
HBB	P68871	Hemoglobin subunit beta (Beta-globin) (Hemoglobin beta chain) [Cleaved into: LVV-hemorphin-7; Spinorphin]	147	Down-regulated	0.499	-1.004	0.118	0.928	1.932
FN1	P02751	Fibronectin (FN) (Cold-insoluble globulin) (CIG) [Cleaved into: Anastellin; Ugl-Y1; Ugl-Y2; Ugl-Y3]	2477	Down-regulated	0.535	-0.901	0.105	0.979	1.880
HBD	P02042	Hemoglobin subunit delta (Delta-globin) (Hemoglobin delta chain)	147	Down-regulated	0.544	-0.878	0.184	0.735	1.613
GSN	P06396	Gelsolin (AGEL) (Actin-depolymerizing factor) (ADF) (Brevin)	782	Down-regulated	0.643	-0.638	0.116	0.936	1.573
PPBP	P02775	Platelet basic protein (PBP) (C-X-C motif chemokine 7) (Leukocyte-derived growth factor) (LDGF) (Macrophage-derived growth factor) (MDGF) (Small-inducible cytokine B7) [Cleaved into: Connective tissue-activating peptide III (CTAP-III) (LA-PF4) (Low-affinity platelet factor IV); TC-2; Connective tissue-activating peptide III(1-81) (CTAP-III(1-81)); Beta-thromboglobulin (Beta-TG); Neutrophil-activating peptide 2(74) (NAP-2(74)); Neutrophil-activating peptide 2(73) (NAP-2(73)); Neutrophil-activating peptide 2 (NAP-2); TC-1; Neutrophil-activating peptide 2(1-66) (NAP-2(1-66)); Neutrophil-activating peptide 2(1-63) (NAP-2(1-63))]	128	Down-regulated	0.678	-0.560	0.116	0.936	1.496
SERPIND1	P05546	Heparin cofactor 2 (Heparin cofactor II) (HC-II) (Protease inhibitor leuserpin-2) (HLS2) (Serpin D1)	499	Down-regulated	0.687	-0.542	0.116	0.936	1.478
HP	P00738	Haptoglobin (Zonulin) [Cleaved into: Haptoglobin alpha chain; Haptoglobin beta chain]	406	Down-regulated	0.628	-0.670	0.160	0.796	1.466
PON1	P27169	Serum paraoxonase/arylesterase 1 (PON 1) (EC 3.1.1.2) (EC 3.1.1.81) (EC 3.1.8.1) (Aromatic esterase 1) (A-esterase 1) (K-45) (Serum aryldialkylphosphatase 1)	355	Down-regulated	0.698	-0.518	0.116	0.936	1.453

APOC2	P02655	Apolipoprotein C-II (Apo-CII) (ApoC-II) (Apolipoprotein C2) [Cleaved into: Proapolipoprotein C-II (ProapoC-II)]	101	Down-regulated	0.699	-0.516	0.116	0.936	1.451
APOC1	P02654	Apolipoprotein C-I (Apo-CI) (ApoC-I) (Apolipoprotein C1) [Cleaved into: Truncated apolipoprotein C-I]	83	Down-regulated	0.675	-0.567	0.135	0.870	1.436
CLEC3B	P05452	Tetranectin (TN) (C-type lectin domain family 3 member B) (Plasminogen kringle 4-binding protein)	202	Down-regulated	0.708	-0.499	0.116	0.936	1.434
HBG1	P69891	Hemoglobin subunit gamma-1 (Gamma-1-globin) (Hb F Agamma) (Hemoglobin gamma-1 chain) (Hemoglobin gamma-A chain)	147	Down-regulated	0.659	-0.602	0.150	0.824	1.426
APOC3	P02656	Apolipoprotein C-III (Apo-CIII) (ApoC-III) (Apolipoprotein C3)	99	Down-regulated	0.727	-0.460	0.116	0.936	1.396
SERPINA4	P29622	Kallistatin (Kallikrein inhibitor) (Peptidase inhibitor 4) (PI-4) (Serpin A4)	427	Down-regulated	0.783	-0.353	0.116	0.936	1.289
PGLYRP2	Q96PD5	N-acetylmuramoyl-L-alanine amidase (EC 3.5.1.28) (Peptidoglycan recognition protein 2) (Peptidoglycan recognition protein long) (PGRP-L)	576	Down-regulated	0.797	-0.328	0.116	0.936	1.264
F13B	P05160	Coagulation factor XIII B chain (Fibrin-stabilizing factor B subunit) (Protein-glutamine gamma-glutamyltransferase B chain) (Transglutaminase B chain)	661	Down-regulated	0.817	-0.292	0.116	0.936	1.228
APOA1	P02647	Apolipoprotein A-I (Apo-AI) (ApoA-I) (Apolipoprotein A1) [Cleaved into: Proapolipoprotein A-I (ProapoA-I); Truncated apolipoprotein A-I (Apolipoprotein A-I(1-242))]	267	Down-regulated	0.716	-0.482	0.183	0.738	1.219
F2	P00734	Prothrombin (EC 3.4.21.5) (Coagulation factor II) [Cleaved into: Activation peptide fragment 1; Activation peptide fragment 2; Thrombin light chain; Thrombin heavy chain]	622	Down-regulated	0.823	-0.282	0.116	0.936	1.217
APOB	P04114	Apolipoprotein B-100 (Apo B-100) [Cleaved into: Apolipoprotein B-48 (Apo B-48)]	4563	Down-regulated	0.827	-0.274	0.116	0.936	1.210

KLKB1	P03952	Plasma kallikrein (EC 3.4.21.34) (Fletcher factor) (Kininogenin) (Plasma prekallikrein) (PKK) [Cleaved into: Plasma kallikrein heavy chain; Plasma kallikrein light chain]	638	Down-regulated	0.831	-0.266	0.116	0.936	1.202
PZP	P20742	Pregnancy zone protein (C3 and PZP-like alpha-2-macroglobulin domain-containing protein 6)	1482	Down-regulated	0.820	-0.286	0.129	0.889	1.176
IGFALS	P35858	Insulin-like growth factor-binding protein complex acid labile subunit (ALS)	605	Down-regulated	0.795	-0.332	0.144	0.842	1.174
PRDX2	P32119	Peroxiredoxin-2 (EC 1.11.1.24) (Natural killer cell-enhancing factor B) (NKEF-B) (PRP) (Thiol-specific antioxidant protein) (TSA) (Thioredoxin peroxidase 1) (Thioredoxin-dependent peroxide reductase 1) (Thioredoxin-dependent peroxiredoxin 2)	198	Down-regulated	0.762	-0.391	0.176	0.754	1.146
ORM2	P19652	Alpha-1-acid glycoprotein 2 (AGP 2) (Orosomucoid-2) (OMD 2)	201	Down-regulated	0.826	-0.276	0.137	0.863	1.139
TF	P02787	Serotransferrin (Transferrin) (Beta-1 metal-binding globulin) (Siderophilin)	698	Down-regulated	0.816	-0.293	0.164	0.785	1.078
PPIA	P62937	Peptidyl-prolyl cis-trans isomerase A (PPIase A) (EC 5.2.1.8) (Cyclophilin A) (Cyclosporin A-binding protein) (Rotamase A) [Cleaved into: Peptidyl-prolyl cis-trans isomerase A, N-terminally processed]	165	Down-regulated	0.814	-0.297	0.166	0.780	1.076

Protein accession numbers retrieved from UNIPROT (Knowledgebase). All protein names were collected from UniProtKb (SwissProt). Manhattan distance (|ΔX| +|ΔY|) of the data from the origin (0,0). Differentially protein expression was defined as adjusted p value <0.2 and 1.2-fold change.

Supplemental Table 7. GO enrichment analysis results

Go terms for trauma (Up-regulated protein)

ID	Description	p value	Adjusted p value	Protein	Count
GO:0002697	regulation of immune effector process	1.31E-07	1.30E-05	LBP/B2M/C9/CFHR1/C2/CFHR2	6
GO:0019835	cytolysis	1.83E-09	5.47E-07	LBP/C9/CFHR1/CFHR2	4
GO:0006953	acute-phase response	1.37E-08	2.04E-06	SAA1/SERPINA3/SAA2/LBP	4
GO:0030449	regulation of complement activation	5.00E-07	2.99E-05	C9/CFHR1/C2/CFHR2	4
GO:0002920	regulation of humoral immune response	9.29E-07	4.63E-05	C9/CFHR1/C2/CFHR2	4
GO:0001906	cell killing	2.59E-06	9.69E-05	B2M/C9/CFHR1/CFHR2	4
GO:0006959	humoral immune response	5.31E-05	1.59E-03	C9/CFHR1/C2/CFHR2	4
**Go terms for trauma (Down-regulated protein)**					
ID	Description	p value	Adjusted p value	Protein	Count
GO:0007596	blood coagulation	7.45E-10	6.75E-08	HBB/FN1/HBD/SERPIND1/HBG1/F13B/F2/KLKB1/PRDX2	9
GO:0007599	hemostasis	8.46E-10	6.75E-08	HBB/FN1/HBD/SERPIND1/HBG1/F13B/F2/KLKB1/PRDX2	9
GO:0050817	coagulation	8.68E-10	6.75E-08	HBB/FN1/HBD/SERPIND1/HBG1/F13B/F2/KLKB1/PRDX2	9
GO:0002576	platelet degranulation	5.08E-10	6.75E-08	FN1/PPBP/CLEC3B/SERPINA4/APOA1/ORM2/TF	7
GO:0033344	cholesterol efflux	3.61E-10	6.75E-08	PON1/APOC2/APOC1/APOC3/APOA1/APOB	6
GO:0034368	protein-lipid complex remodeling	7.17E-10	6.75E-08	APOC2/APOC1/APOC3/APOA1/APOB	5
GO:0034369	plasma lipoprotein particle remodeling	7.17E-10	6.75E-08	APOC2/APOC1/APOC3/APOA1/APOB	5
GO:0034377	plasma lipoprotein particle assembly	7.17E-10	6.75E-08	APOC2/APOC1/APOC3/APOA1/APOB	5
GO:0034367	protein-containing complex remodeling	8.49E-10	6.75E-08	APOC2/APOC1/APOC3/APOA1/APOB	5
GO:0042744	hydrogen peroxide catabolic process	8.49E-10	6.75E-08	HBB/HBD/HP/HBG1/PRDX2	5
GO:0065005	protein-lipid complex assembly	1.36E-09	8.99E-08	APOC2/APOC1/APOC3/APOA1/APOB	5

Supplementary Figures

Supplemental Figure.1 Flowchart of patient selection

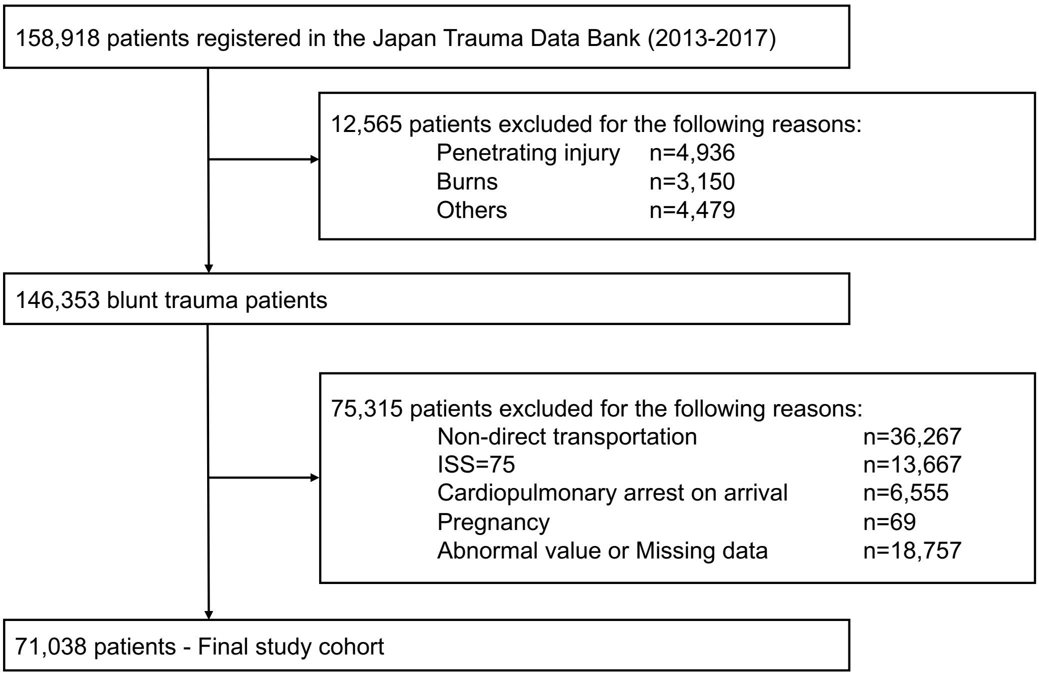

Supplemental Figure.2 Heatmap of correlation between clinical variables for phenotyping

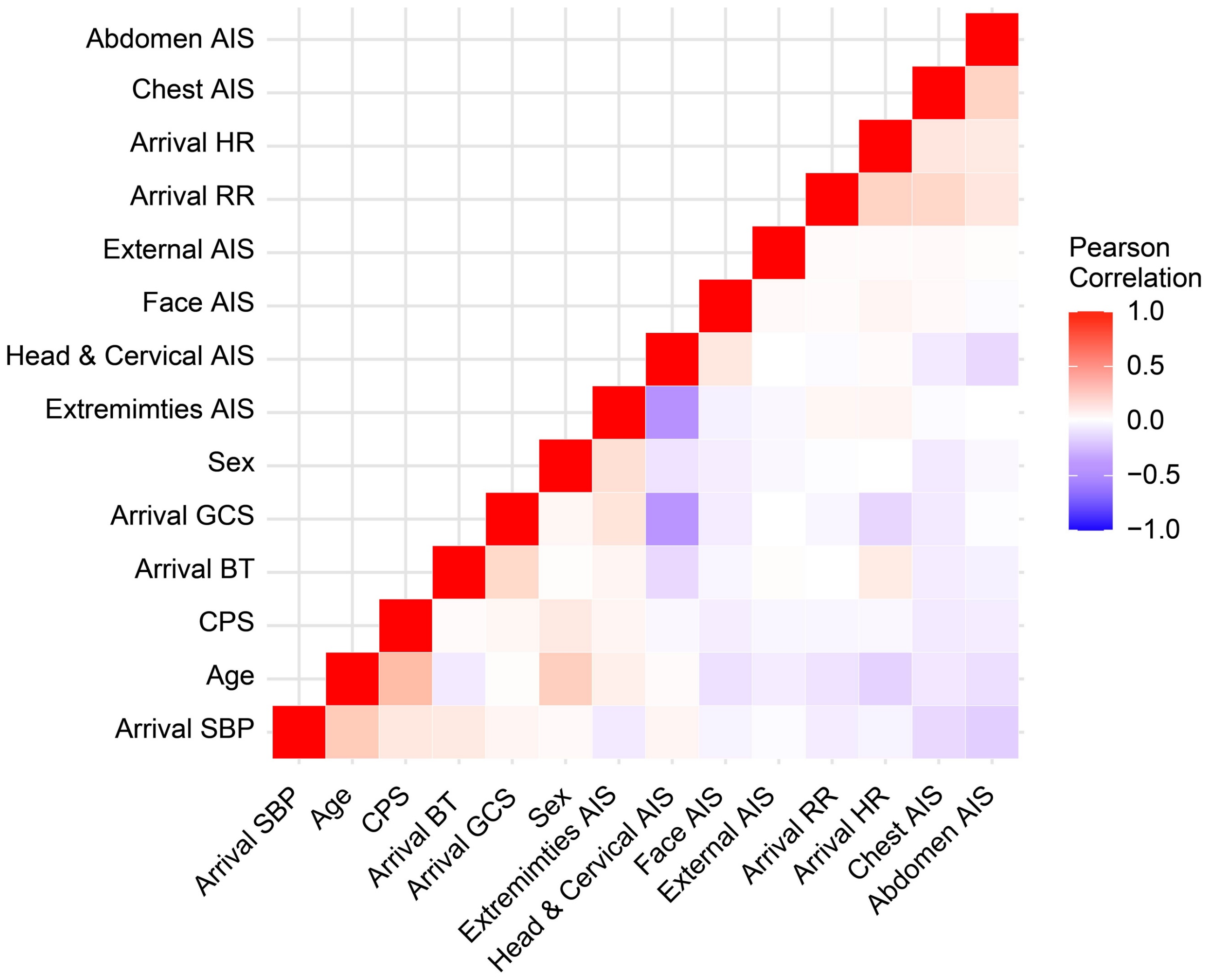

Heatmap showing the correlation among clinical variables for phenotyping. Intense red or blue colors are shown when the Spearman rank order correlation coefficient is higher in positive or negative directions, respectively. The absolute values of the correlation coefficients were evaluated to verify their exceeding 0.5. AIS, Abbreviated Injury Scale; BT, body temperature; GCS, Glasgow Coma Scale; SBP, systolic blood pressure; HR, heart rate; RR, respiratory rate; CPS, Charlson Polypharmacy Scale.

Supplemental Figure.3 Optimal number of clusters in the derivation cohort (average silhouette width)

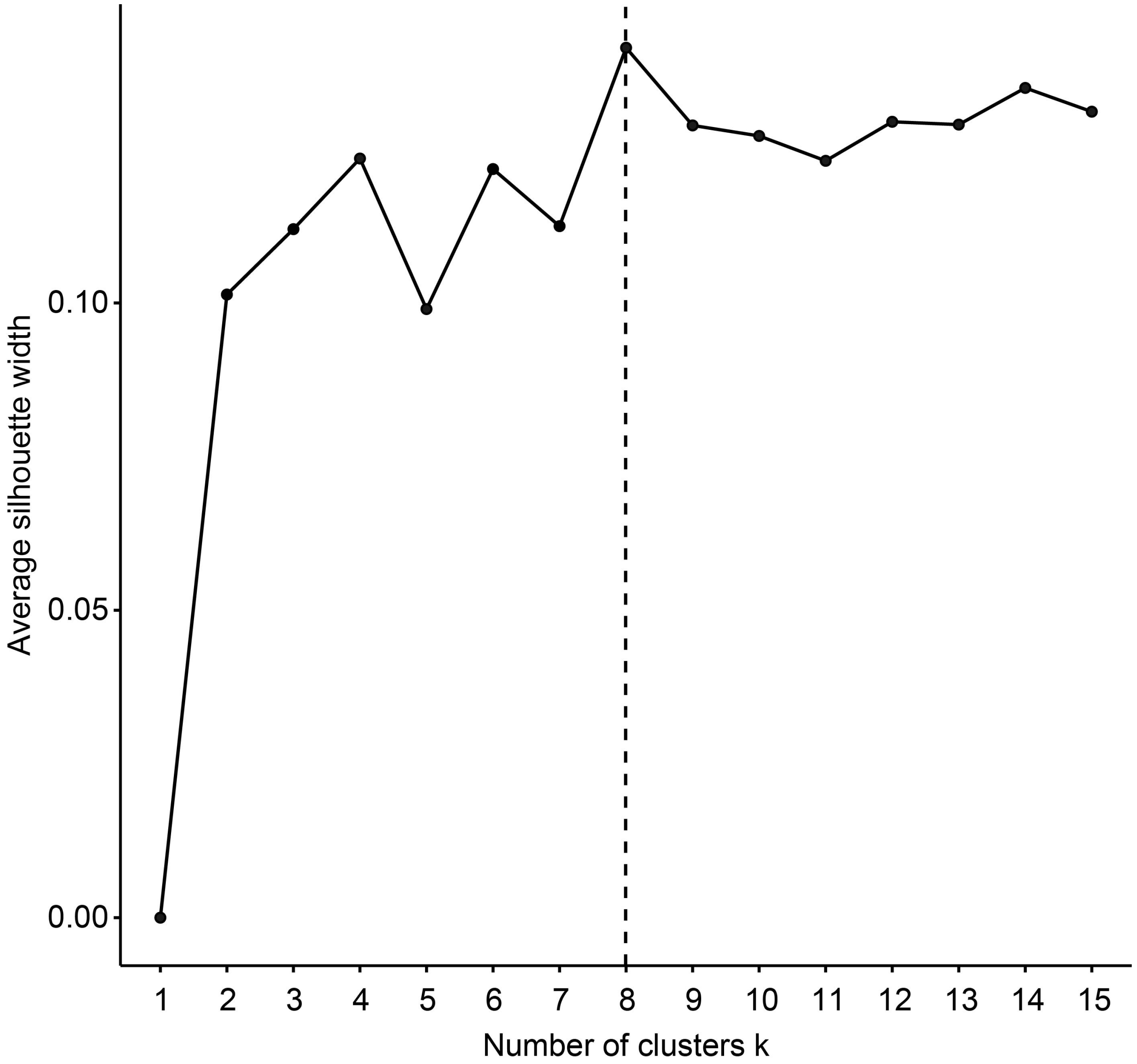

Optimal number of clusters in the derivation cohort (average silhouette width). The silhouette method was used to evaluate how well each individual fit within their cluster and estimate the mean distance between clusters. The silhouette coefficients ranged from −1 to +1, where a high value indicates that individuals are well-matched to their clusters and poorly matched to neighboring clusters. A high average silhouette width indicates a good clustering. Thus, the vertical dashed line indicates that the mean silhouette width as the highest for $k = 8$.

Supplemental Figure.4 Optimal number of clusters in the derivation cohort (elbow plot by k-means)

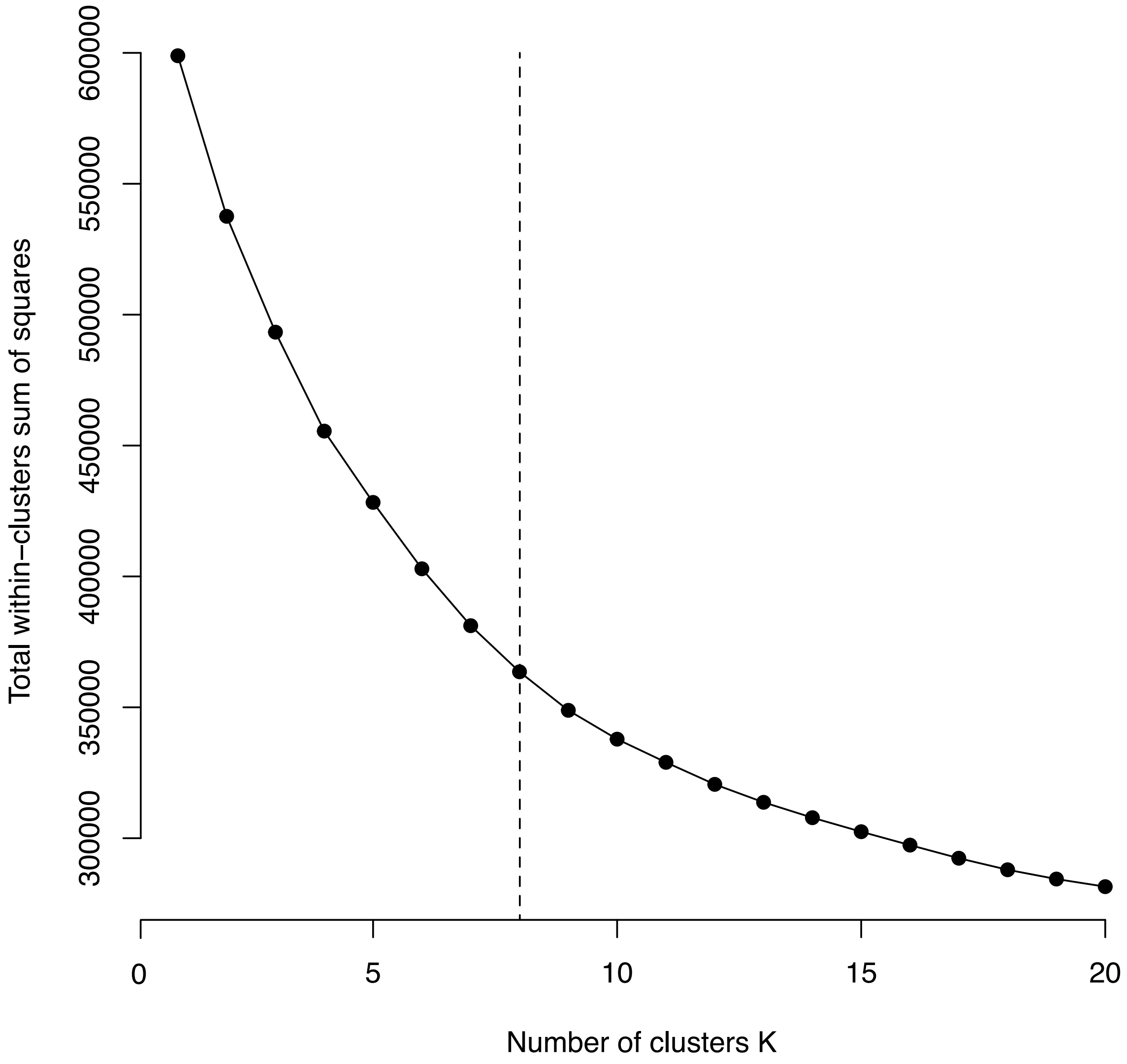

Optimal number of clusters in the derivation cohort (elbow plot according to *k*-means clustering). Clustering of the derivation cohort. The elbow plot was generated by calculating the within-cluster sum of squares (*k* range: 1–20) in the derivation cohort. The location of a bend (elbow) in the plot is generally considered as an indicator of the appropriate number of clusters. The vertical dashed line indicates $k = 8$, with the elbow located near this area.

Supplemental Figure.5 Silhouette plot in the derivation cohort (excluding the negative silhouette)

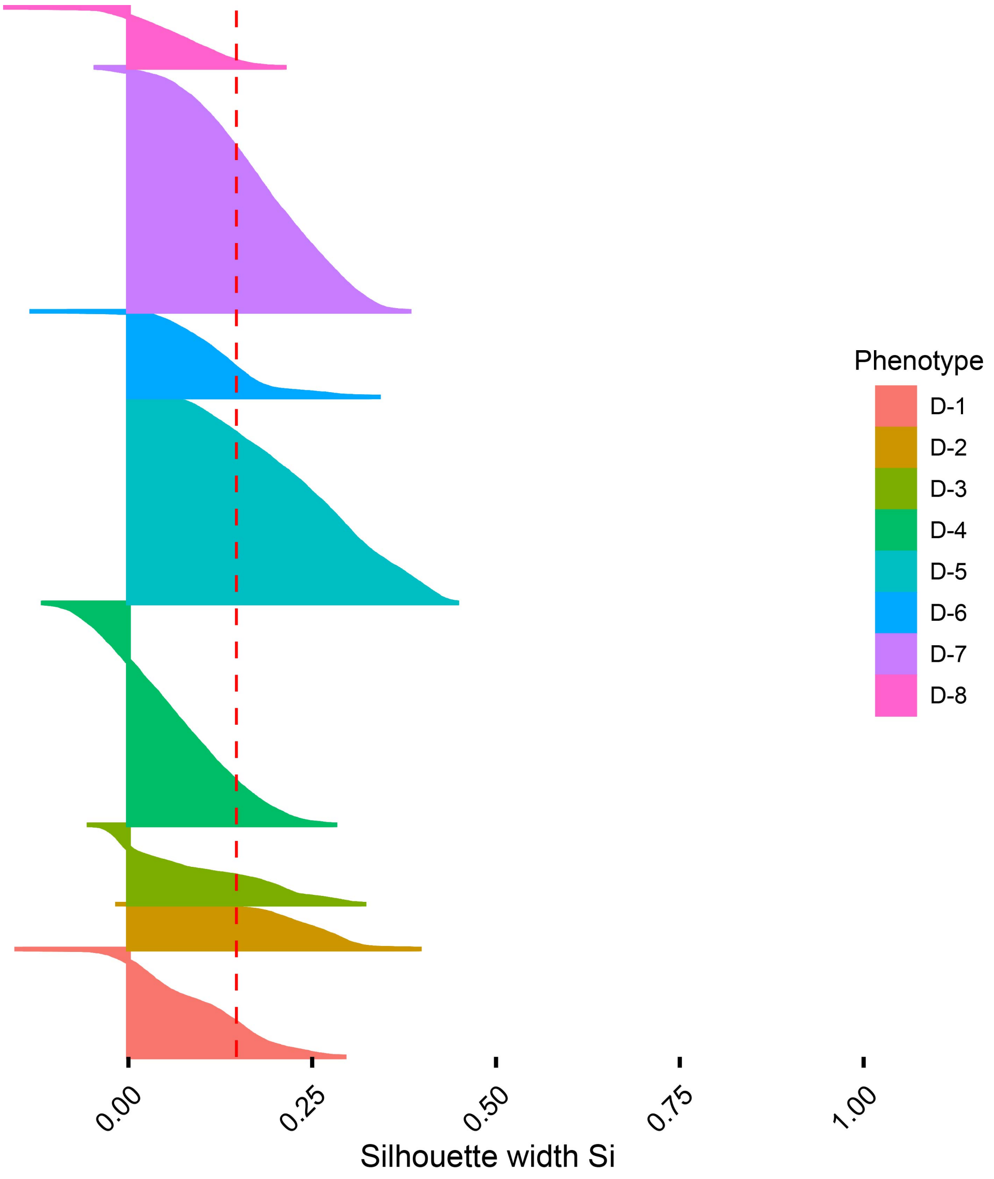

Silhouette plot in the derivation cohort (excluding the negative silhouette). The silhouette plot allows for a graphical evaluation of the clustering results. The silhouette value measures how similar an object is to others within its cluster versus those in other clusters. The values range from -1 (no similarity at all or wrong cluster assignment) to +1 (perfect clustering), with values close to 0 indicating that the item is on the boundary of two clusters and has poor separation performance. The silhouette thickness indicates the size of the sample to which it belongs and should not have a large bias. The silhouette coefficient for each cluster was calculated and this value was used as an indicator of how closely all data was grouped into one cluster. The red dotted line represents the average silhouette coefficient, which should be exceeded by each cluster. These results of the analysis are summarized in the silhouette plot.

Supplemental Figure.6 Outline of derivation cohort analysis

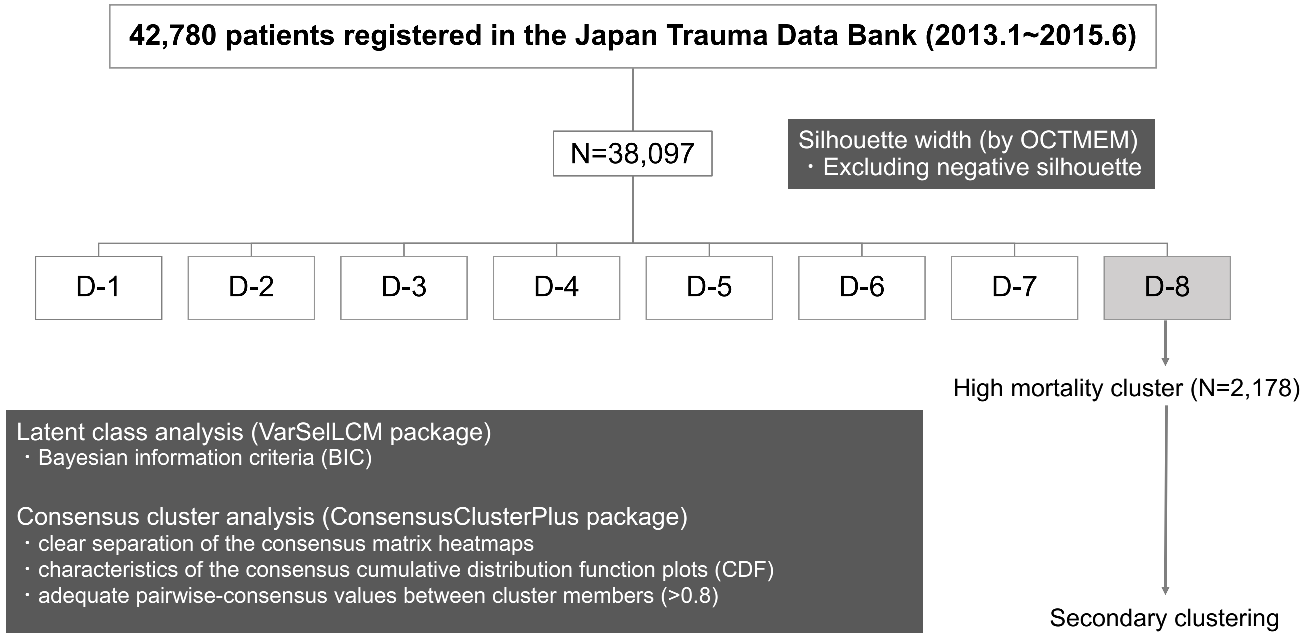

Silhouette analysis was performed in the derivation cohort and divided into eight clusters. Secondary clustering was performed on the cluster with the highest mortality (D-8) using Latent class analysis. Consensus Cluster Analysis was performed as a sensitivity analysis. Some analyses were performed on a supercomputer (OCTOPUS; Osaka University Cybermedia cenTer Over-Petascale Universal Supercomputer, job class: OCTMEM) because of their large computational complexity.

Supplemental Figure.7 Latent class analysis for high-mortality phenotype in the derivation cohort (Bayesian information criterion analysis)

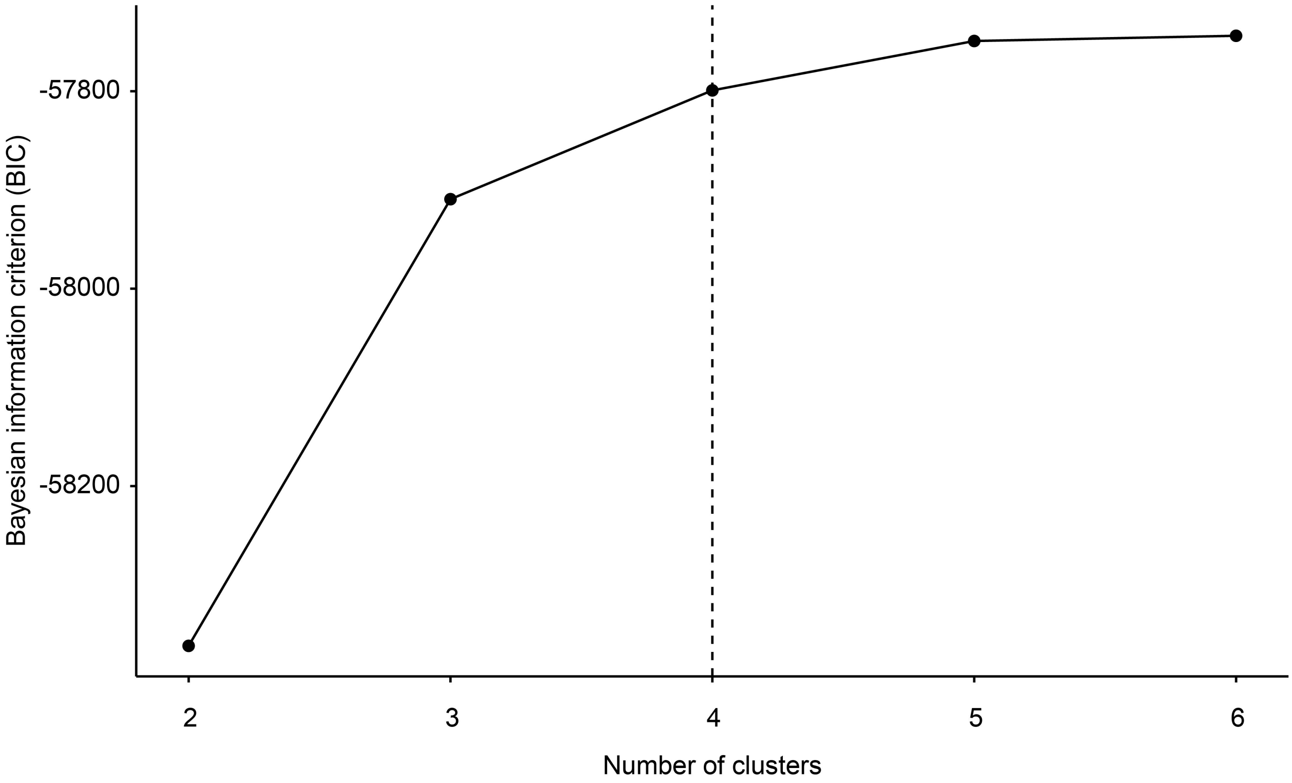

The model was fitted using derivation cohort and Bayesian Information Criterion (BIC) values were calculated. The optimal number of clinically significant subphenotypes was determined in terms of BIC value, moderate within-cluster sample size and high posterior probability of group assignment. Vertical dashed lines indicate the number of clusters determined.

Supplemental Figure.8 Probability of misclassification determined using latent class clustering for high-mortality group in the derivation cohort

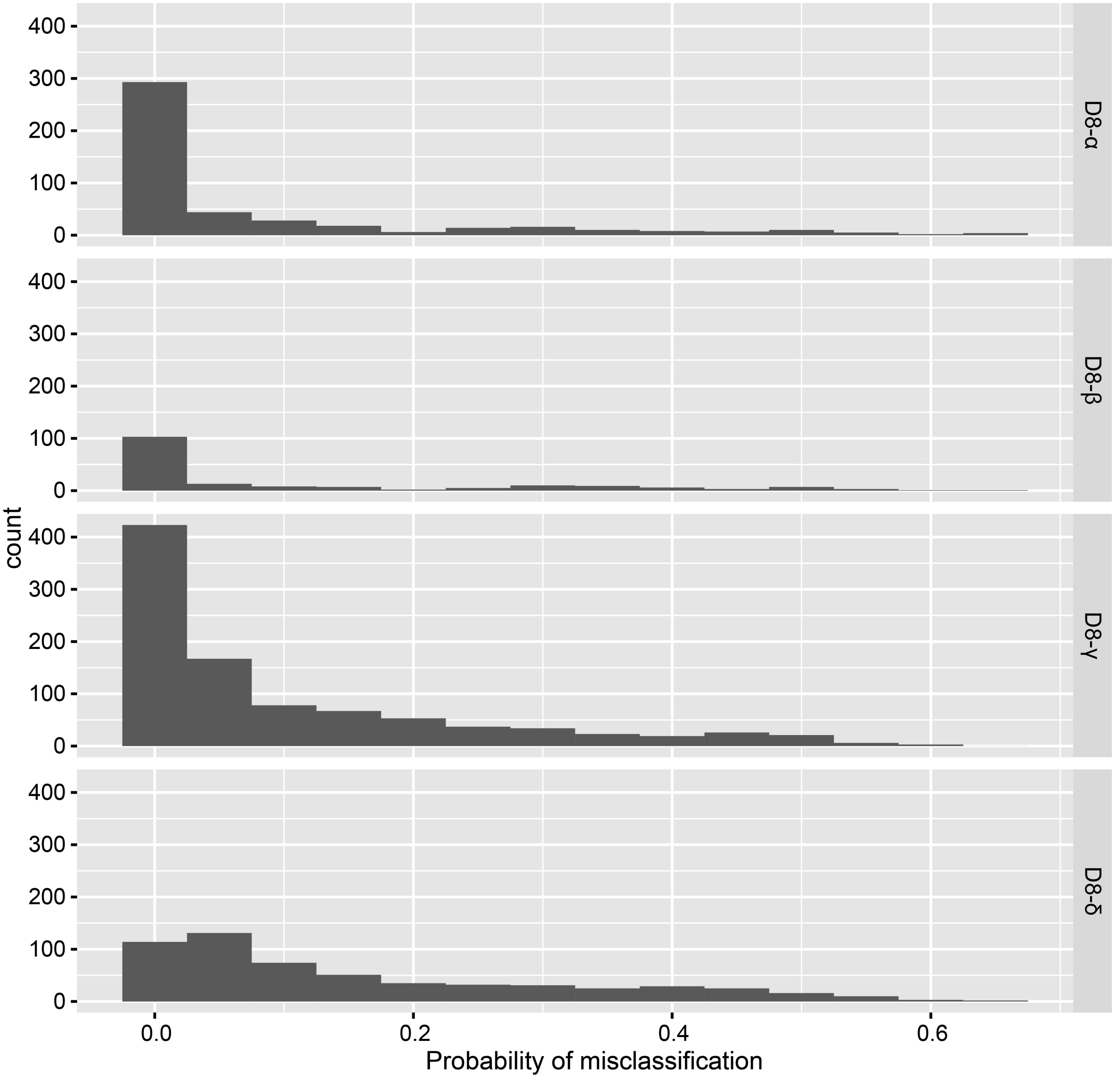

Histograms show the distribution of misclassification probabilities calculated using the posterior probabilities generated by LCA for the four phenotypes. Calculated by misclassification probability = 1 - (posterior probability attributed to each cluster). The histogram of intraphenotypic probabilities showed that members had a high probability (>0.9) of being phenotypic members.

Supplemental Figure.9 Probability of assignment for phenotype members and unassigned members determined using latent class analysis in the derivation cohort

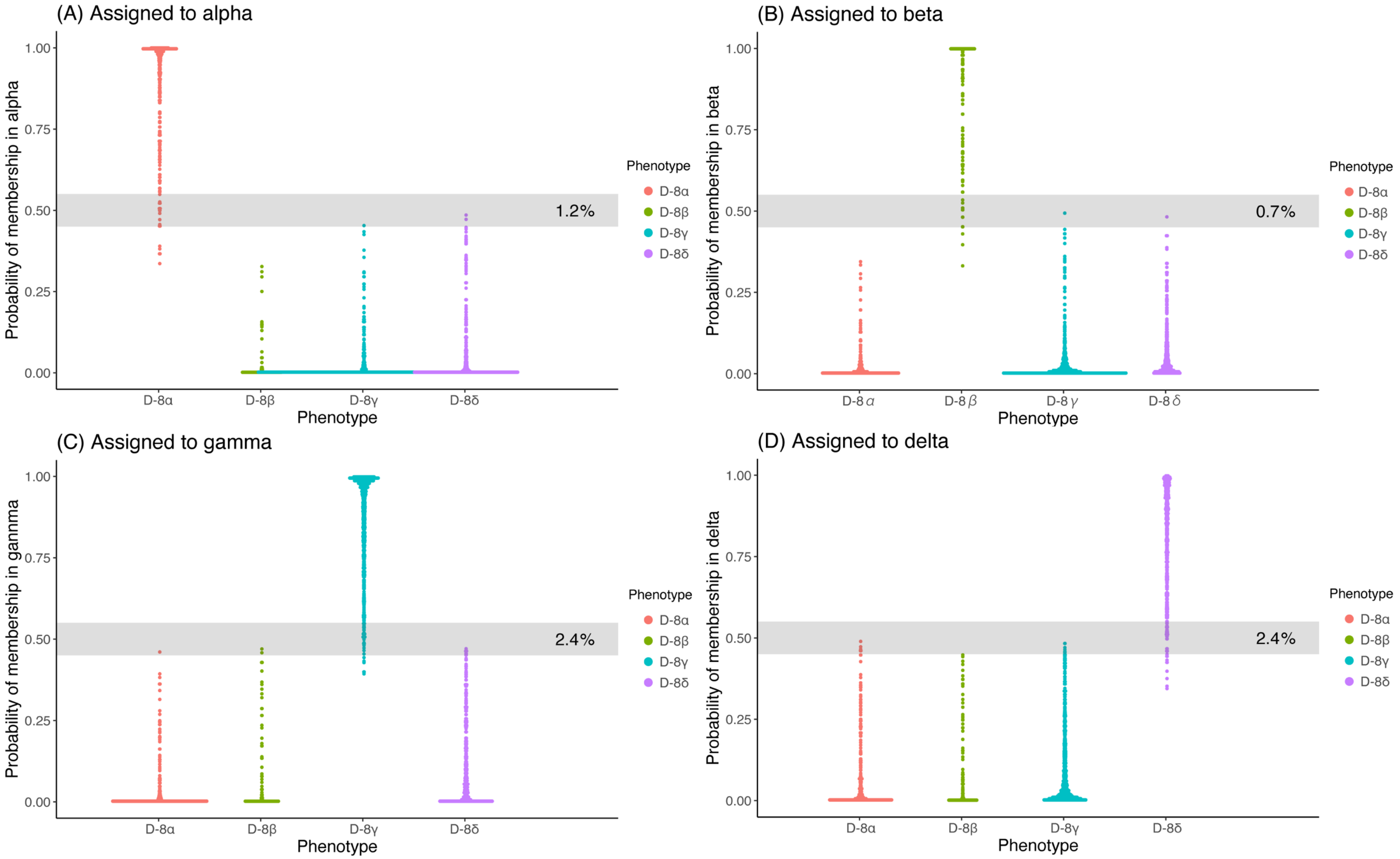

The probability that the subjects belong to each cluster is plotted from the posterior probabilities calculated by LCA; the fewer patients belonging to the margin region, the better the cluster can be interpreted as a cluster with better separation. Probabilities of assignment to the (a) alpha type (red indicates those actually assigned to alpha), (b) beta type (green), gamma type (blue), and (d) delta type (purple). Inset proportion is the percentage of 2,178 members in the marginal region. The figure shows that α-, β-, γ-, and δ-types are well separated and clustered.

Supplemental Figure.10 Discriminative power of each variable in the latent class analysis (derivation cohort)

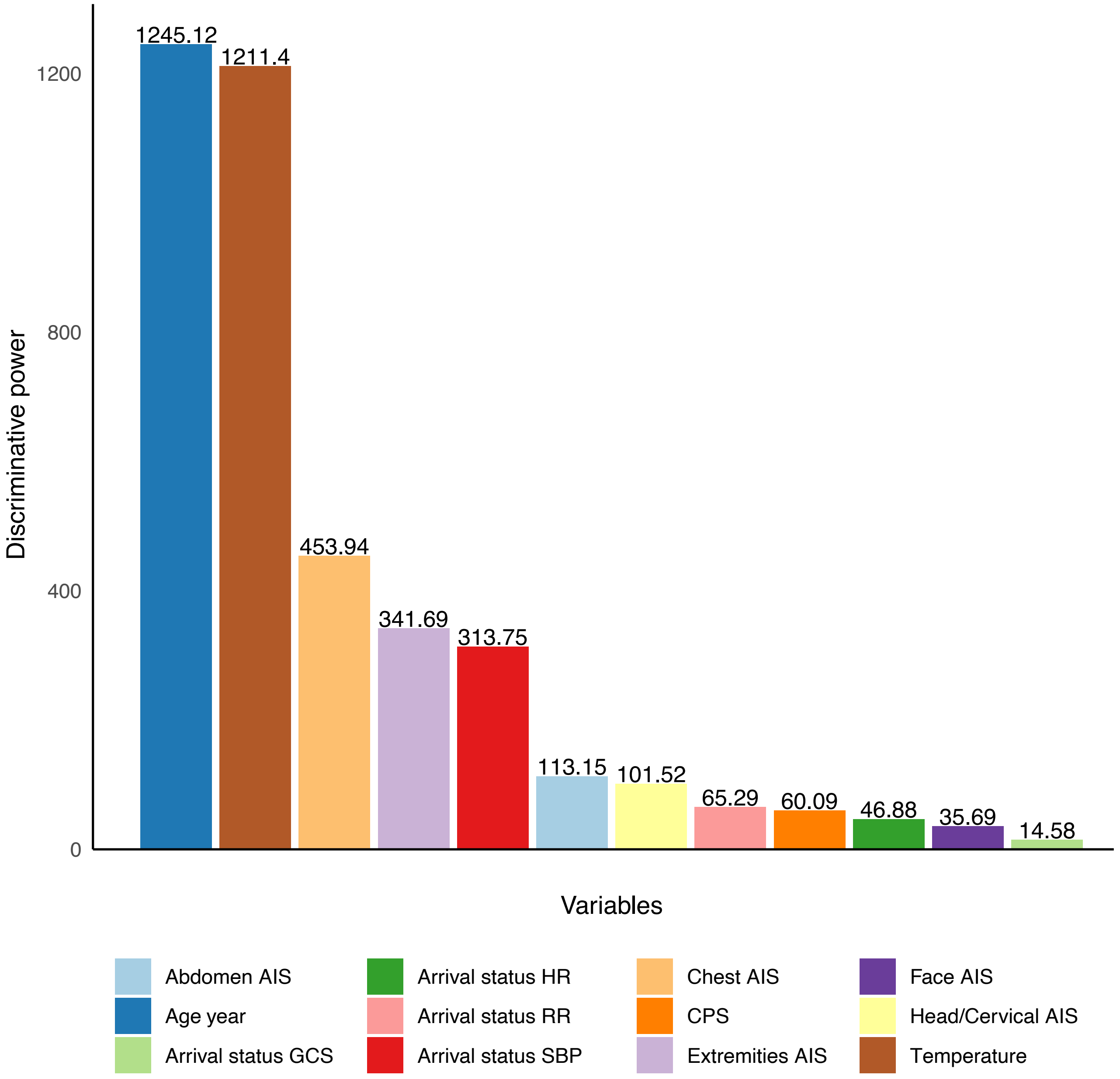

The discriminative power of each variable was calculated as the logarithm of the ratio of the probability that the variable is associated with clustering to the probability that it is not associated with clustering. Higher values indicate greater discriminatory power.

Supplemental Figure.11 Distribution of variables for each clinical phenotype (high-mortality group, derivation cohort)

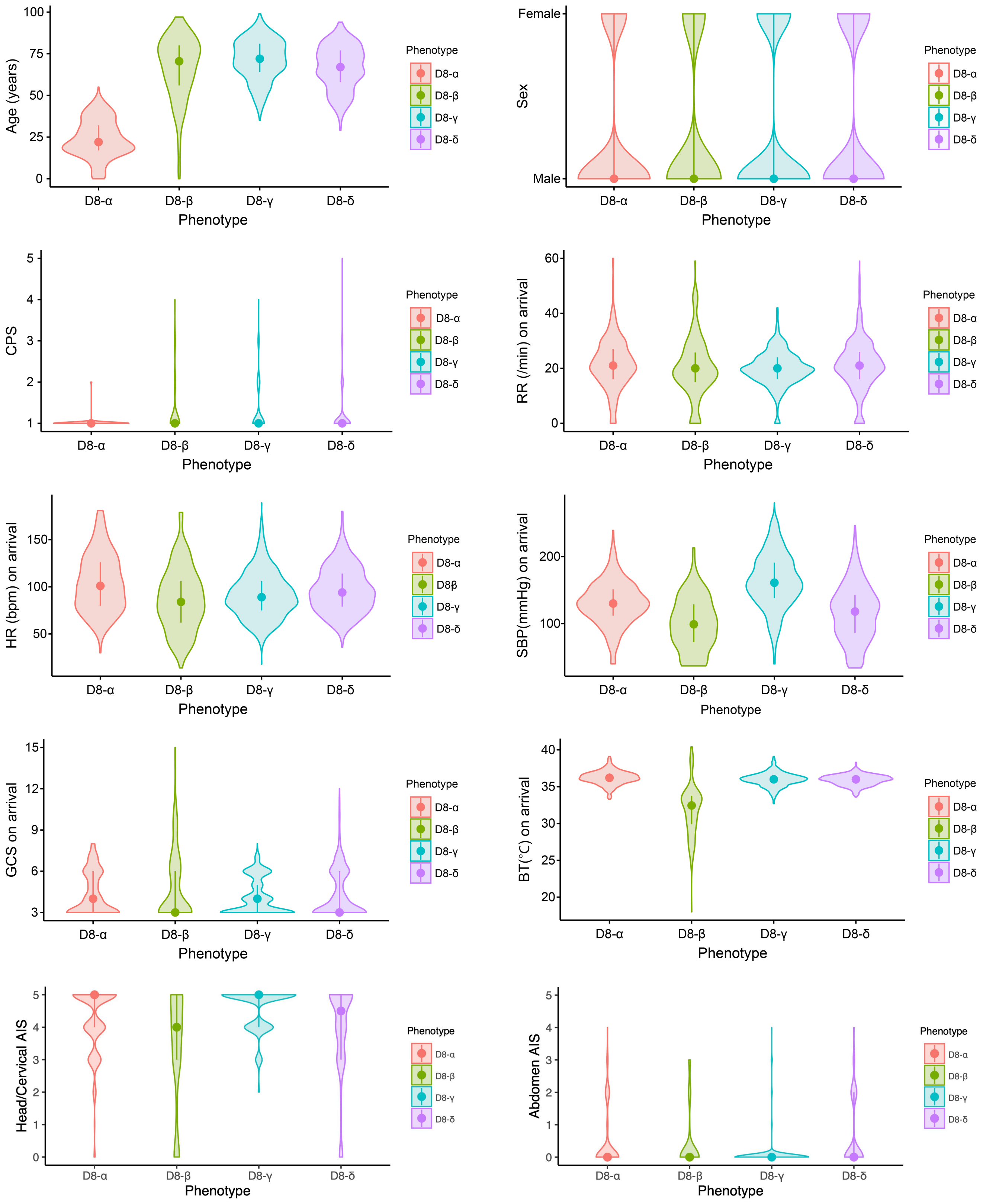

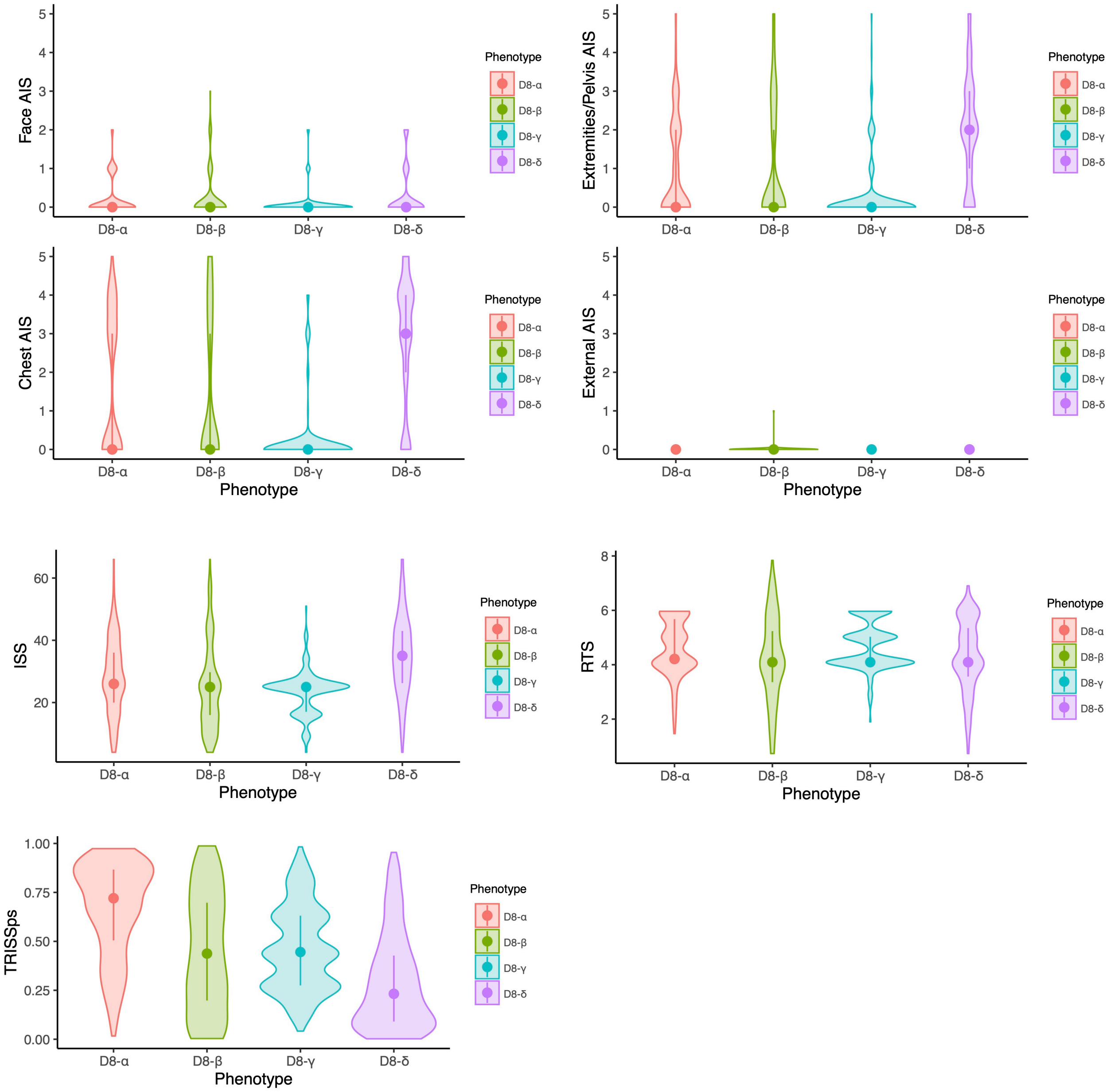

The distribution of each variable of the high-mortality phenotype in the derivation cohort is shown in a violin plot. The bar indicates the interquartile range, and the plot indicates the median.

Supplemental Figure.12 Alluvial plot showing distribution of clinical phenotypes in the derivation cohort

(A) Phenotype: D-8α

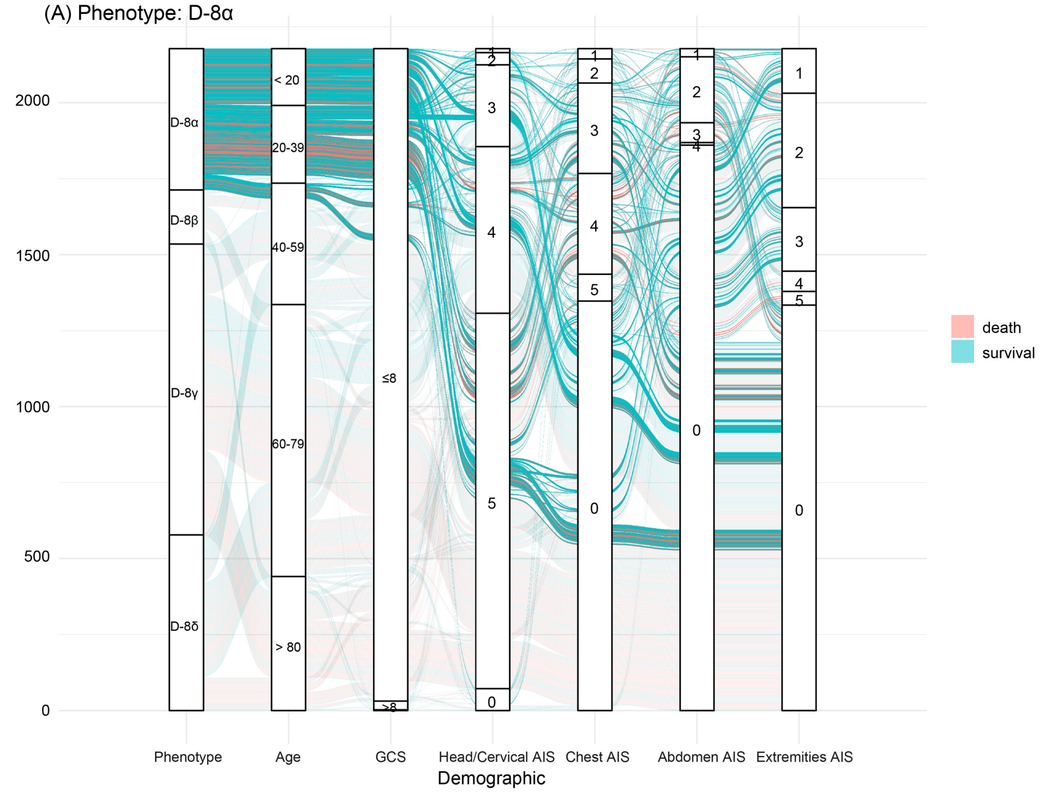

(B) Phenotype: D-8β

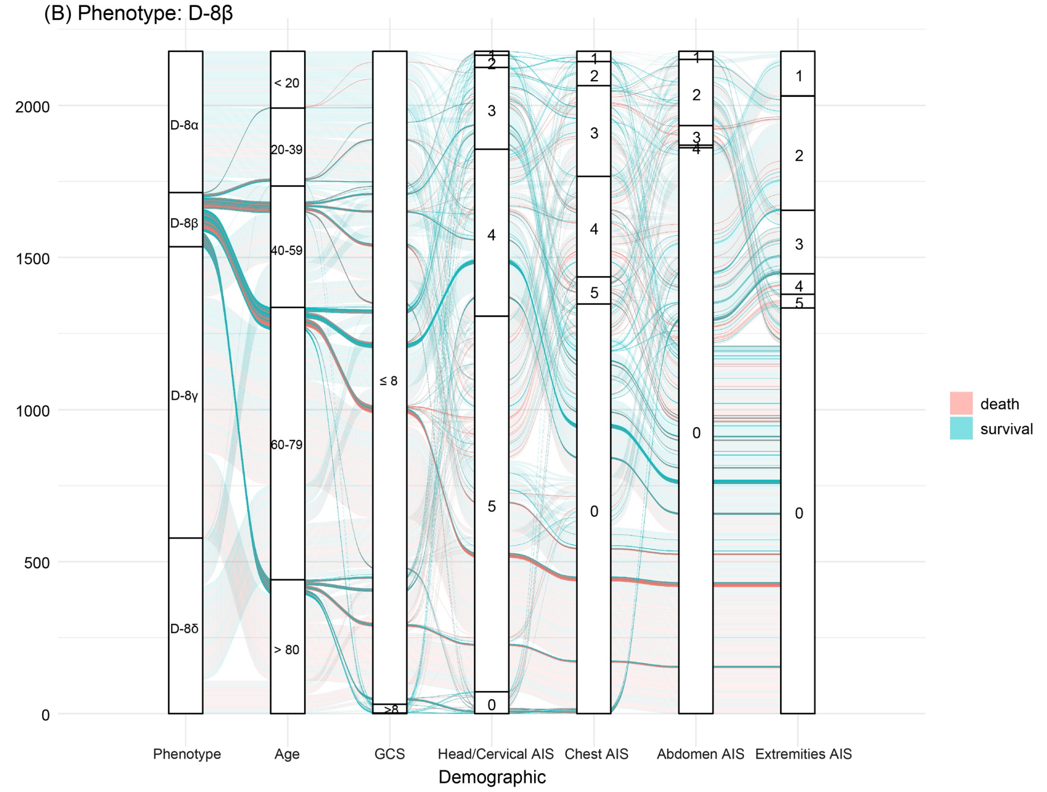

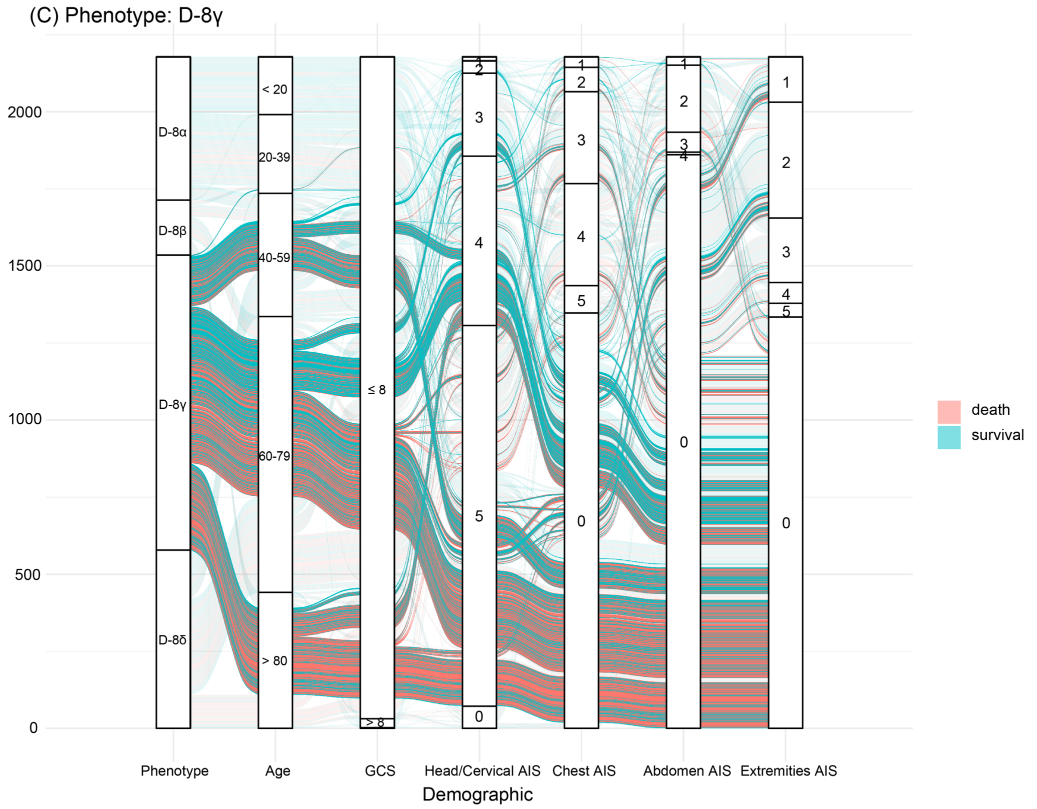
(C) Phenotype: D-8γ
2000
1500
1000
500
0
D-8α
D-8β
D-8γ
D-8δ
< 20
20-39
40-59
60-79
> 80
≤ 8
> 8
Phenotype
Age
GCS
Head/Cervical AIS
Chest AIS
Abdomen AIS
Extremities AIS
Demographic
death
survival

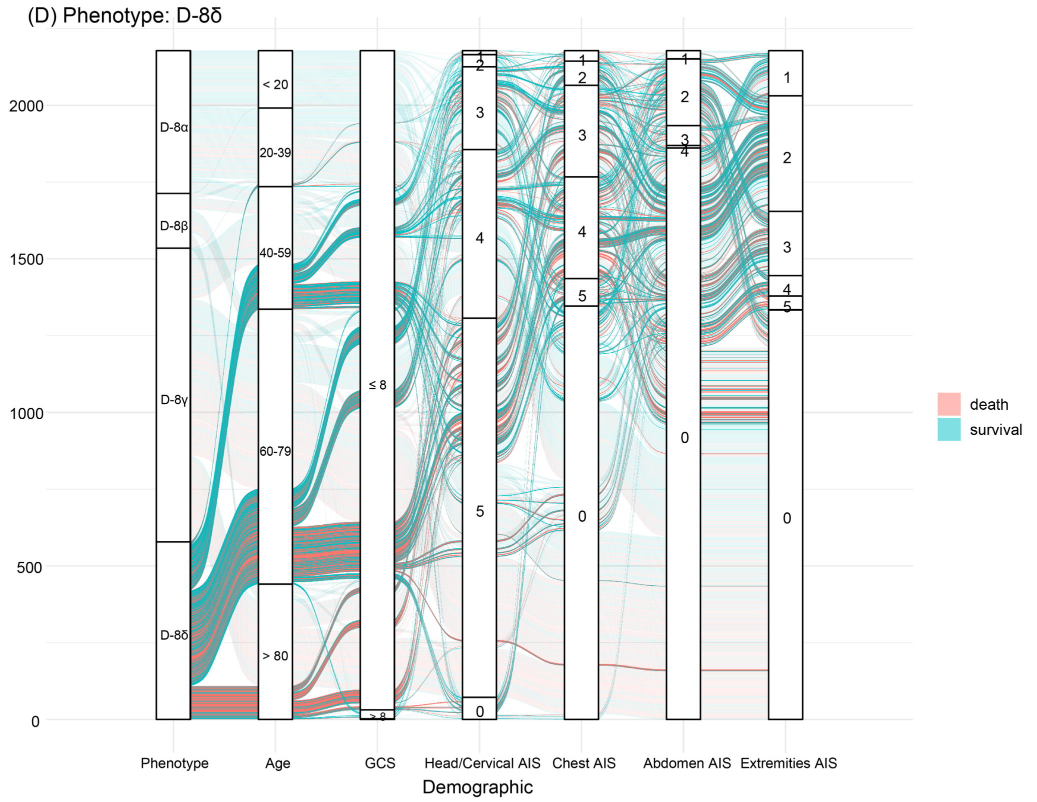
(D) Phenotype: D-8δ
2000
1500
1000
500
0
D-8α
D-8β
D-8γ
D-8δ
< 20
20-39
40-59
60-79
> 80
≤ 8
> 8
Phenotype
Age
GCS
Head/Cervical AIS
Chest AIS
Abdomen AIS
Extremities AIS
Demographic
death
survival

From the left column: clinical phenotype, age, GCS, head/cervical AIS, chest AIS, abdomen AIS, and extremities AIS. The blue bands indicate survivors, and the red bands indicate deceased. Sample sizes for each phenotype: D-8α (*n* = 464), D-8β (*n* = 178), D-8γ (*n* = 957), and D-8δ (*n* = 579). Interpretive example: in these alluvial plots, the members of the phenotype are shown in the leftmost column, and the distribution of each variable is shown in the subsequent columns. The D-8α phenotype is more common in younger patients who tend to have multiple traumas. A somewhat distinct band of mortality can be identified in some of the patients with head injuries with an AIS ≥4. AIS, Abbreviated Injury Scale; GCS; Glasgow Coma Scale.

Supplemental Figure.13 Consensus *k* clustering results in the derivation cohort

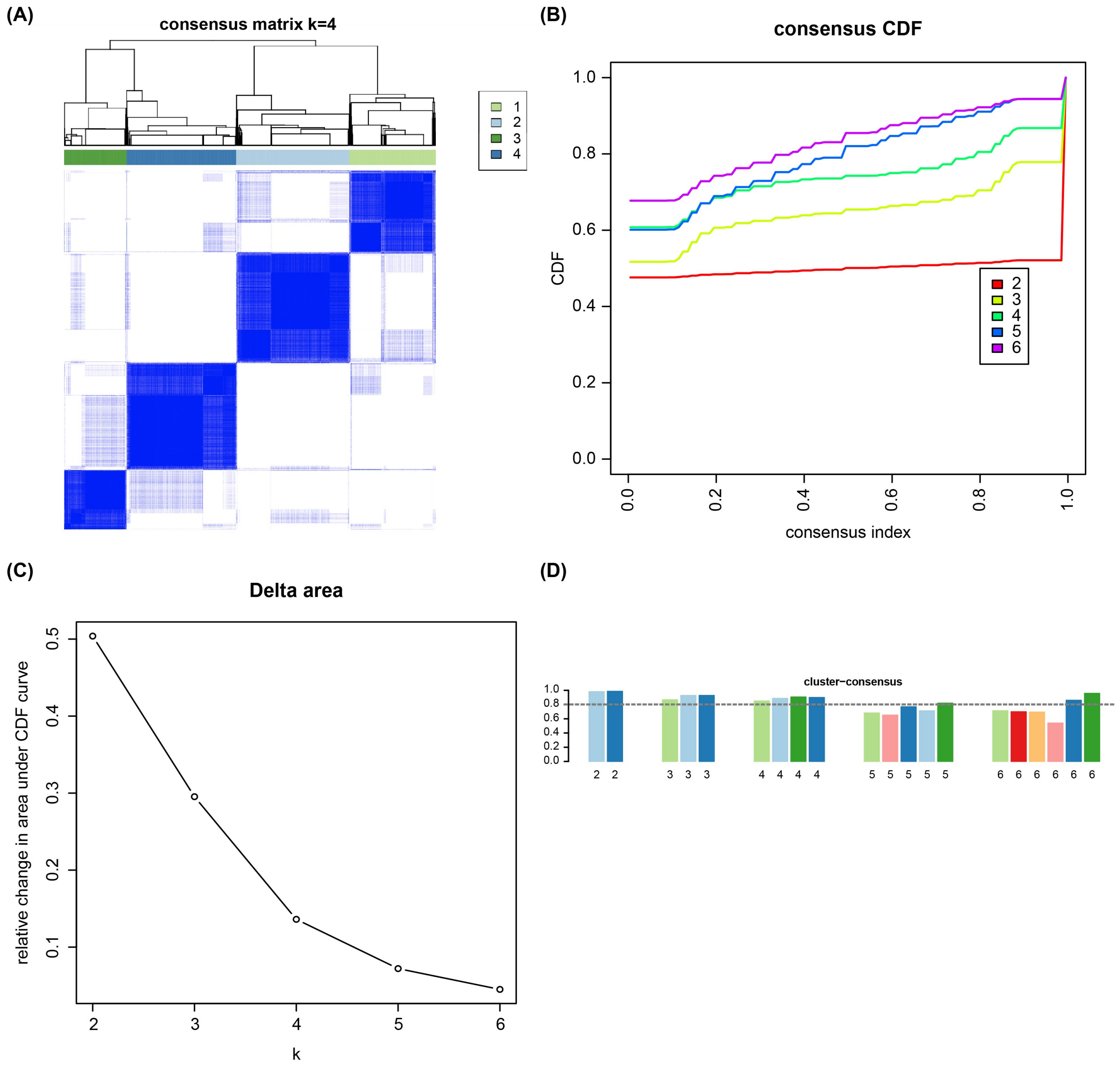

(a) Unsupervised consensus *k*-means clustering in the high-mortality phenotype of the derivation cohort (n = 2,178) and showing optimal partitioning for the $k = 4$ consensus matrix. (b) Consensus cumulative distribution function (CDF) plot across $k = 2$ to $k = 6$, where higher and more horizontal curves suggest an optimal fit. (c) Relative change in the area under the CDF curve with increasing clusters (k), with little change observed from $k = 2$ to $k = 4$. (d) Cluster consensus plot from $k = 2$ to $k = 6$ and showing the mean of all pairwise consensus values between cluster members. A $k = 4$ implied that the consensus value was >0.8 for all clusters and suggesting an optimal fit. Based on the overall judgment of (a)-(d), k=4 was determined to be the optimal cluster.

Supplemental Figure.14 t-SNE plot of clinical phenotype assignments in the derivation cohort

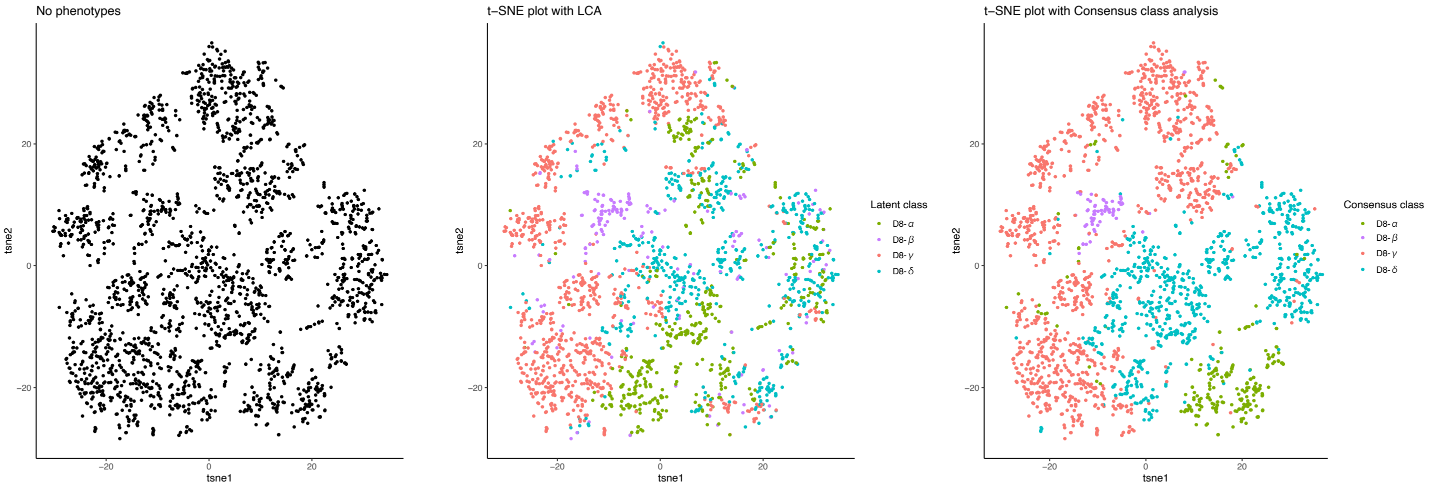

(a) Visualization of phenotypes using the t-SNE method in the high-mortality phenotype group of the derivation cohort. Phenotypes are not shown in color. (b) Color coding based on latent class analysis. (c) Sensitivity analysis using consensus class analysis. Interpretive example: using visualization methods, the phenotype members showed that the clustering results were similar between the two different methods (latent class analysis and consensus class analysis). t-SNE, t-distributed stochastic neighbor embedding.

Supplemental Figure.15 Kaplan-Meier plot for the derivation cohort

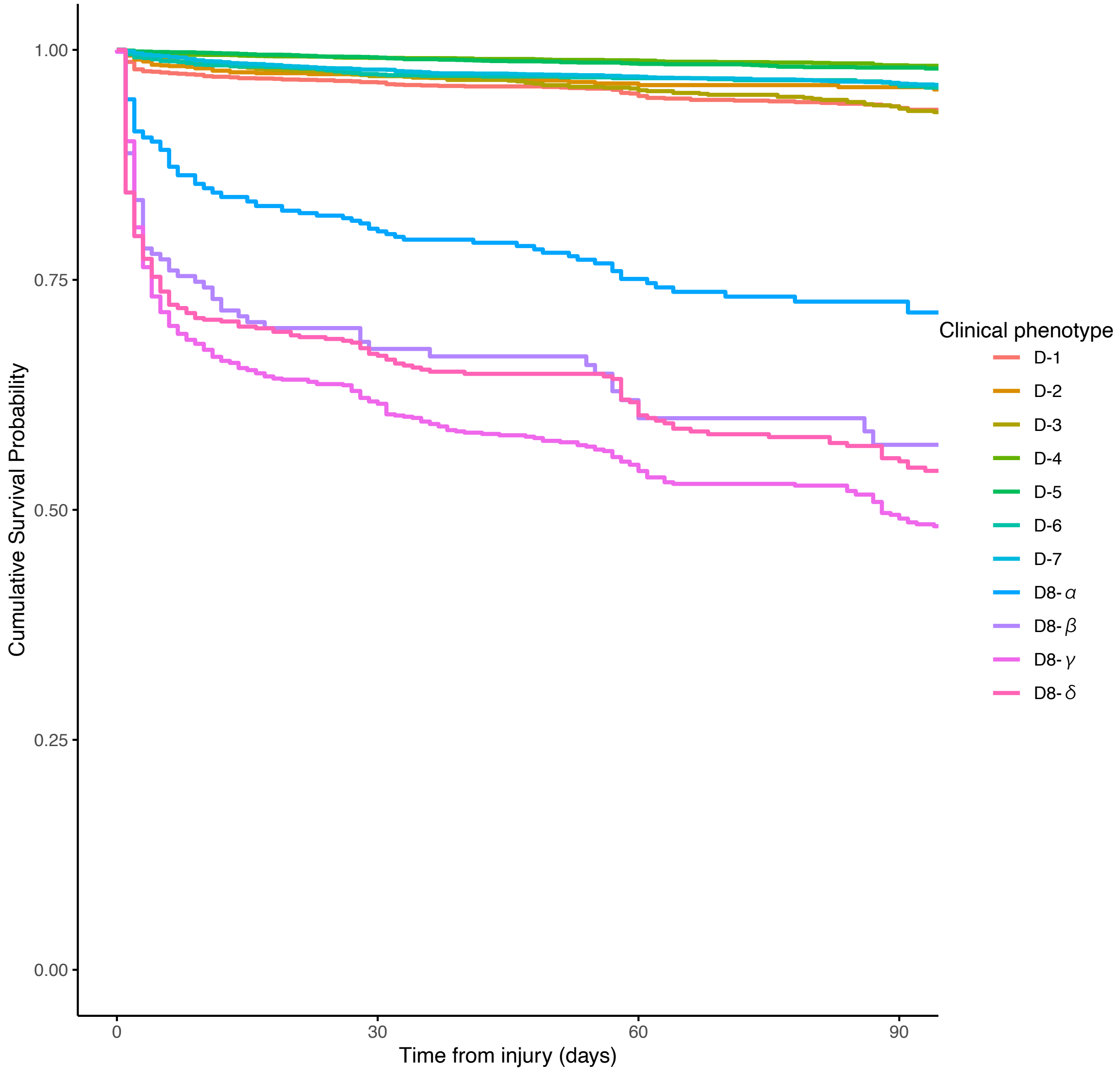

The Kaplan–Meier curves for each phenotype in the derivation cohort. The vertical axis shows the cumulative probability of survival, and the horizontal axis shows the number of days from injury to death.

Supplemental Figure.16 Optimal number of clusters in the validation cohort (average silhouette width)

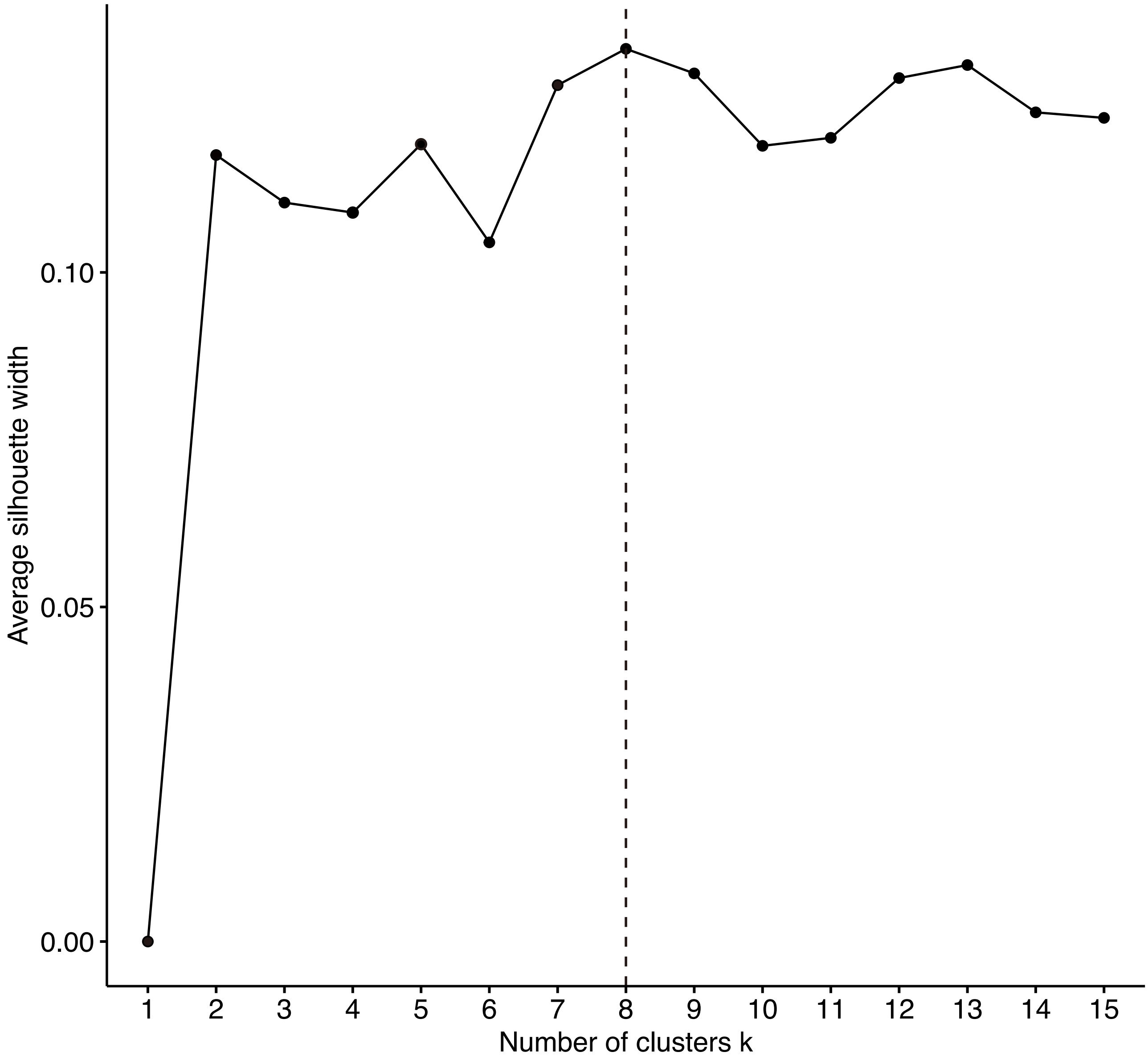

The silhouette method was used in the validation cohort. The silhouette method was used to evaluate how well each individual fit within their cluster and estimate the mean distance between clusters. The silhouette coefficients ranged from −1 to +1, where a high value indicates that individuals are well-matched to their clusters and poorly matched to neighboring clusters. A high average silhouette width indicates a good clustering. The vertical dashed line indicates that the mean silhouette width as the highest for $k = 8$.

Supplemental Figure.17 Optimal number of clusters in the validation cohort (elbow plot by k-means)

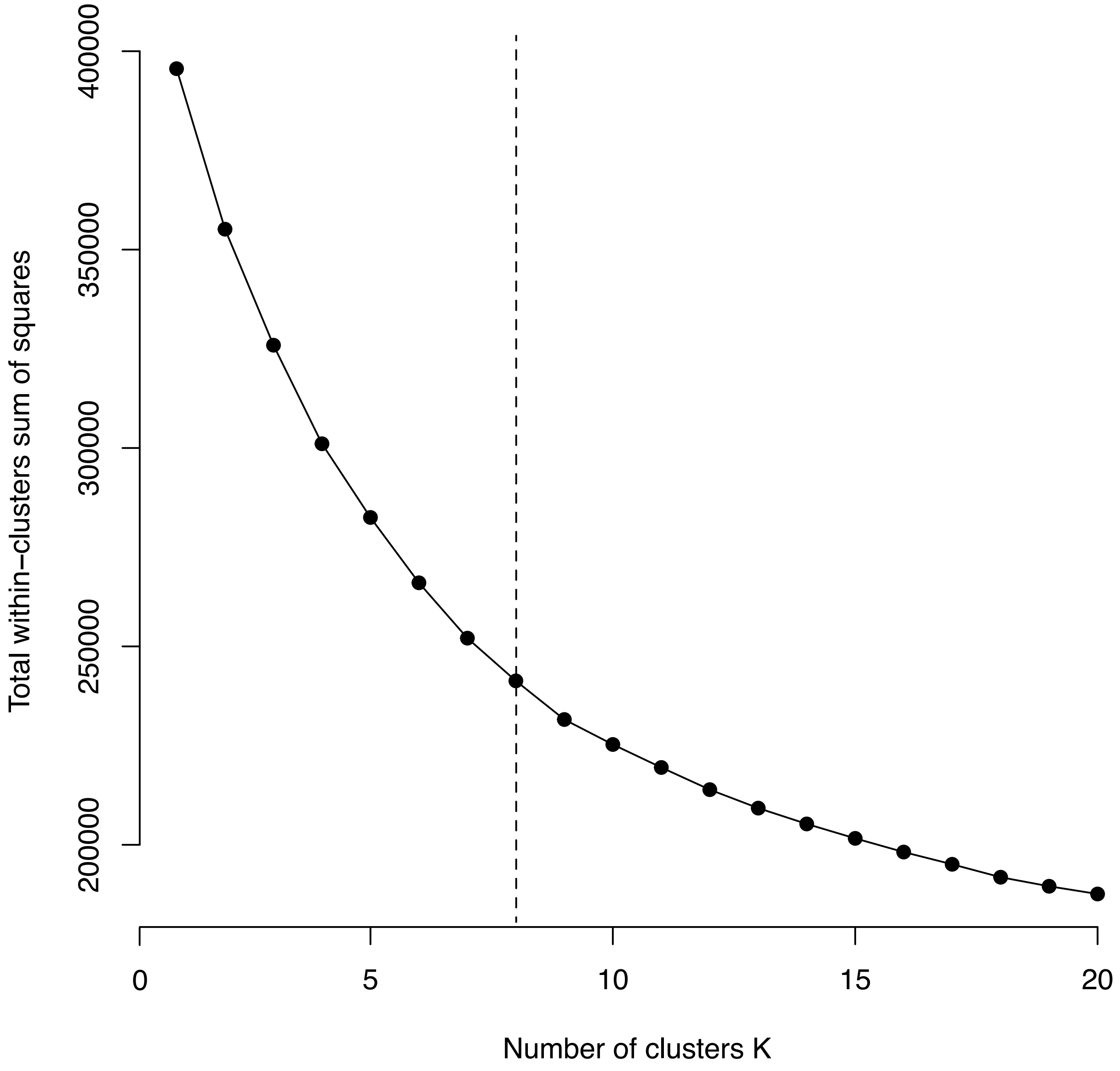

The *k*-means algorithm was used for the validation cohort. The elbow plot was generated by calculating the within-cluster sum of squares (*k* range: 1–20) in the validation cohort. The location of a bend (elbow) in the plot is generally considered as an indicator of the appropriate number of clusters. The vertical dashed line indicates $k = 8$, with the elbow located near this area.

Supplemental Figure.18 Silhouette plot for the validation cohort (excluding the negative silhouette)

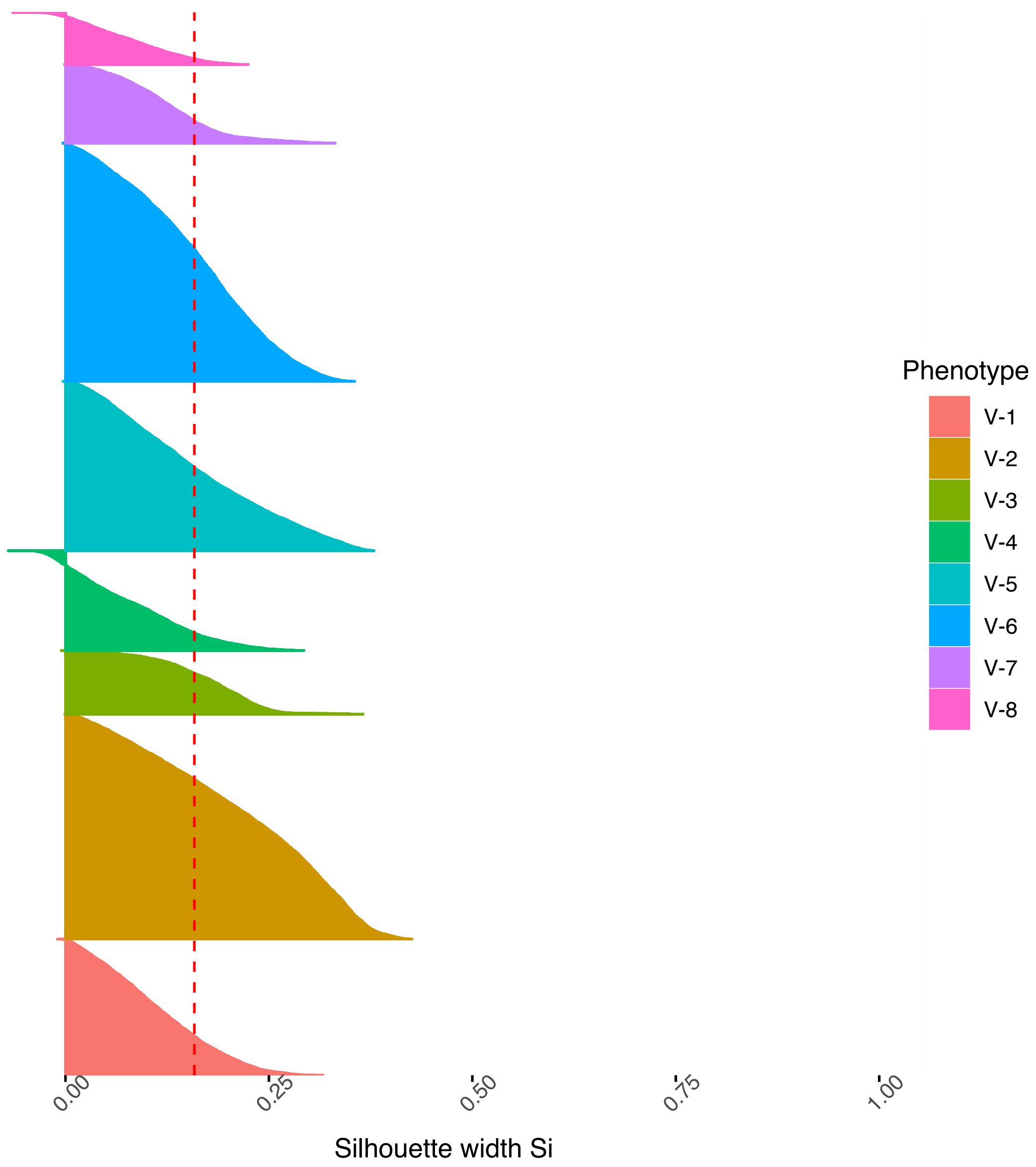

The silhouette plot allows for a graphical evaluation of the clustering results. The silhouette value measures how similar an object is to others within its cluster versus those in other clusters. The values range from -1 (no similarity at all or wrong cluster assignment) to +1 (perfect clustering), with values close to 0 indicating that the item is on the boundary of two clusters and has poor separation performance. The silhouette thickness indicates the size of the sample to which it belongs and should not have a large bias. The silhouette coefficient for each cluster was calculated and this value was used as an indicator of how closely all data was grouped into one cluster. The red dotted line represents the average silhouette coefficient, which should be exceeded by each cluster. These results of the analysis are summarized in the silhouette plot.

Supplemental Figure.19 Outline of validation cohort analysis

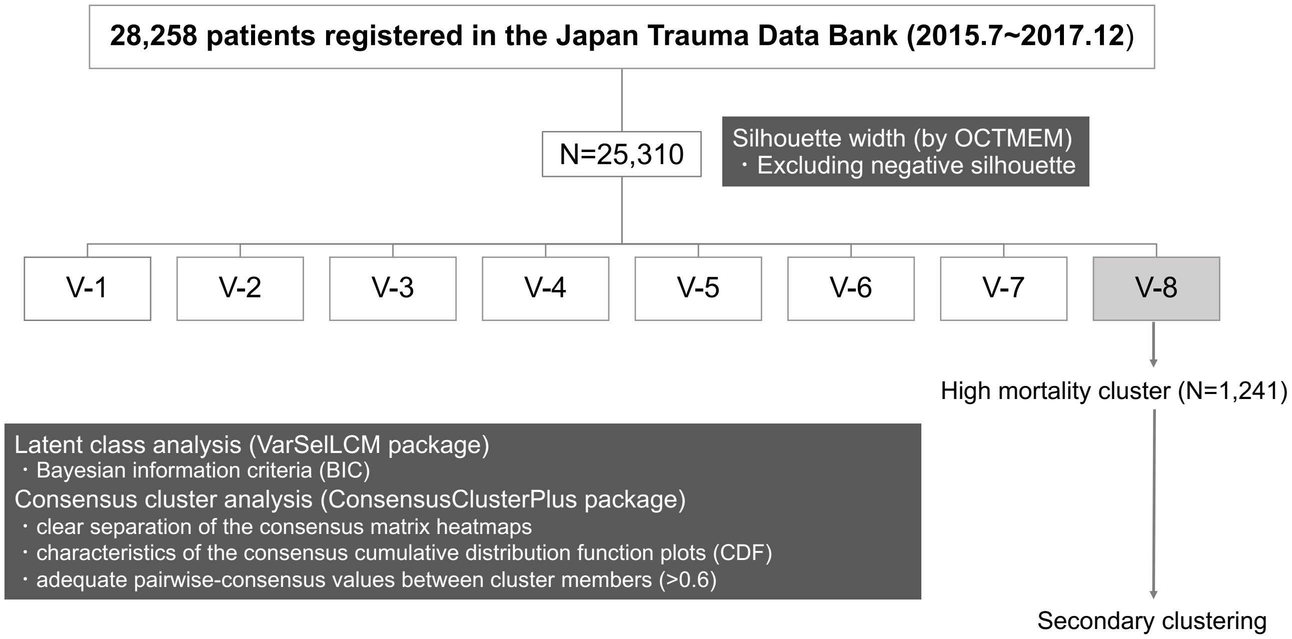

Silhouette analysis was performed in the validation cohort and divided into eight clusters. Secondary clustering was performed on the cluster with the highest mortality (V-8) using Latent class analysis. Consensus Cluster Analysis was performed as a sensitivity analysis. Some analyses were performed on a supercomputer (OCTOPUS; Osaka University Cybermedia cenTer Over-Petascale Universal Supercomputer, job class: OCTMEM) because of their large computational complexity.

Supplemental Figure.20 Latent class analysis for high-mortality phenotype in the validation cohort (Bayesian information criterion analysis)

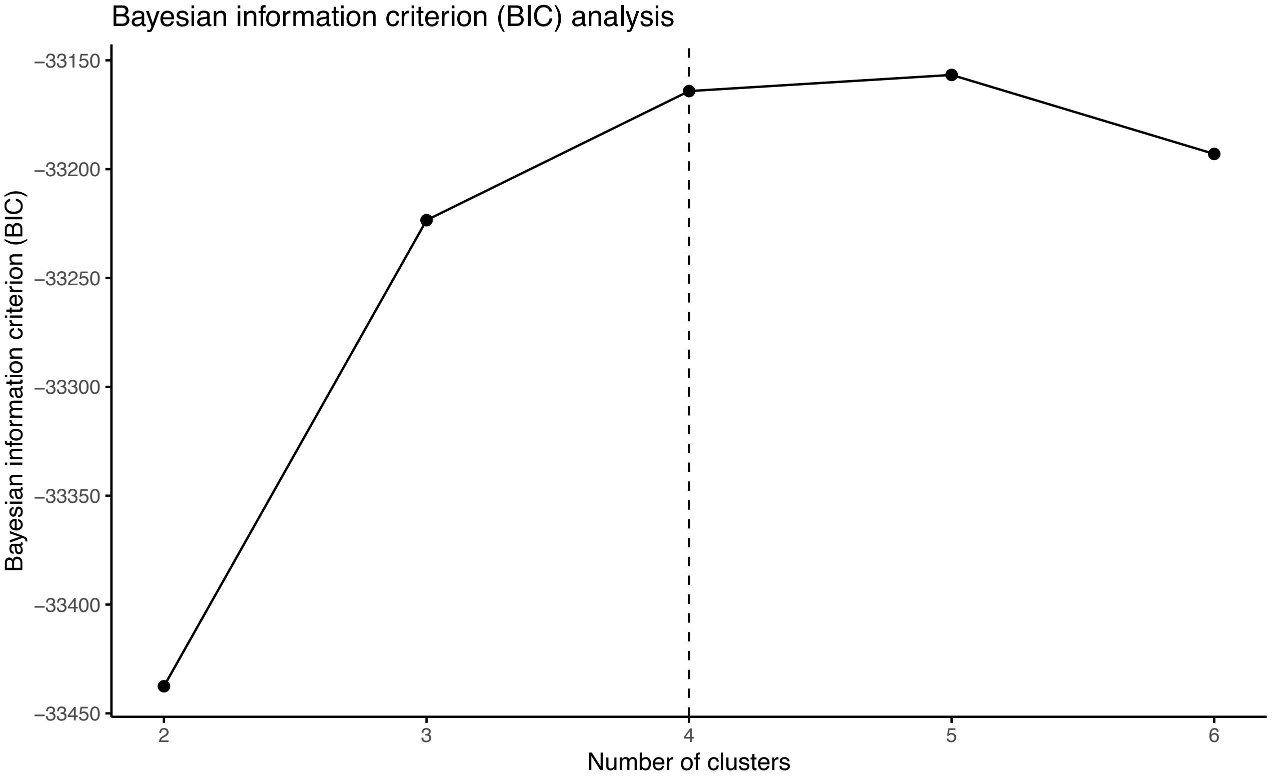

The model was fitted using validation cohort and Bayesian Information Criterion (BIC) values were calculated. The optimal number of clinically significant subphenotypes was determined in terms of BIC value, moderate within-cluster sample size and high posterior probability of group assignment. Vertical dashed lines indicate the number of clusters determined.

Supplemental Figure.21 Probability of misclassification determined using latent class clustering for the high-mortality group in the validation cohort

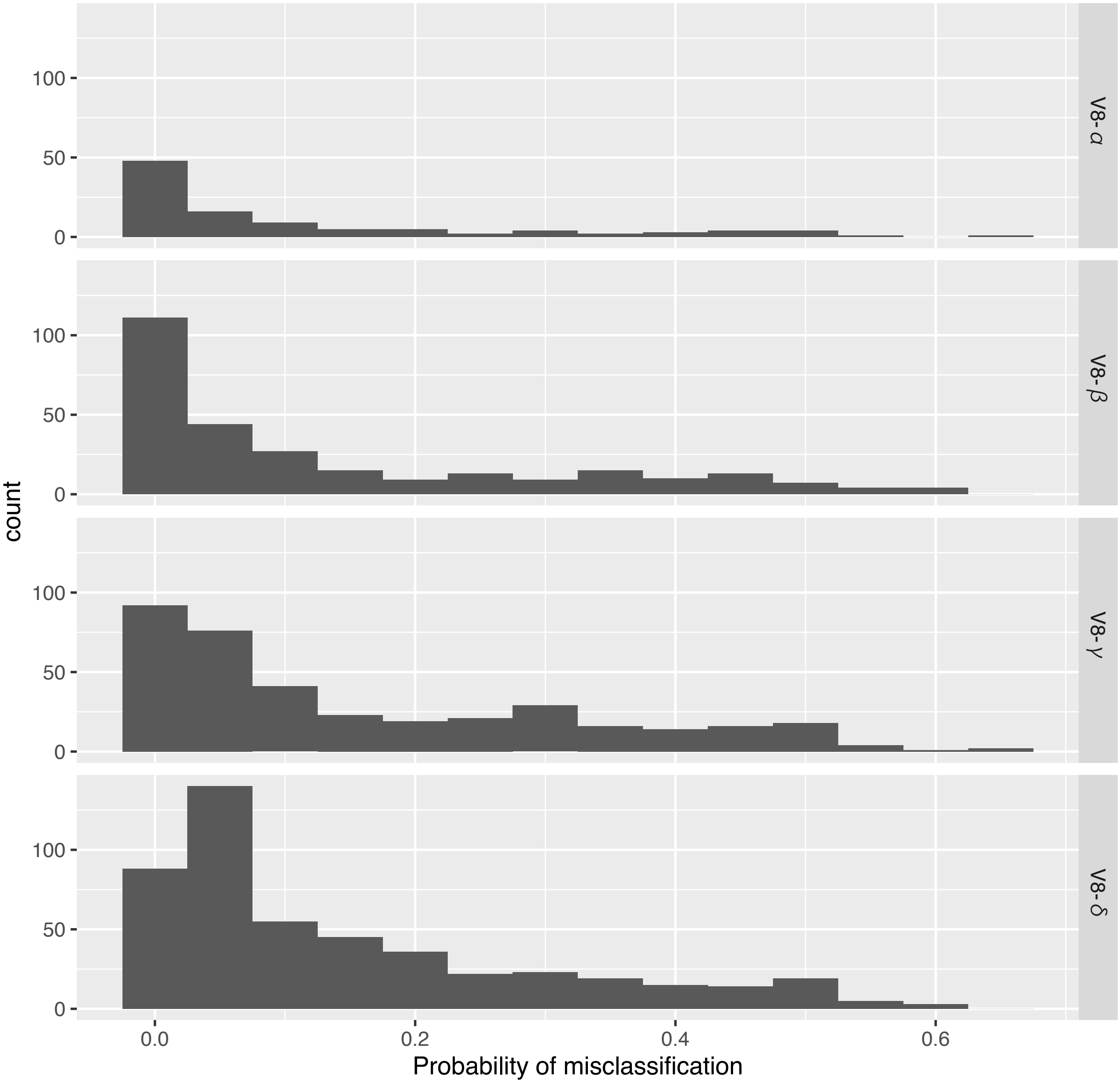

Histograms show the distribution of misclassification probabilities calculated using the posterior probabilities generated by LCA for the four phenotypes. Calculated by misclassification probability = 1 - (posterior probability attributed to each cluster). The histogram of intraphenotypic probabilities showed that members had a high probability (>0.9) of being phenotypic members.

Supplemental Figure.22 Probability of assignment for phenotype members and unassigned members determined using latent class analysis in the validation cohort

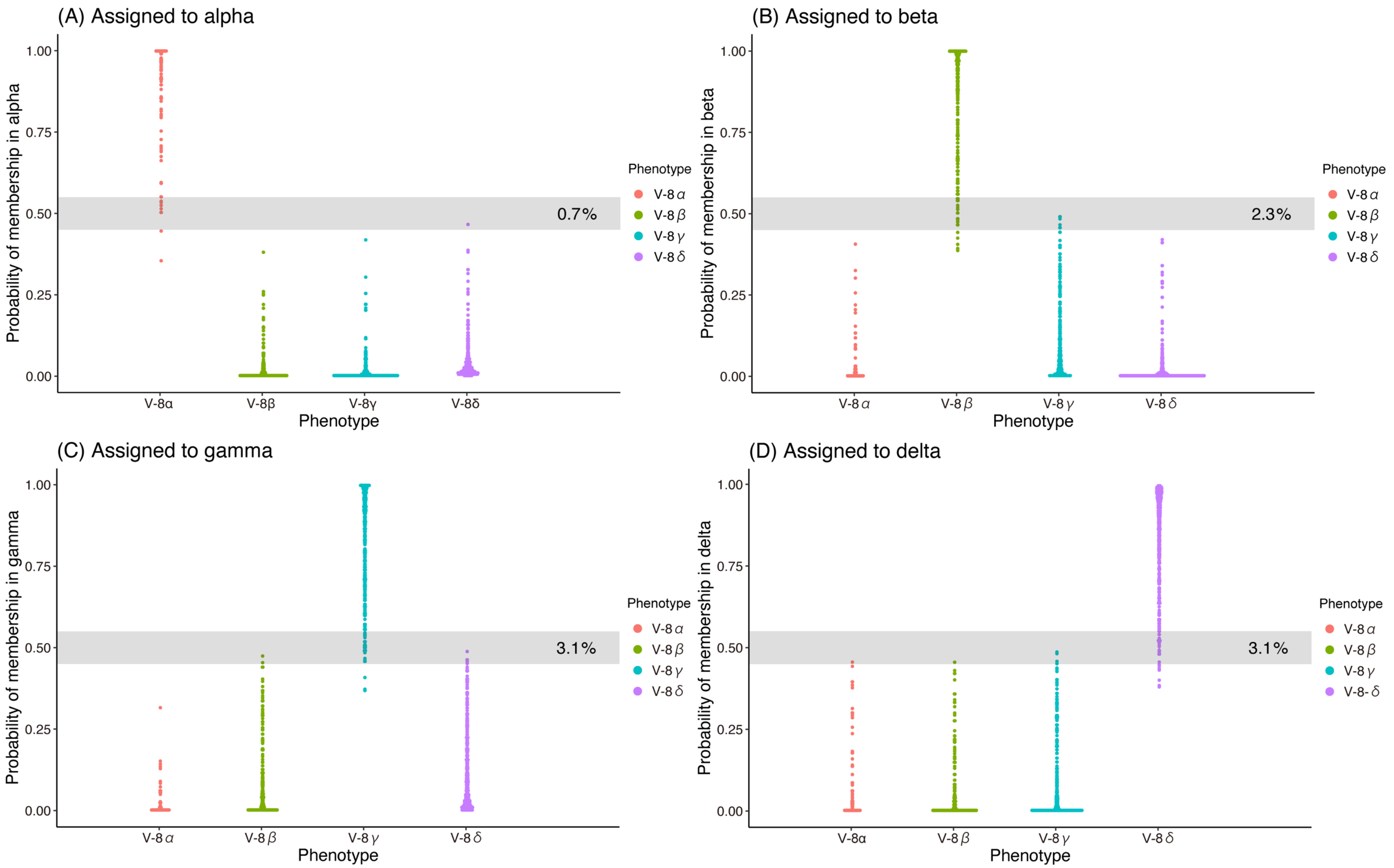

The probability that the subjects belong to each cluster is plotted from the posterior probabilities calculated by LCA; the fewer patients belonging to the margin region, the better the cluster can be interpreted as a cluster with better separation. Probabilities of assignments to (a) alpha type (red indicates those actually assigned to alpha), (b) beta type (green), gamma type (blue), and (d) delta type (purple). Inset proportion is the percentage of 1,241 members in the marginal region. The figure shows that α-, β-, γ-, and δ-types are well separated and clustered

Supplemental Figure.23 Discriminative power of each variable in the latent class analysis (validation cohort)

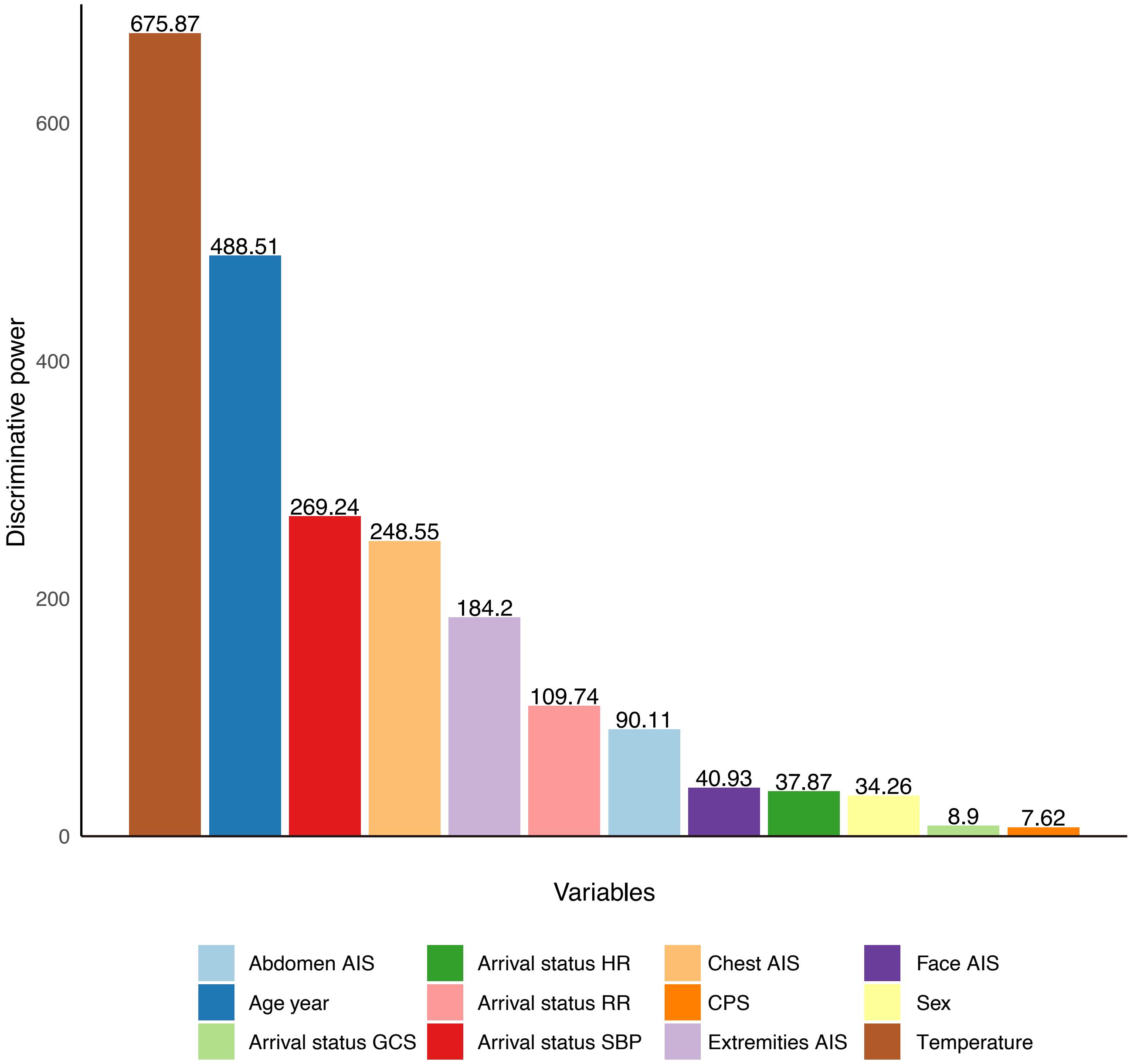

The discriminative power of each variable was calculated as the logarithm of the ratio of the probability that the variable is associated with clustering to the probability that it is not associated with clustering. Higher values indicate greater discriminatory power.

Supplemental Figure.24 Distribution of variables for each clinical phenotype (high-mortality group, validation cohort)

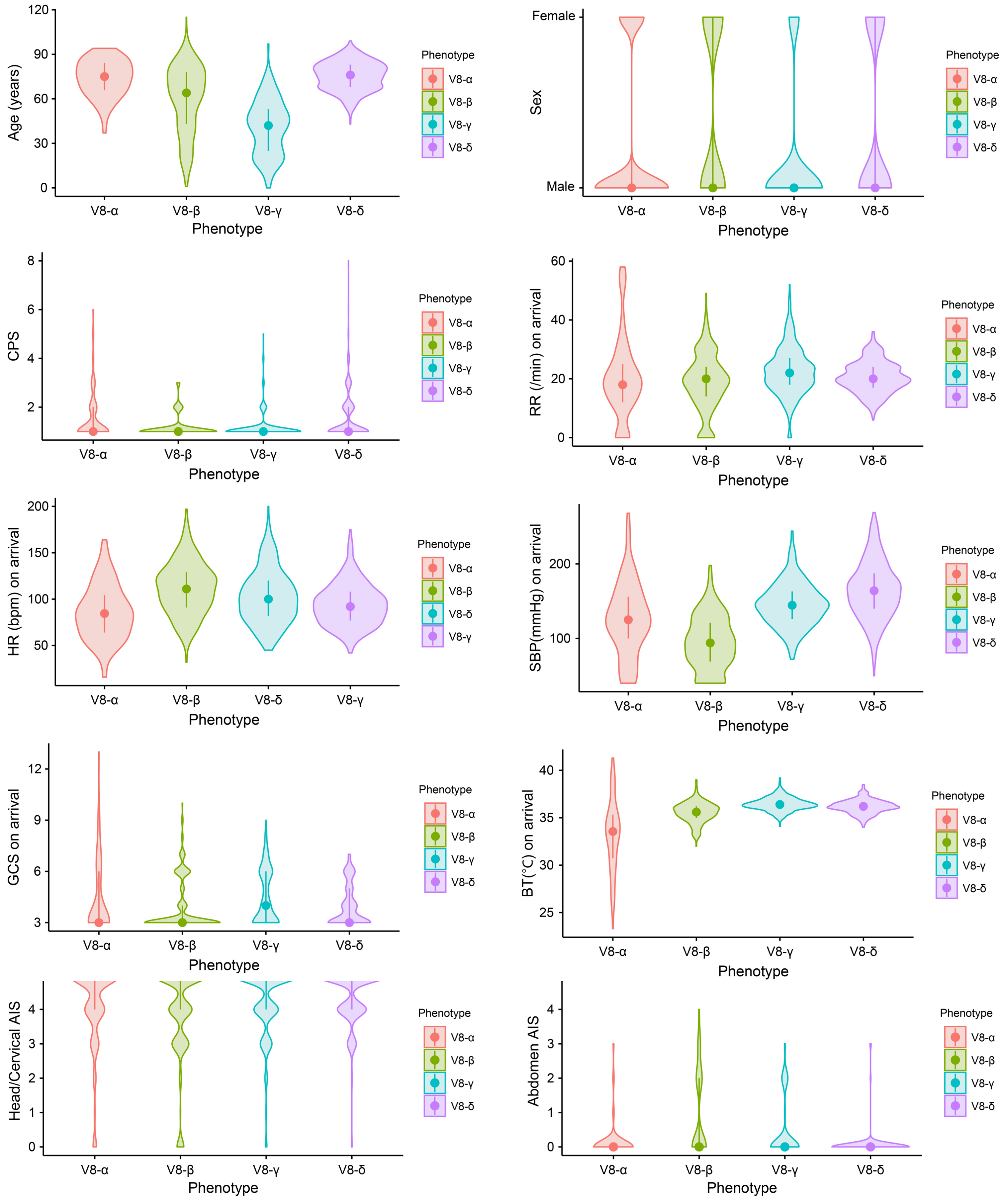

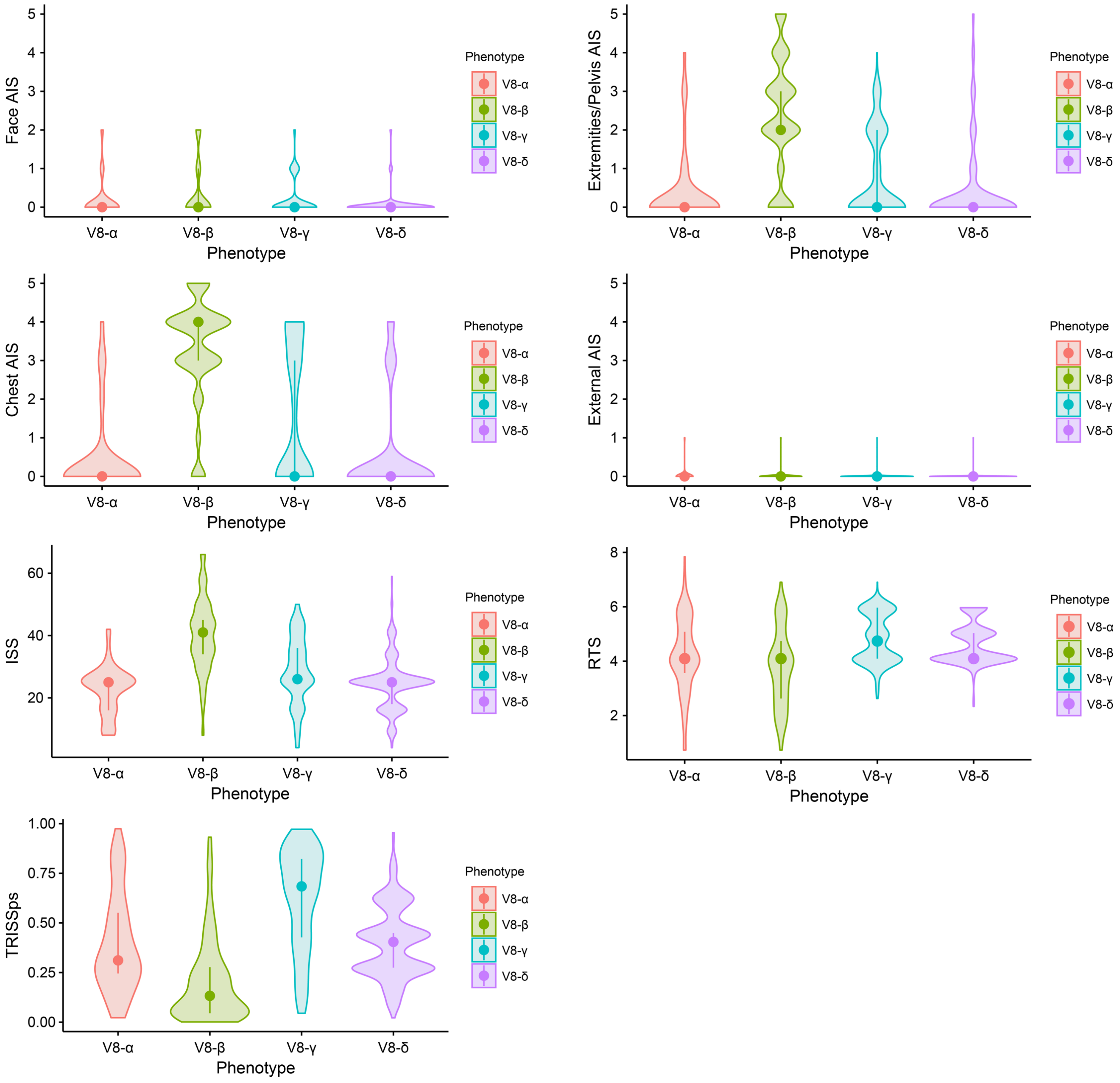

The distribution of each variable of the high-mortality phenotype in the validation cohort is shown in the violin plot. The bar indicates the interquartile range, and the plot indicates the median.

Supplemental Figure.25 Alluvial plot showing distribution of clinical phenotypes in the validation cohort

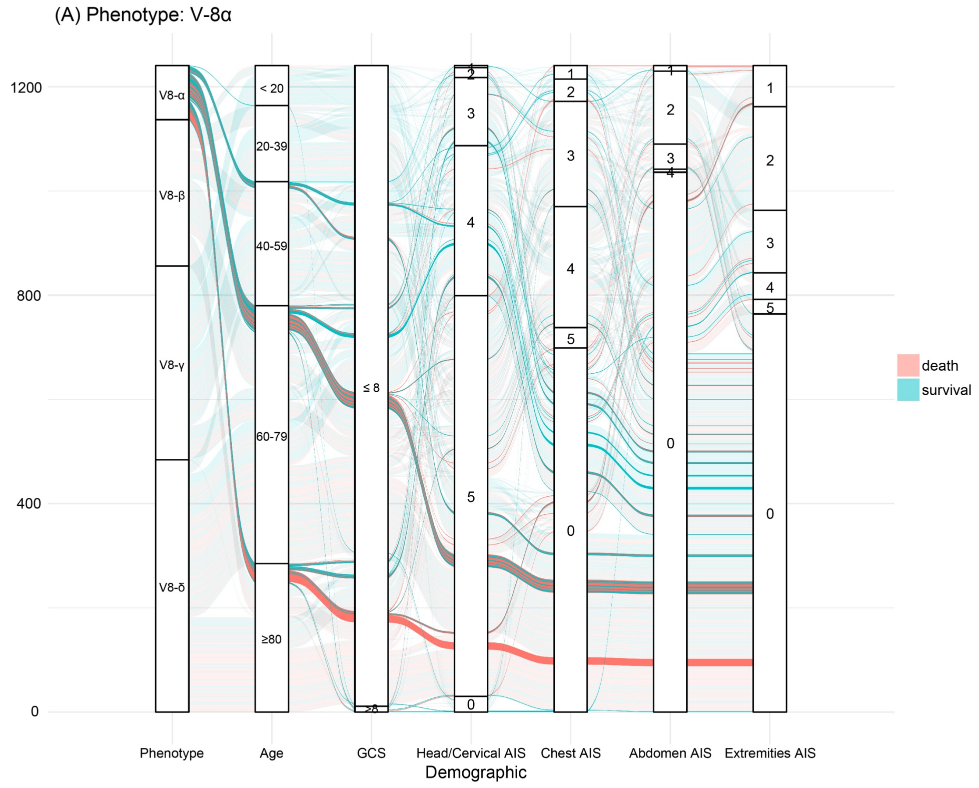

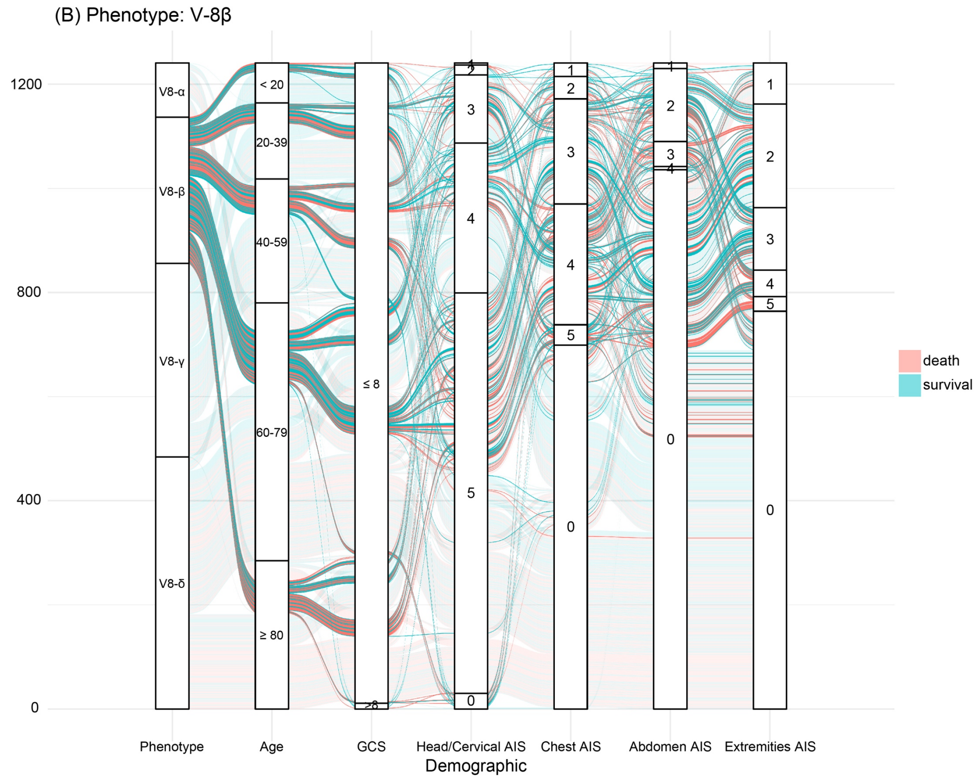

(C) Phenotype: V-8γ

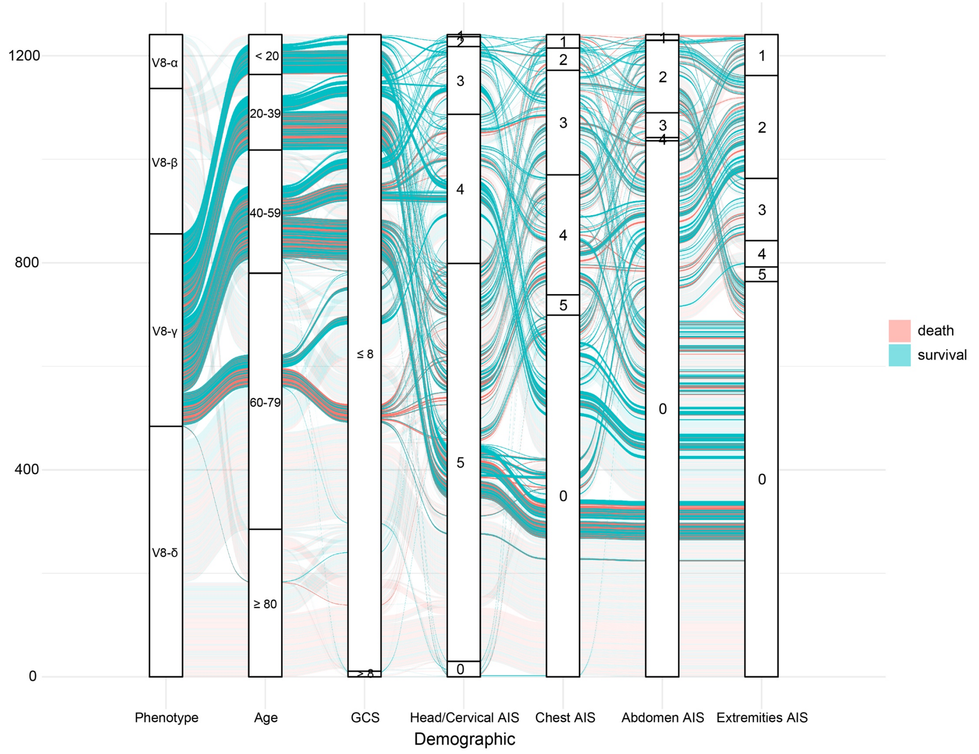

(D) Phenotype: V-8δ

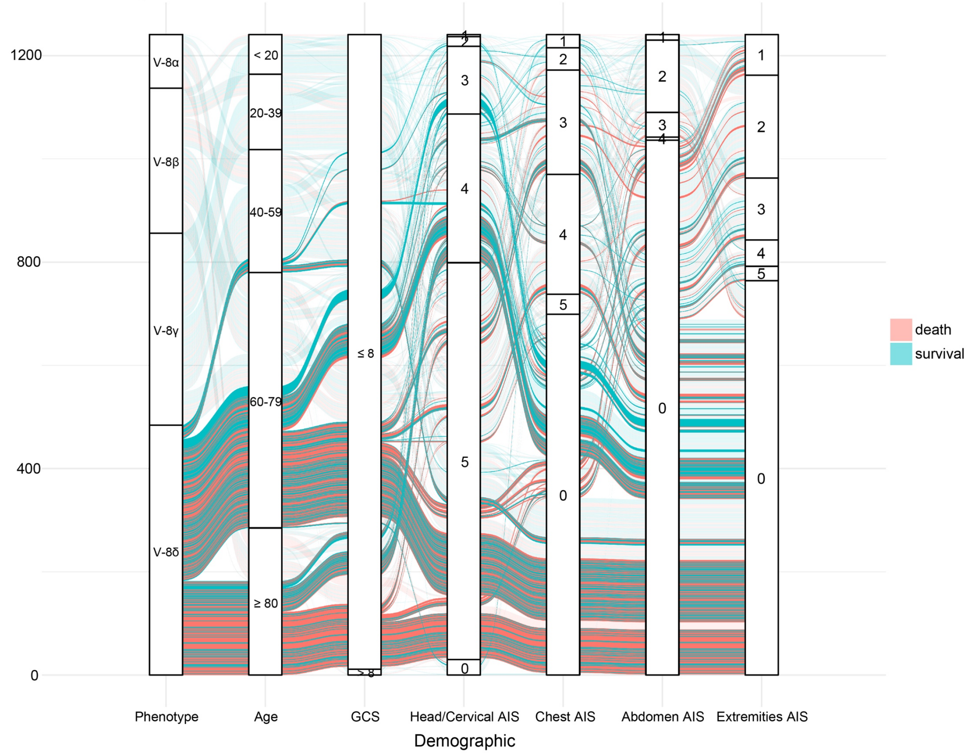

From the left column: clinical phenotype, age, GCS, head/cervical AIS, chest AIS, abdomen AIS, and extremities AIS. The blue bands indicate survivors, and the red bands indicate deceased. The sample sizes of each phenotype: V-8α (*n* = 104), V-8β (*n* = 281), V-8γ (*n* = 372), and V-8δ (*n* = 484). Interpretive example: in these alluvial plots, the members of the phenotype are shown in the leftmost column, and the distribution of each variable is shown in subsequent columns. The V-8β phenotype is more common in younger patients and tends to include multiple traumas. A somewhat distinct band of mortality can be identified in some of the patients with head injuries with an AIS ≥4. AIS, Abbreviated Injury Scale; GCS, Glasgow Coma Scale.

Supplemental Figure.26 Consensus *k* clustering results in the validation cohort

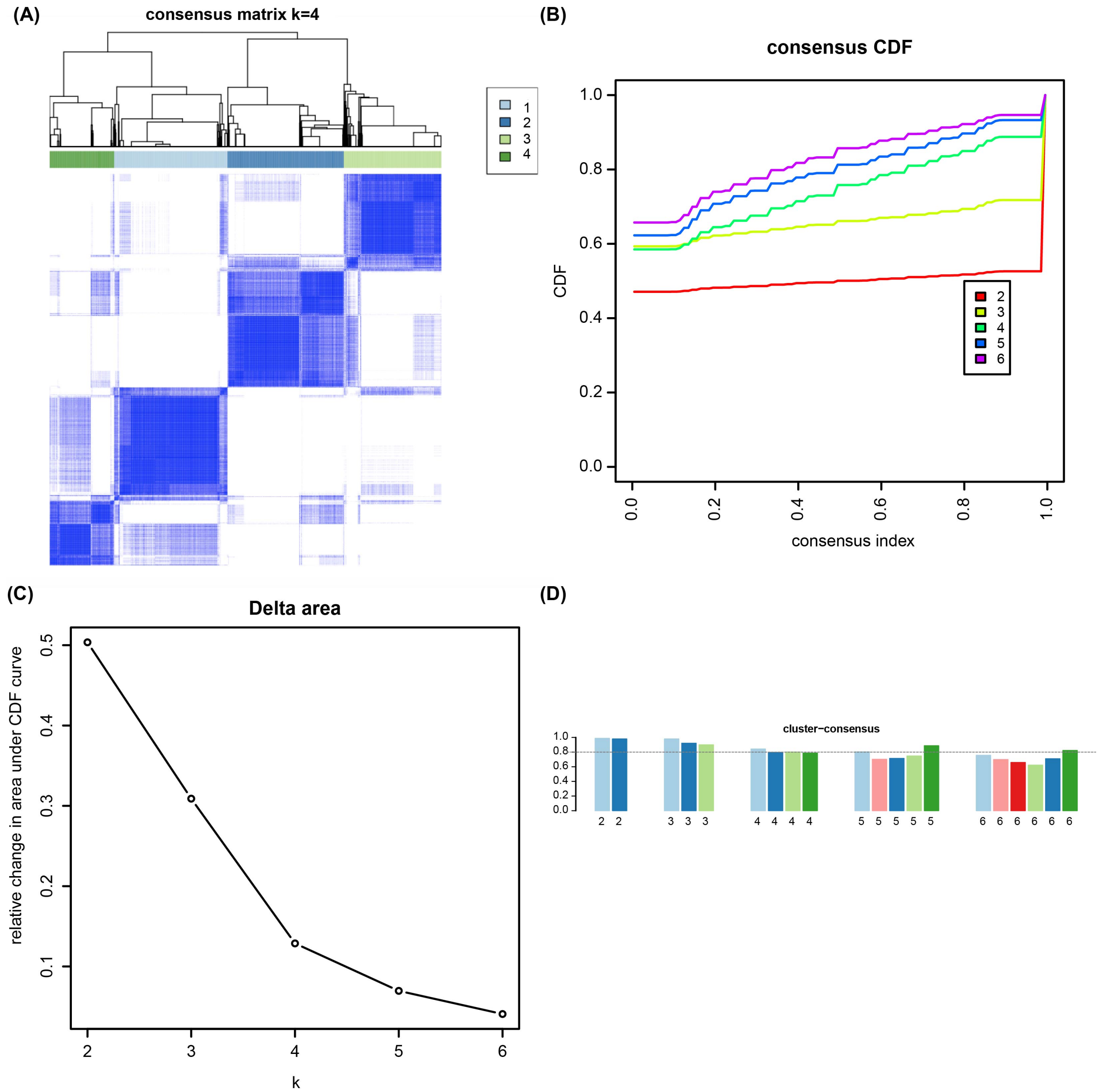

(a) Unsupervised consensus *k*-means clustering in the high-mortality phenotype of the derivation cohort (n = 1,241) and showing optimal partitioning for the $k = 4$ consensus matrix. (b) Consensus cumulative distribution function (CDF) plot across $k = 2$ to $k = 6$, where higher and more horizontal curves suggest an optimal fit. (c) Relative change in the area under the CDF curve with increasing clusters (k), with little change observed from $k = 2$ to $k = 4$. (d) Cluster consensus plot from $k = 2$ to $k = 6$ and showing the mean of all pairwise consensus values between cluster members. A $k = 4$ implied that the consensus value was >0.8 for all clusters and suggesting an optimal fit. Based on the overall judgment of (a)-(d), k=4 was determined to be the optimal cluster.

Supplemental Figure.27 t-SNE plot of clinical phenotype assignments in the validation cohort

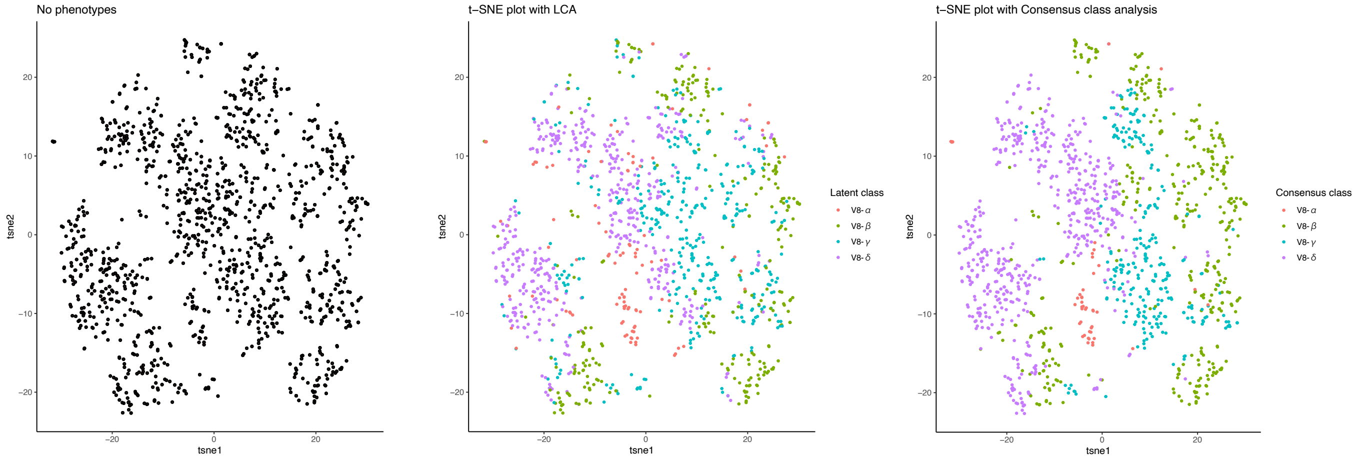

(a) Visualization of phenotypes using the t-SNE method in the high-mortality phenotype group of the derivation cohort. Phenotypes are not shown in color. (b) Color coding based on latent class analysis. (c) Sensitivity analysis using consensus class analysis. Interpretive example: using visualization methods, the phenotype members showed that the clustering results were comparable between the two different methods (latent class analysis and consensus class analysis). t-SNE, t-distributed stochastic neighbor embedding.

Supplemental Figure.28 Kaplan-Meier plot for the validation cohort

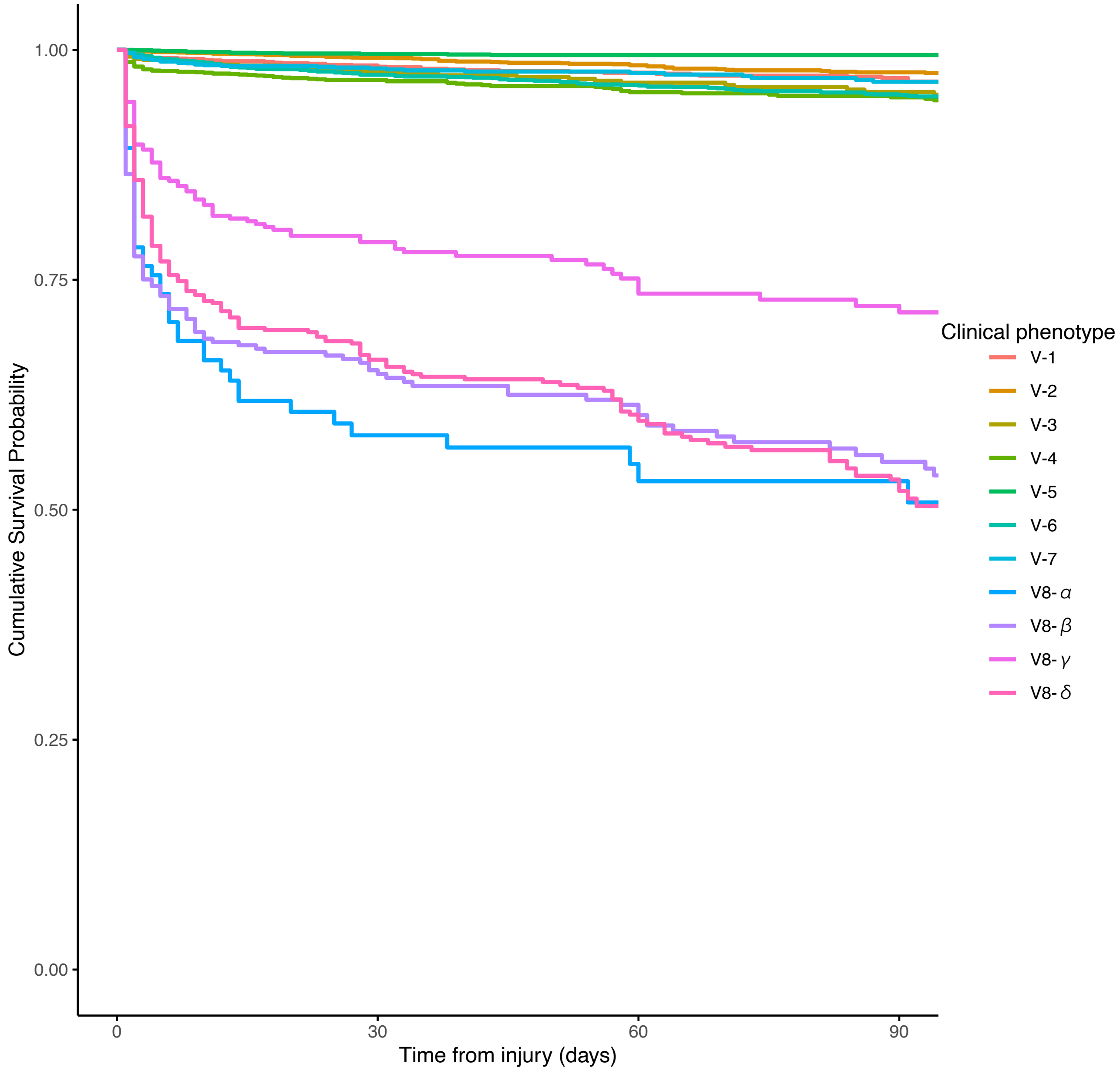

The Kaplan–Meier curves for each phenotype in the validation cohort. The vertical axis shows the cumulative probability of survival, and the horizontal axis shows the number of days from injury to death.

Supplemental Figure.29 Centroid of each cluster determined using principal component analysis in all cohorts

(a)

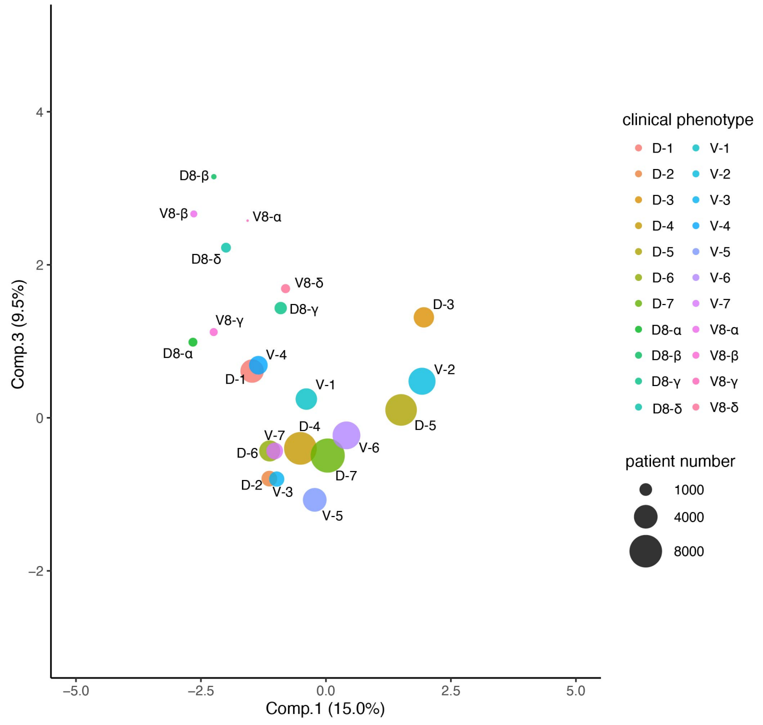

(b)

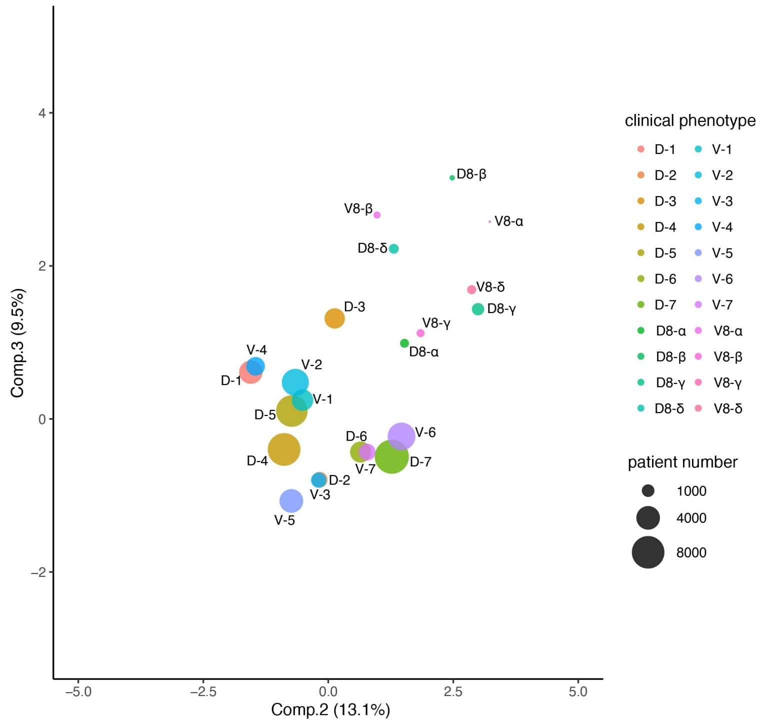

To visualize the homology of the clusters derived from the derivation and validation cohorts, the centroid of each cluster was calculated and shown in primary component analysis (PCA).

(a) The X-axis is the first component, and the Y-axis is the third component. (b) The X-axis is the second component, and the Y-axis is the third component. PCA, principal component analysis. Plot size indicates the number of patients. With respect to cluster 8, which is strongly associated with death, the distribution was clearly different from the remaining clusters 1-7. There was no dissociation in the position of the clusters between the derivation and validation cohorts, suggesting that homology between the cohorts was maintained.
